# Ethnobotanical Study of Indigenous Knowledge on Medicinal Plants Used in the Talensi District of the Upper East Region of Ghana

**DOI:** 10.12688/openresafrica.15807.3

**Published:** 2026-04-10

**Authors:** Emmanuel Azanlerigo, Philip T. Thompson, Cephas Kanati, Mercy Badu

**Affiliations:** 1 Chemistry, Kwame Nkrumah University of Science and Technology Department of Chemistry, Kumasi, Ashanti Region, Ghana

**Keywords:** ethnobotany, medicinal plants, dermatological, gastrointestinal, Talensi District, malaria, snake bite, skin

## Abstract

**Background:**

The Talensi District uniquely combines ritual treatment and traditional medicine for patient care. Despite urbanization, they preserve their lifestyle and extensive knowledge of traditional medicinal plants, distinguishing them from other regions. However, there is no systematic documentation of the Talensi people’s knowledge of these plants in the Upper East Region. This study explored utilization patterns, preparation methods, and the significance of plants in traditional medicine.

**Methods:**

A survey was conducted using semi-structured interviews. Informant Consensus Factor, Fidelity Level, Relative Frequency of Citation, Plant Part Value, and Use Value were selected as key quantitative indices. All analyses utilised GraphPad Prism (version 8.0.1) and Microsoft Excel.

**Results:**

Among 219 informants (115 females, 104 males), most practitioners were aged 51–60 years (53%) and predominantly illiterate (86%). 46 plant species were identified and grouped into four major ailment categories: gastrointestinal disorders, dermatological infections, snakebite, and malaria. Plant parts and preparation methods displayed distinct patterns by ailment category. Roots dominated treatments for skin infections, gastrointestinal disorders, and snakebites, while leaves were mainly used for malaria. Preparation methods varied: boiling was common for malaria and gastrointestinal treatments, while grinding fresh samples characterised snakebite and dermatological preparations. Quantitative ethnobotanical indices indicated high informant consensus factors (ICF) for gastrointestinal disorders (0.97), malaria (0.96), and dermatological conditions (0.93), signaling strong agreement on treatments, with moderate consensus for snakebite (ICF 0.85).

**Conclusion:**

Documentation of the ethnomedicinal species in the Talensi District will not only ensure that traditional medicinal knowledge is preserved and passed on to future generations, but it will also provide their therapeutic usage, potentially leading to the discovery of new drug formulations.

**Abbreviations**
FC : Frequency of Citation
FL : Fidelity Level
ICF : Informant Consensus Factor
RFC : Relative Frequency of Citation
NA : Not Specified
PPV : Plant Part Value
UV : Use Value

## Introduction

Medicinal plants are significant natural resources used over the years to treat different ailments. The Savanna Regions of Ghana, including the Northern part of the country, host a rich diversity of plant life that reflects the region’s climate and ecological conditions. ^1^ Herbal remedies play a vital role in healthcare, deeply rooted in the traditions of the Indigenous communities in the Savanna Regions of Ghana. ^2^ Herbal practitioners, often regarded as custodians of traditional knowledge, use different kinds of plant parts for their healing properties. The Savanna Regions are divided into two main ecological zones: Guinea Savanna and Sudan Savanna. The Sudan Savanna covers parts of the Upper East Region, usually experiencing a lower and shorter rainy season (600–1000 mm). ^3^^–^^5^

Talensi District is among the fifteen districts in the Upper East Region of Ghana. ^3^^–^^5^ The district has rich traditional knowledge of herbal preparations for disease management. Almost every tribe/clan possesses specialized expertise for treating specific ailments. This knowledge historically passed down through generations, now faces threats due to cultural and societal changes.

During ethnobotanical interviews, many herbalists/healers expressed concern that the growing influence of Christianity and Islam has led to a generational shift, causing younger community members to distance themselves from traditional medicinal practices, often perceiving them as incompatible with their religious beliefs (Respondents). This observation aligns with previous research, which highlights the negative effects of modernization, religious influence, and climate change on the continued use of herbal remedies. ^6^^–^^8^ This cultural shift significantly impacts the preservation of essential ethnobotanical knowledge, which could lead to the loss of effective traditional remedies and the biodiversity they support. Usually, important information on the traditional uses of medicinal plants, the various parts used, the mode of application, and, most importantly, information on their efficacy is kept secret. Practitioners only pass on their knowledge to close family members, especially their children.

Therefore, this ethnobotanical study seeks to document the medicinal plants utilized by communities in the Talensi district. The study focused on medicinal plants commonly used to address the area’s four main health issues: snakebites, gastrointestinal disorders (stomach-related ailments), dermatological diseases (skin-related infections), and malaria. These health conditions reflect broader socio-economic and environmental factors that impact public health in the region. ^9^^–^^11^ By systematically recording this knowledge, the study aims to preserve cultural heritage, inform conservation initiatives, and provide useful information for new drug discoveries that could improve healthcare outcomes locally and globally. ^12^

**Figure 6.**
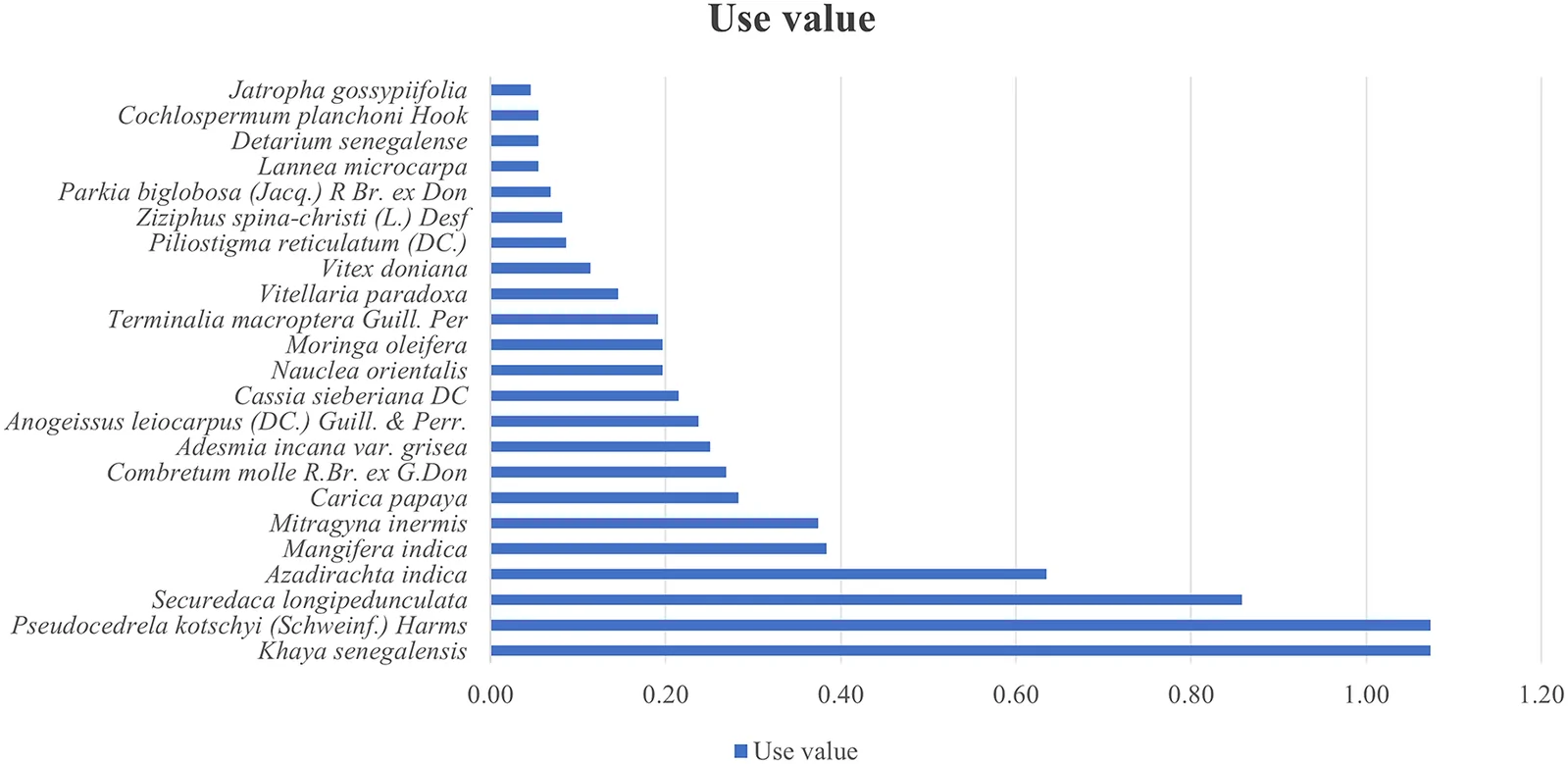
Use value of plant species in the Talensi District.

## Materials and methods

### Location, topography, vegetation cover and climate

The study was conducted in the Talensi District of the Upper East Region, Ghana, from March to September 2024. The district lies within the boundaries of longitudes 0°31′ and 1°05′ west and latitudes 10°35′ and 10°60′ north. The Talensi District, formally Talensi-Nabdam District, was established in 2012, covers an area of 845.3 km ^2^ with a population of 87,021 as of the 2021 census, 102.9/km ^2^ Population Density and 0.65% Annual Population Change from 2010 to 2021. ^3^^–^^5^ Most of the district’s inhabitants (about 90%) are peasant farmers. The topography of the district is generally flat, with relatively undulating lowlands and gentle slopes ranging between 1% and 5%. Elevations range between 100 m and 200 m, with some isolated rock outcrops and uplands mainly around the district capital Tongo and Yinduri reaching 400 m high. Several rivers and tributaries drain the district during the rainy season. In the dry season, most tributaries dry out, leaving only portions of the Red and White Volta Rivers flowing through the district. The area is almost always relatively warm, with temperatures reaching 45°C in March and April and recording a minimum of 12°C in December. ^3^^–^^5^ The prolonged dry season is accompanied by very low relative humidity, which reaches 10% during harmattan (the dry and hazy northeast trade wind) in December and January. With the onset of the rains, it rises gradually to a maximum of 65%. Figure 1 below shows the sketch of the map of the study area.

**Figure 1.**
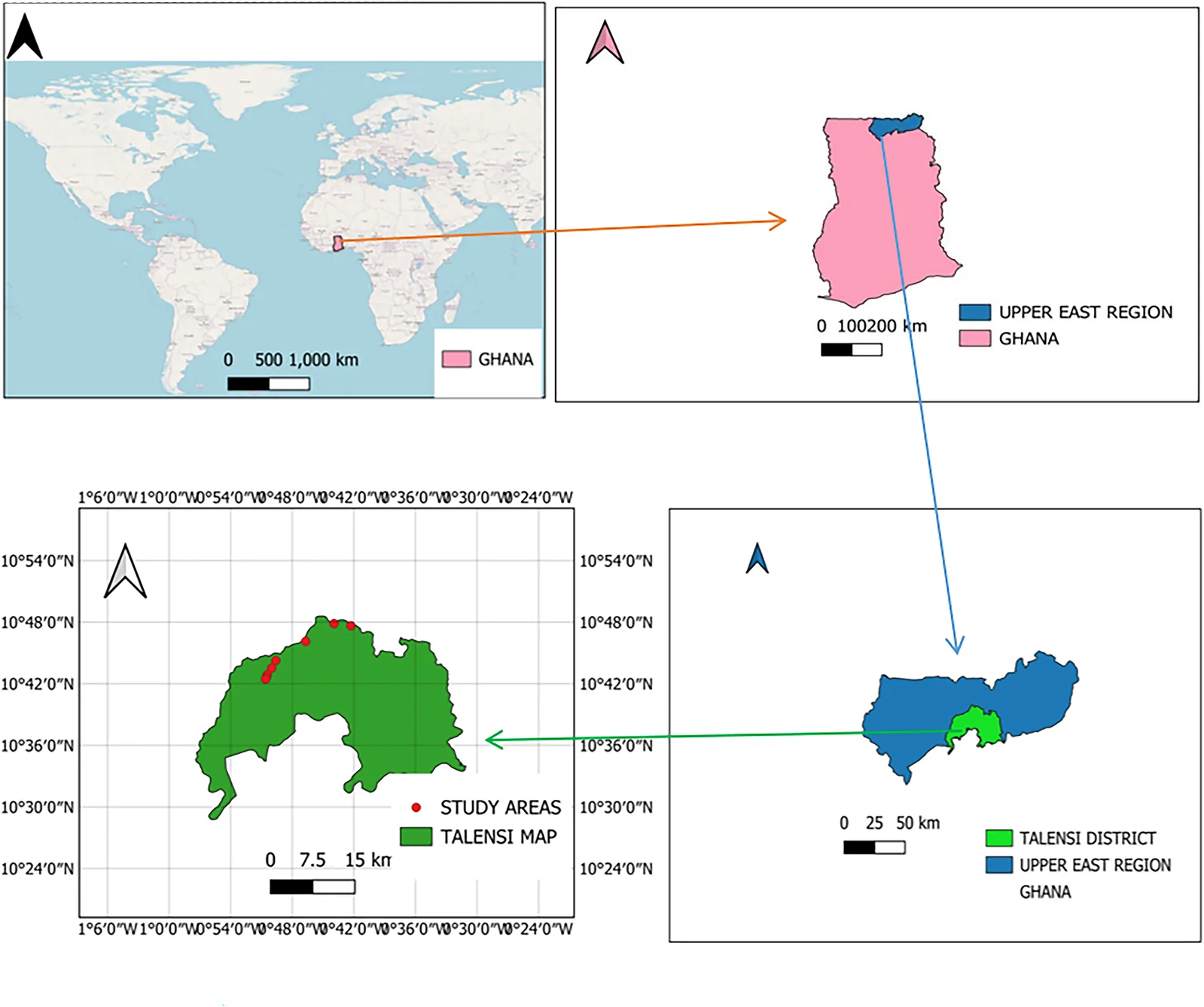
A map showing coordinates of the study area.

### Data collection

Data was collected through semi-structured interviews with 219 respondents aged 30 to over 70. The interviews were conducted in the local Taleni/Frafra dialect. Before each interview, the purpose of the study was explained to informants, and informed consent was obtained from participants. Participants who gave their consent were interviewed, while those who did not provide their consent were not interviewed. Participants signed a written consent form, in cases where participants could not sign, they were assisted and to thumbprint. All consent was administered in the written form. Respondents were selected based on a combination of random and snowball sampling techniques. These methods were used to reduce sampling bias due to gender or specific social group (herbalist) to ensure that the data represent different demographic groups. House-to-house interviews were conducted with individuals within the specified age range, to reduce knowledge bias, which occurs when participants with insufficient expertise provide inaccurate data, reducing the reliability of the study. Thus, informants aged 30 years and above were interviewed as they were believed to possess extensive knowledge of medicinal plants, acquired through years of experience or interactions with elders. Also, the study used some respondents, particularly herbalists, who were recognized for their expertise in medicinal plant use. The study complied with the International Society of Ethnobiology Code of Ethics. ^13^ The questionnaire was divided into five sections, covering demographic information and specific questions about plants used to treat snakebites, stomach-related diseases, skin-related diseases, and malaria.

***Sample size determination and justification***

The number of respondents (n = 219) was determined using Cochran’s sample size formula for social and ethnobotanical surveys. ^14^ Based on the 2021 Population and Housing Census, the Talensi District has an estimated population of 87,021 inhabitants. Using a 95% confidence level (Z = 1.96), a 5% margin of error (e = 0.05), and an estimated population proportion (p = 0.5), the theoretical representative sample for the entire adult population would be approximately 382 respondents. However, because this ethnobotanical study specifically targeted knowledge holders including herbalists, elderly individuals, and community members aged 30 years and above who possess traditional medicinal knowledge, a smaller yet focused sample was deemed more appropriate.

The final number of 219 respondents was therefore considered adequate to capture the diversity of medicinal plant knowledge within the Talensi District, while ensuring feasibility and data reliability. Similar sample sizes have been successfully used in comparable ethnobotanical studies. ^15^^,^^16^

### Plant identification

Plant identification was carried out in two stages. First, the reported medicinal plants were identified in the field with the assistance of knowledgeable local guides and traditional healers. After each interview, the research team, accompanied by local experts, visited nearby forest and fallow areas to locate and preliminarily identify the cited plants.

Subsequently, all collected specimens were taken to the Department of Herbal Medicine, Faculty of Pharmacy and Pharmaceutical Sciences, Kwame Nkrumah University of Science and Technology (KNUST) for taxonomic verification and voucher authentication. At the herbarium, each specimen was examined by qualified botanists and confirmed through comparison with authenticated reference collections and standard botanical literature. Voucher numbers were then assigned to each specimen and deposited at the KNUST Herbarium.

The verified scientific names and corresponding voucher numbers are provided in Tables 3, 4, 5 and 6 of this manuscript. Some voucher specimens had been previously collected and deposited at the herbarium in earlier years, while others were newly collected during this study and assigned current voucher numbers. All vouchers, regardless of collection year, were verified and are maintained at the KNUST Herbarium.

### Data analysis

All analyses were done using the GraphPad Prism (version 8.0.1) and Microsoft Excel. The gathered data was revised, and statistical analysis was conducted.

### Quantitative indices

In this study, Informant Consensus Factor (ICF), Fidelity Index (FI), Relative Frequency of Citation (RFC), Plant Part Value (PPV) and Use Value (UV) were selected as key quantitative indices to assess the reliability, specificity, and cultural importance of medicinal plant use. These indices systematically validate traditional knowledge by measuring informant agreement on plant use (ICF), the specificity of plants for treating particular ailments (FI), their popularity within the community (RFC), and their overall therapeutic relevance (UV). Their application enhances the credibility of ethnobotanical data and helps prioritize species for conservation and pharmacological research. These indices were chosen over others due to their effectiveness in identifying widely recognized medicinal plants with strong cultural and therapeutic significance. ^12^^,^^17^^,^^18^

### Justification for disease categories and application of indices

The ethnobotanical questionnaire was structured around four major disease categories: Stomach-related diseases (stomach pain, ulcers, diarrhea), Skin-related diseases (boils, inflammation, rashes, wounds), Snakebite, and Malaria. These categories were carefully chosen based on both local health relevance and broader biomedical significance. Field observations and informant interviews revealed that these conditions were the most prevalent and culturally prioritized in the study area. Furthermore, these disease groups reflect global public health challenges—notably, snakebite envenomation, ^19^^,^^20^ malaria, ^21^^,^^22^ and antimicrobial resistance (AMR) associated with bacterial infections of the skin and gastrointestinal tract. ^23^^,^^24^ Stomach- and skin-related diseases often arise from bacterial pathogens that have developed resistance to conventional antibiotics, making the search for novel bioactive plants in these categories both locally justified and scientifically strategic.

Focusing on these four categories allowed for detailed and accurate recall by informants and minimized the dilution of data across too many unrelated ailments. Each quantitative index—except the Use Value (UV)—was therefore calculated independently within each disease category, enabling disease-specific assessment of plant importance and consensus. The Use Value (UV) was computed using all plants collectively to evaluate their overall ethnomedicinal importance.

All indices were derived based on scientific nomenclature, although vernacular names were used during field data collection to ensure clarity and cultural accuracy. Each vernacular name was later cross-checked and matched with its corresponding botanical identity using standard floras and herbarium references.

***Plant Part Value.*** The plant part value (PPV) is presented as the percentage of utilized parts of plants (stem, leaves, root, fruit, bark, and flower) as medicinal bioresources. The PPV is calculated according to ^25^^,^^26^ as follows:

PPV(%)=[ΣRU(plantpart)/ΣRU]*100 (1)

∑RU (plant part) and ∑RU represent the sum of the cited plant parts and the number of cited uses for a given plant, respectively. The same was calculated for a mode of administration as well as a mode of preparation for the treatment for each disease category. Results were present in chats for better interpretation.

***Informant Consensus Factor.*** To evaluate the homogeneity of the data, we used the information consistency index (Informant Consensus factor, ICF) for quantitative analysis. ^26^^–^^28^ ICF was calculated using the following formula:

$$\text{ICF}=\text{Σ}({\text{N}}_{\text{ur}}-{\text{N}}_{\text{t}})\text{/Σ}({\text{N}}_{\text{ur}}-1)$$ (2)

Where ICF ranges between 0.00 and 1.00, ∑N _ur_ is the total number of use citations for that category, and ∑N _t_ is the number of plant species used for a specific disease category. Each ICF was calculated independently for the four disease categories to reflect their distinct ethnomedical significance.

***Relative Frequency of Citation.*** Relative frequency of citation (RFC), expressed percentages, was calculated to evaluate the importance of plants used by local healers to treat various diseases using the formula:

RFC=(ΣFC/ΣN)*100 (3)

Where ∑FC is the number of prescriptions mentioning the use of a plant species, and ∑N is the total number of prescriptions in the survey. The values closest to 100 show that nearly all informants mentioned a specific medicinal plant used to treat a particular illness. Low scores show that the usage or purpose of a medicinal plant species is mentioned by few, or occasionally by one informant. ^26^^–^^28^

***Fidelity Level.*** The percentage of the most popular and valuable medicinal plant for a specific condition or use category was calculated using fidelity level (FL) using the formula:

FL(%)=(ΣIp/ΣIu)*100 (4)

Where ∑Ip is the proportion of informants who cited or mentioned using a medicinal plant to treat a specific disease category, and ∑Iu is the total number of informants who cited the plant for any other use or purpose. ^26^^–^^28^ In cases where a species was associated with multiple therapeutic uses, FL was computed separately for each major disease category to capture its primary and dominant indications. This approach ensures that overlapping uses are accounted for without inflating fidelity scores, thereby identifying the plants most culturally and therapeutically specific to a particular condition.

A medicinal plant with a high value will likely have many citations and be the most popular species for treating a specific condition. This ethnobotanical documentation included four ^4^ different disease categories.

***Use Value.*** To determine the relative importance of each medicinal plant, the Use Value (UV) index was calculated following established ethnobotanical methods. ^26^^–^^28^

UV=(ΣUr/ΣN) (5)

∑Ur is the number of use reports, citations, or mentions by each informant for a particular species, and ∑N is the total number of informants who participated in the study. A “use report” was defined as a single mention of a plant species by an informant for the treatment of a specific ailment category.

To avoid overestimation, when a plant was cited under multiple ailment categories by the same informant, only one use report was counted for that individual. UV values were therefore computed collectively across all disease categories to represent the overall cultural importance of each species. Higher UV values indicate more frequently cited and culturally significant species, while lower values denote plants with fewer mentions.

Low numbers signify fewer mentions or citations, whereas high values show a significant volume of use reports or citations from the informants. It counts as one use report or citation each time an informant identifies or describes a species of medicinal plant that is being used to treat a condition or for another reason.

## Results and discussion

### Demographics of informants

Considering the demographics of the study population, it was revealed that most informants were females (115; 55.3%). Detailed results are shown in  Table 1. The observed predominance of female informants may be attributed to the traditional nature of the division of labour practised in the study area. Women are primarily engaged in domestic duties: caregivers, home managers, providing food for the family, caring for the sick, and many other social supports. They also primarily engage in farming and trading and thus are exposed to many valuable plants in their communities. Most of the informants have little or no formal educational background. The majority of the informants practice traditional religion (60%), followed by Christianity (32%), Islam (3%), and unspecified (5%). Age distribution for most informants ranged from 51–60 years (n = 117, 53%), followed by those aged >70 years (n = 42, 19%), and 30–50 years (n = 60, 23%). Informants under 30 years were not recorded, as they are believed to have little to no knowledge of traditional herbal treatments, often expressing a lack of interest in traditional medicine during interviews. This agrees with other reports in the literature. ^29^^–^^31^

**Table 1.** Demographic information of informants.

Parameter	Category	Number_of_ informants	Percent_ informants
**Gender**	Male	104	47
	Female	115	53
**Age (in years)**	30–50	60	23
	51–60	117	53
	Above 70	42	19
**Educational level**	None	188	86
	Elementary	9	4
	Junior high	8	4
	Secondary	6	3
	Tertiary	8	4
**Religion**	Christian	71	32
	Islamic	6	3
	Traditionalist	131	60
	Others	11	5
**Occupation**	Civil servant	7	3
	Trader	42	19
	Farmer	166	76
	NA	4	2

*Note:* NA = Not specified.

### Plant part value

The selection of a particular plant part for remedy preparation varies, depending on the disease being treated. This is shown in Figure 2, a graphical representation of the percentage of citations of the plant parts used to treat the various disease categories. Choosing a particular plant part reflects a deep understanding of the medicinal properties associated with different plant tissues. The preference shows an interplay between traditional knowledge, perceived efficacy, and sustainable harvesting practices.  Figure 3a,  Figure 3b,  Figure 3c and  Figure 3d, show photos of plants for treating various disorders, including snake bite, dermatological infections, malaria and stomach-related disorders, respectively.

**Figure 2.**
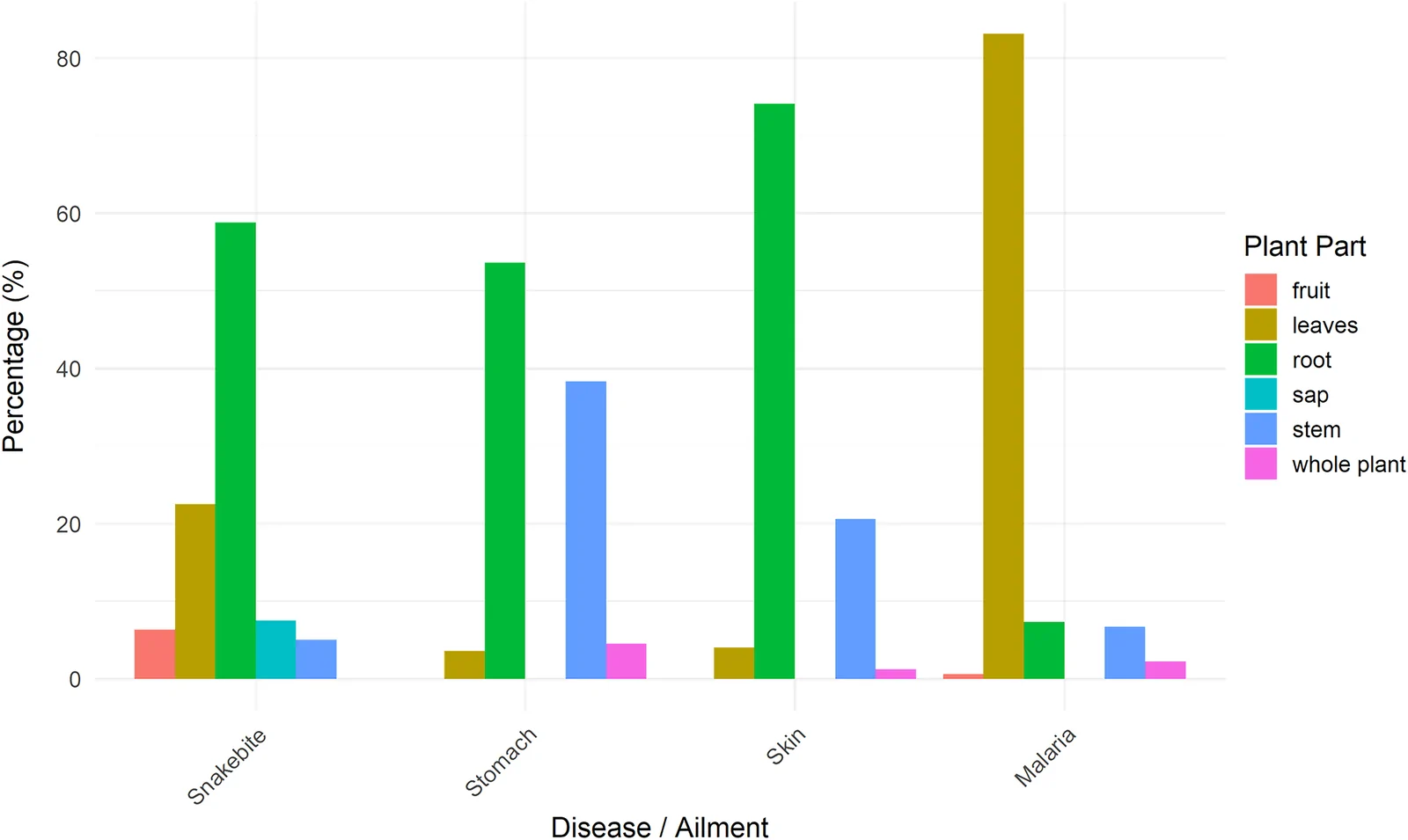
Plant part cited for each ailment.

**Figure 3a.**
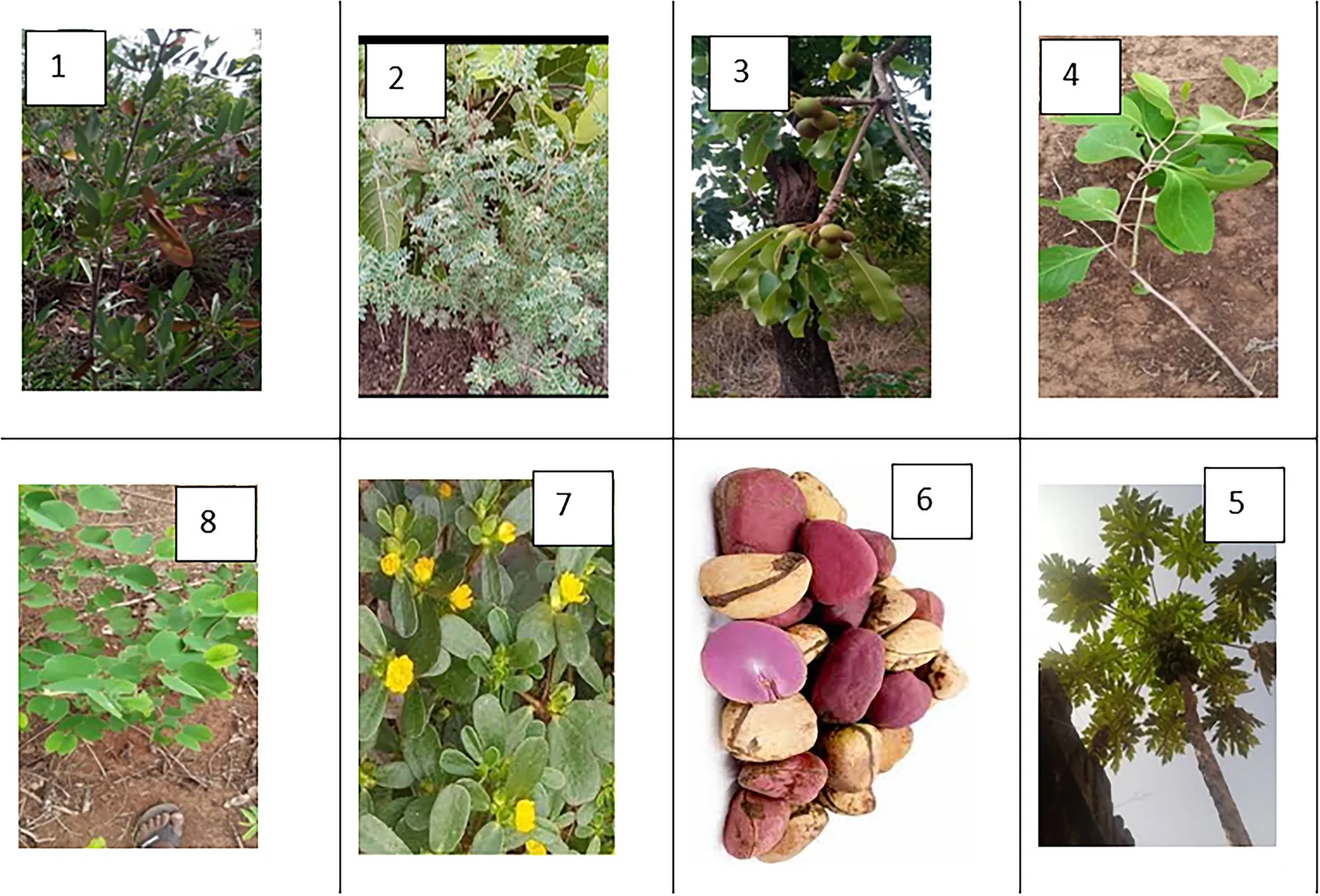
1–8: Images of sample plants/plant part used to treat snake bite.

**1** ( *Securedaca longepedunculata*), **2** ( *Adesmia incana var. grisea*), **3** ( *Vitellaria paradoxa*), **4** ( *Ziziphus spinachristi*), **5** ( *Carica papaya*), **6** ( *Cola nitida*), **7** ( *Portulaca oleracea Linn*) and **8** ( *Piliostigma reticulatum*).

**Figure 3b.**
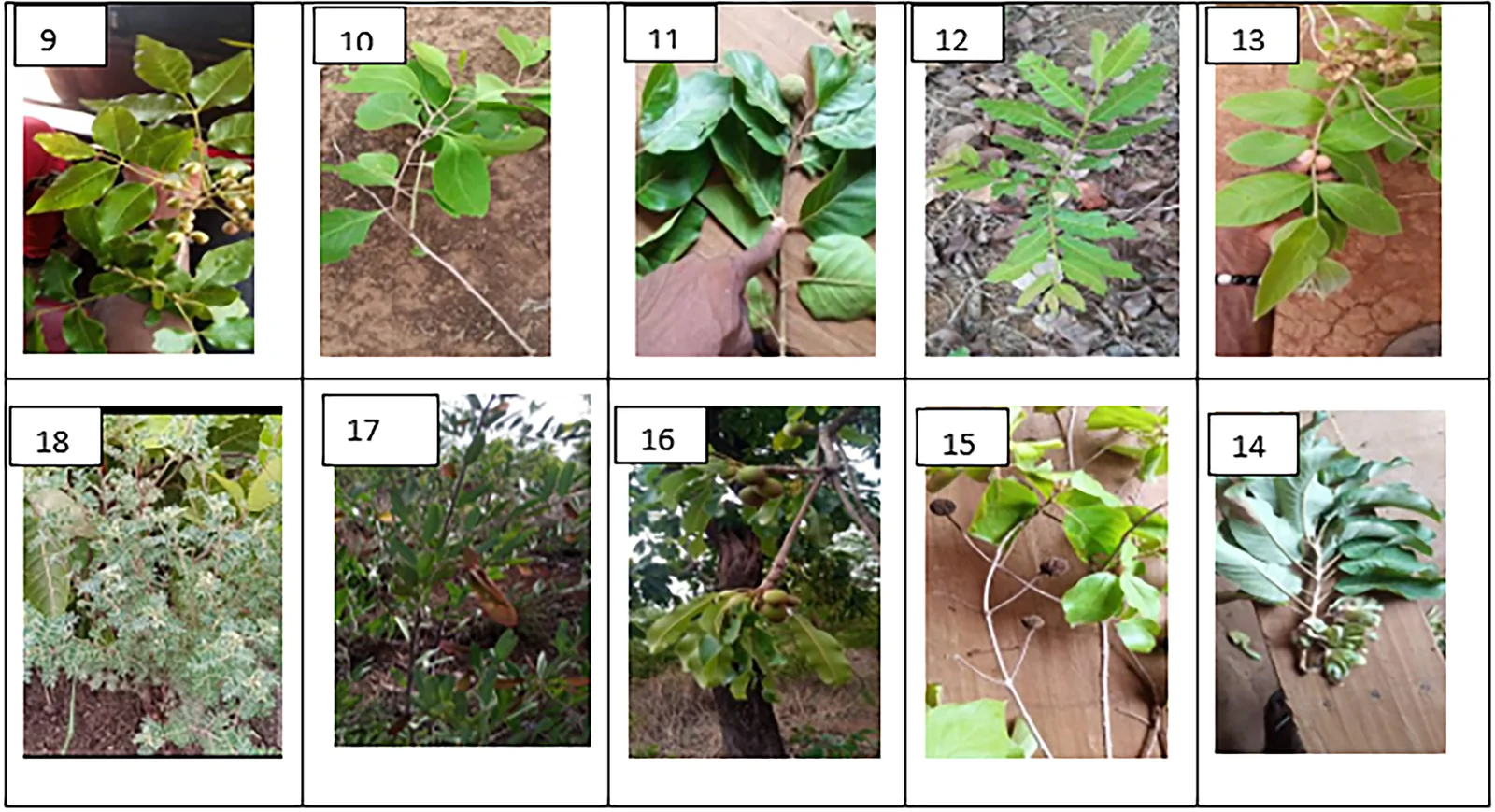
9–18: Sample photos of plants used for treatment of dermatological infections.

**9**( *Lannea microcarpa*), **10**( *Ziziphus spina-christi*), **11**( *Nauclea orientalis*), **12**( *Pseudocedrela kotschyi*), **13**(Combretum molle), **14**(Terminalia macroptera), **15**( *Mitragyna inermis*), **16**( *Vitellaria paradoxa*), **17**( *Securedaca longepedunculata*) and **18**( *Adesmia incana*).

**Figure 3c.**
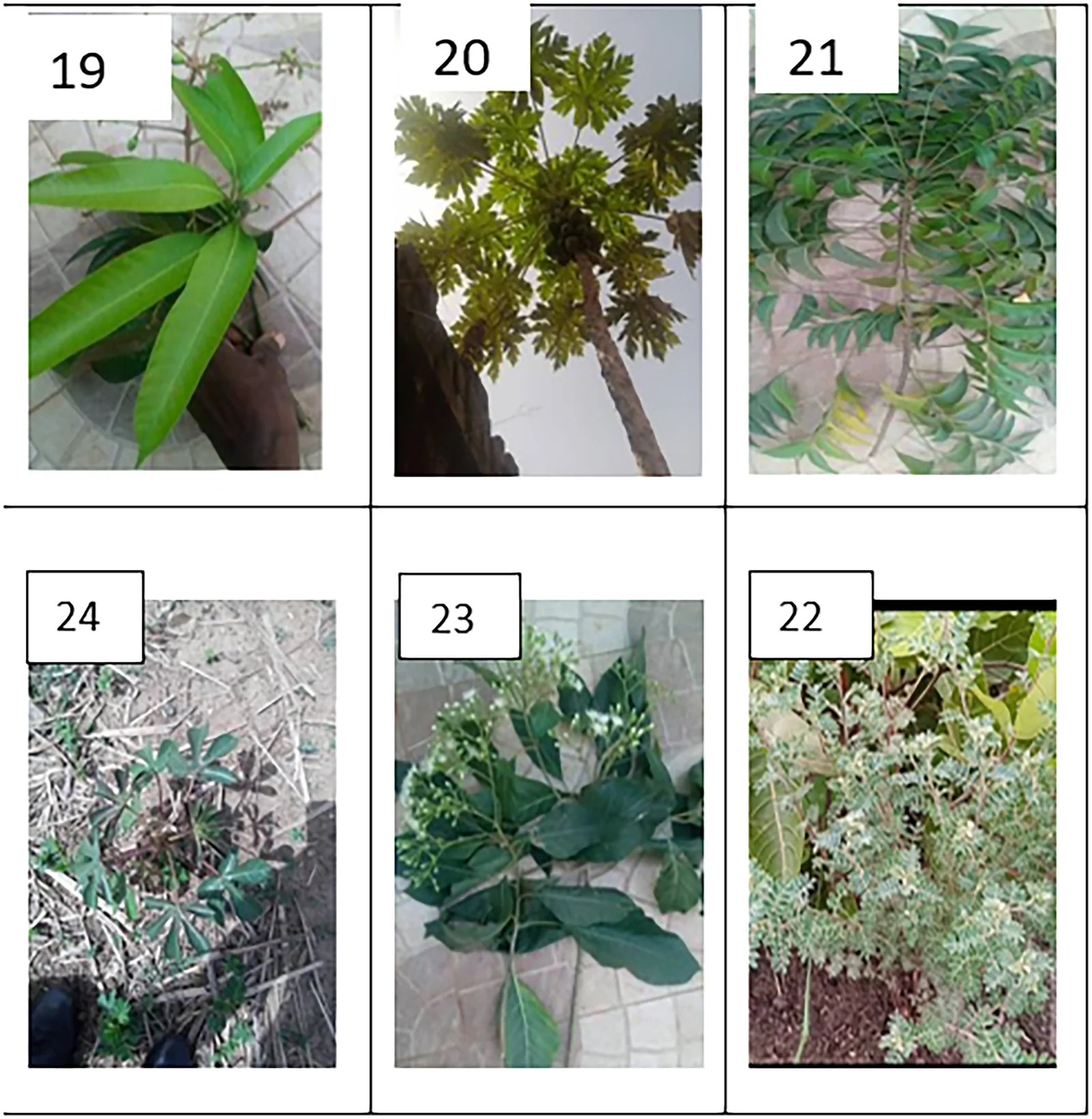
19–24: Sample of photos of plants used for malaria treatment.

**19**( *Manifera indica*), **20**( *Carica papaya*), **21**( *Azadirachta indica*), **22**( *Jatropha gossypiifolia*), **23**( *Vernonia amygdalina*), and **24**( *Adesmia incana*).

In the treatment of snakebite, most of the respondents cited similar plants and the root was the most frequently cited plant part (58.8%), followed by leaves (22.5%), stem (5.0%), sap (7.5%), and fruit (6.3%). For stomach-related diseases, the roots were the most cited part (53.6%), followed by stems (38.3%), whole plants (4.5%), and leaves (3.6%). For skin-related diseases, again, the roots were predominantly cited (74.1%), followed by the stems (20.6%), leaves (4.0%), and whole plants (1.3%). The predominance of root usage aligns with other ethnobotanical study reports in Africa, where roots are traditionally believed to contain higher concentrations of bioactive compounds, particularly alkaloids and other secondary metabolites essential for treating acute conditions like snakebites. ^32^^–^^34^ Plant roots have been described as storage organs and accumulate bioactive compounds in higher concentrations, making them a preferred choice for life-threatening conditions requiring potent remedies. ^35^

In the case of malaria treatment, the leaves were overwhelmingly preferred (83.1%), followed by root (7.3%), stem (6.7%), whole plants (2.2%), and fruit (0.6%). Respondents explained that they can prepare decoctions, infusions, and steam baths for their treatment regimens by using the leaves. This usually gives them immediate relief. Referring to the literature, most reports on antimalaria compounds are typically obtained from the leaves of plants. ^36^^,^^37^ Leaves are also a sustainable resource, as their harvesting generally does not compromise plant survival, unlike root extraction. ^38^

### Methods for remedy preparation

The methods for preparing herbal remedies from the selected ailments as recorded in the study revealed how people perceive the preparations’ pharmacology and traditional drug delivery strategies.  Figure 4 shows the percentage citation of the mode of preparation of the plant for each ailment category. Data from the study shows that stomach-related diseases usually employ boiling/decoction as the dominant preparation method, accounting for 76.5% of the remedies, followed by soaking in water/infusion (11.5%), grinding into powder (9.1%), and chewing/mastication (2.9%). The preference for boiling indicates a reliance on water-soluble bioactive compounds, with heat enhancing their extraction and enhance promoting their bioavailability. Additionally, administering remedies at high temperatures (warm) aligns with the traditional belief in the efficacy of hot decoctions for internal ailments, as reported in the literature. ^38^^,^^39^

**Figure 3d.**
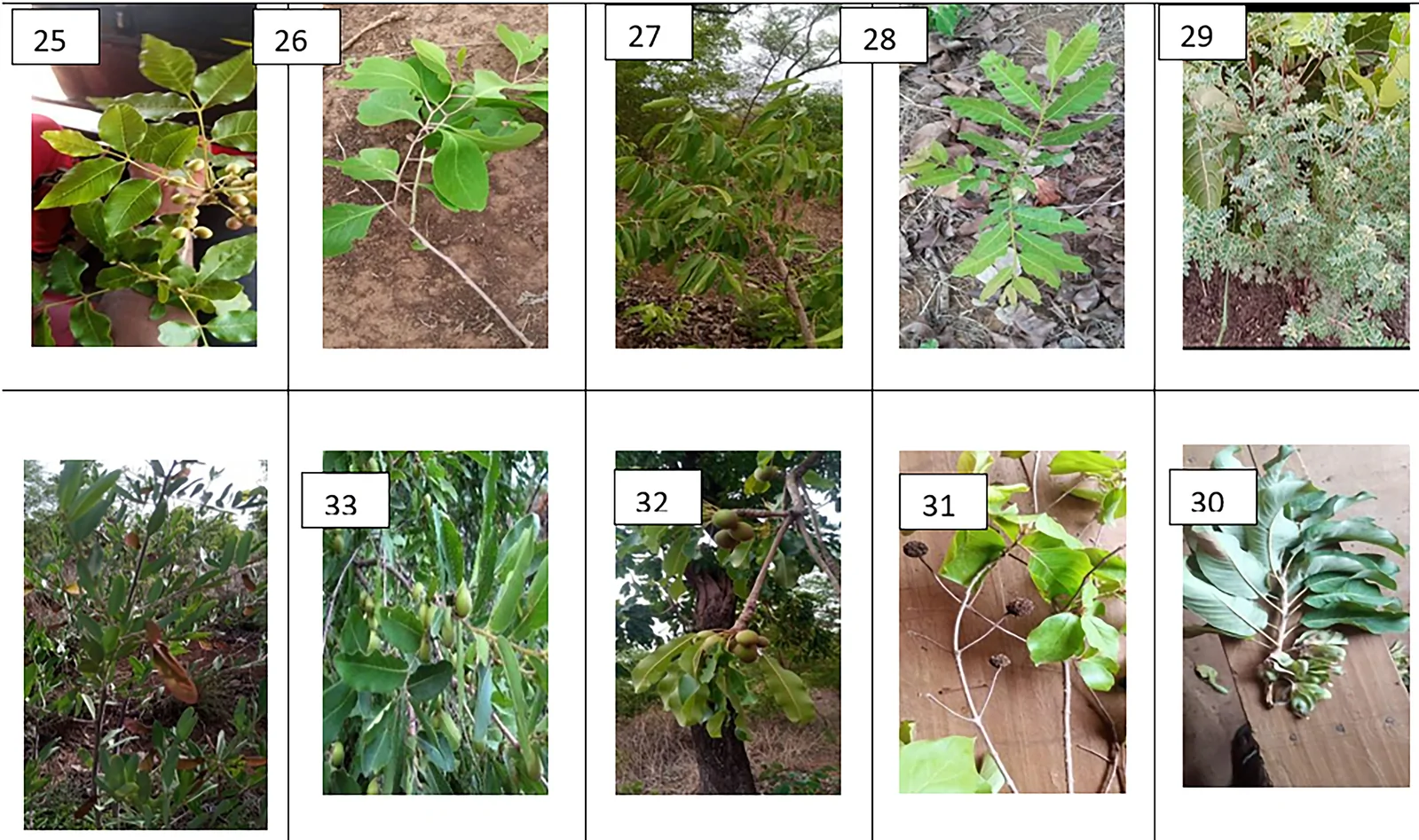
25–34: Sample photos of plants used for treatment of stomach related diseases.

**25**( *Linnea microcarpa*), **26**( *Ziziphus spinacristi*), **27**( *Combretum molle*), **28**( *Pseudocedrela kotschyi*), **29**( *Adensmia incana var.grisea*), **30**( *Terminalia macroptera*), **31**( *Mitragyna inermis*), **32**( *Vitellaria paradoxa*), **33**( *Diospyros mespiliformis*) and **34**( *Securidaca longipedunculata*).

For skin-related disease treatment, grinding material into powder is the primary preparation method, representing 85.5% of the cases, followed by boiling/decoction (10.9%), soaking in water/infusion (1.8%), and squeezing out liquid/extraction (1.8%). Grinding increases the surface area of the material, hence accelerating the interactions between the bioactive compounds and the disease-causing organisms. This facilitates the absorption of drug compounds when applied topically. This approach aligns with traditional practices in dermatology, where powdered or paste-like formulations are preferred for treating skin conditions due to their ease of application and localized action. ^40^

For malaria treatment, boiling/decoction was also the most utilized method, accounting for 94.8% of remedies, with soaking in water at ambient temperature recording 4.9% of cases and burning accounting for 0.6%. However, according to the respondents, burning is more of preive method than treating as it thrives mosquitoes away and prevent bite. The highly accepted use of boiling indicates its effectiveness in extracting heat-stable, water-soluble compounds. These compounds could include flavonoids and alkaloids, which are known to possess antimalarial properties. ^41^ The preference for warm decoctions further explains their perceived efficacy, which may also be tied to traditional medicinal practices across different cultures.

Methods for preparing remedies for snakebite were more diverse, reflecting the urgency of treating this life-threatening condition. Grinding fresh plant material into powder/paste before use was the most common method (40.0%), followed by squeezing out liquid/crushing (25.3%), burning (6.7%), chewing/mastication (5,3%), and soaking in water (6.7%). These methods are geared towards rapid extraction of active compounds for immediate use. Similar approaches have been documented in other ethnopharmacological studies on snakebite treatments, emphasizing the importance of swift preparation and administration. ^42^^,^^43^

### Mode of administration

This study investigated the method of administering the herbal preparations based on three main administration routes: oral, topical, and steam bath. The results are shown in  Figure 5.

**Figure 4.**
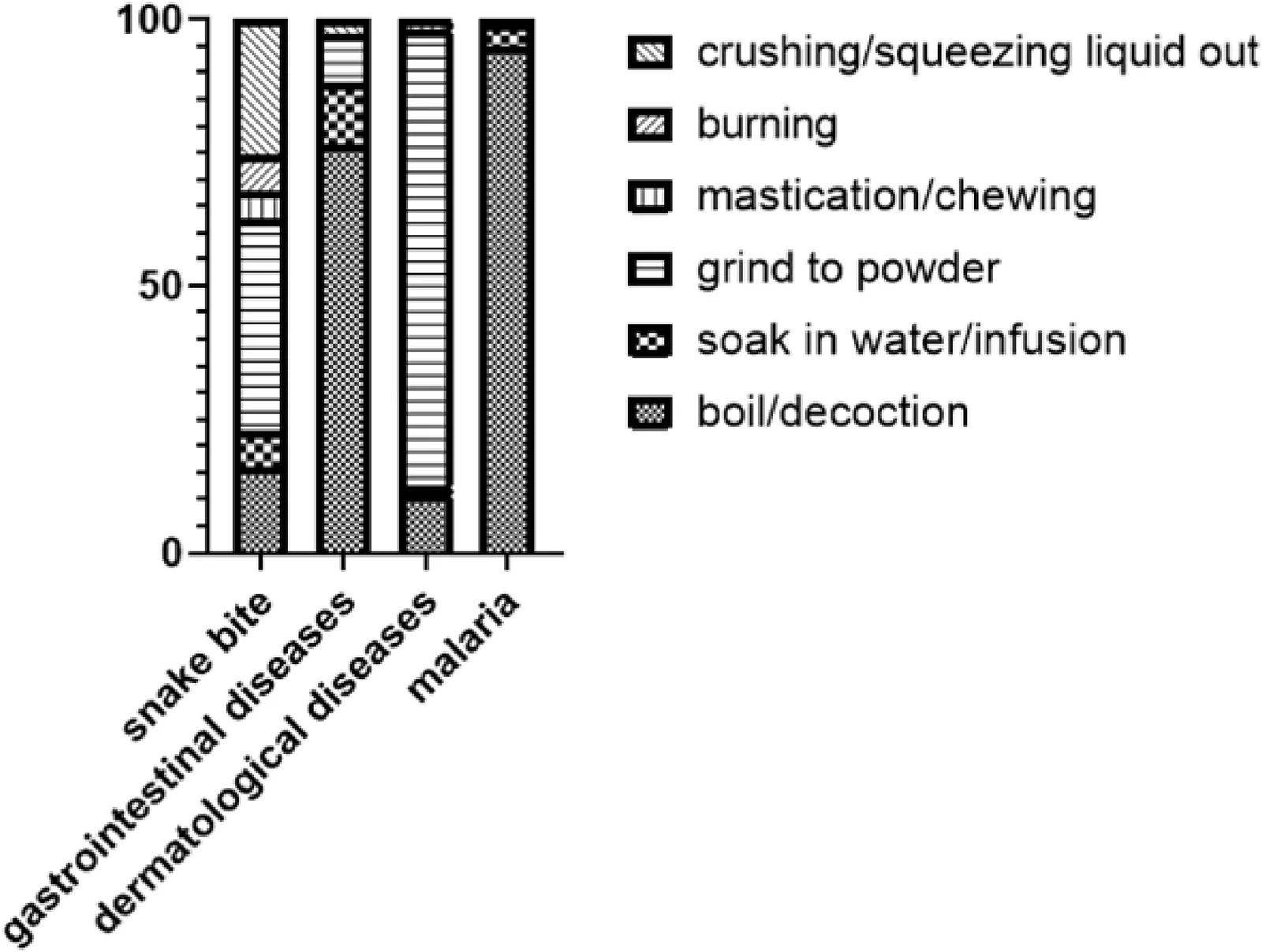
Percentage citation of the mode of preparation of the plant for each ailment category.

Oral administration was observed to be the preferred method for stomach-related diseases, cited by 97.3% of respondents, with only 2.3% opting for topical application. As indicated by the study results, the oral preference shows the recognition that gastrointestinal conditions usually require systemic treatment; hence, oral delivery may give direct access to the bioactive compounds. Again, the use of decoctions and infusions prepared from medicinal plants is based on the understanding that oral administration is effective for conditions involving the digestive system, which facilitates direct absorption of compounds into the gastrointestinal tract. ^44^^,^^45^

Topical application dominated the mode of administration for skin-related diseases (88.6%), while oral administration was less frequently cited (11.4%). The need for localized and direct application to the target influences this trend. Most traditional dermatological practices in various cultures use topical application. ^40^

In the case of malaria treatment, steam bath and oral applications were cited by 51.3% and 48.7% respectively. Steam bath applications are assumed to address external symptoms like fever and body aches. At the same time, oral remedies will target systemic effects such as the incidence of parasite attacks. This strategy provides a holistic approach to treating malaria. Reports indicate that steam inhalation and herbal baths are common for febrile illnesses. ^46^^,^^47^

For snakebite treatment, topical application was the most cited (62.1%), while oral administration accounted for 37.9%. The topical approach is critical considering the time effect and the urgency of treating snakebites to ensure a quick neutralizing of the venom at the bite site. Using plant-based anti-inflammatory, anticoagulant, or venom-neutralizing properties has been reported as an effective management system for snakebite. ^33^^,^^34^^,^^42^ Even though oral administration was less cited, oral remedies are believed to address systemic damage, such as pain or inflammation.

### Informant Consensus Factor

The Informant Consensus Factor (ICF) is an important tool used in ethnobotanical studies to provide quantitative information on the degree of agreement among informants, especially on using plants to treat disease categories. This study considered the ICF of all the studied diseases, and the data obtained are shown in  Table 2.

**Table 2.** Informant Consensus Factor of the various disease categories.

Category.of. Diseases	Nu	Nt	Informant.Consensus.Factor
Snake bite	88	14	0.89
Stomach related ailments/gastrointestinal disorders	788	29	0.96
Dermatological disorders/skin related diseases	357	26	0.93
Malaria	334	15	0.96

The Stomach and Gastrointestinal diseases category and malaria achieved the highest ICF value of 0.97 and 0.96 respectively, indicating a strong consensus among informants about the plants employed for such treatments, followed by the skin disorders with an ICF value of 0.93. The high agreement suggests a shared traditional knowledge base and confidence in the efficacy of the selected plants for treating stomach-related, skin-related, and malaria issues. Snakebite gave an ICF value of 0.85, representing a relatively low consensus among informants. The lower agreement reflects the varied knowledge and practices across different families and tribes. This divergence is likely due to the localized and specialized nature of snakebite treatment, often handled by specialists, locally known as *“waakolog daan”.* These specialists employ a combination of herbal remedies and ritual practices, emphasizing the complexity and unique approaches to this form of treatment.

### Relative Frequency of Citation and Fidelity Level

Fidelity Level (FL%) and Relative Frequency of Citation (RFC%) were calculated to assess the ethnobotanical significance of species. FL% measures the consensus on a plant’s use for a specific ailment, while RFC% indicates overall reliance on a species based on citation frequency. Comparing these indices helps differentiate widely used species from those with specialized applications. In this study, RFC% values ranged from 1.2% to 46.5%, with higher values reflecting more significant reliance on species, while lower values may indicate less common but potentially specialized uses.  Table 3 below shows %RFC, number of citations, %FL of plants used for treating snake bite and other diseases, plants that can treat and side effects, if any.

**Table 3.** Information of plants used for the treatment of snakebite.

Voucher number	Medicinal plant	Family	Local name	Citations	RFC	FL	Other uses	Side effect
KNUST/HM1/2023/R006	*Securidaca longepedunculata* Fresen.	Polygalaceae	Pelig	41	46.6	20.9	Boil, inflammation, pains, insect and snake repellant, stomach pains, pains in bones, deliver placenta, body weakness, de-clot blood after birth	Yes
KNUST/HM1/2023/F009	*Carica papaya* L.	Caricaceae	Pawpaw	3	3.4	4.9	Fever	Yes
KNUST/HM1/2016/SB013	*Piliostigma reticulatum* (DC.) Hochst.	Fabaceae	Ba-an	13	14.8	68.4		No
KNUST/HM1/2018/L052	*Vitellaria paradoxa* C.F. Gaertn.	Sapotaceae	Ta-an	10	11.4	31.3	Diarrhea, kokoo	No
KNUST/HM1/S002	*Cola nitida* (Vent.) Schott & Endl.	Sterculiaceae	Kolanut	4	4.5	100.0		
KNUST/HM1/2025/SB013	*Khaya senegalensis* (Desr.) A. Juss.	Meliaceae	Kok	2	2.3	0.8	Stomach pains, inflammation	No
KNUST/HM1/2025/L026	*Adesmia incana var. grisea Hook. f.*	Fabaceae	Kbantiug	4	4.5	7.3		No
KNUST/HM1/2025/L024	*Mitragyna inermis* (Willd.) Kuntze	Rubiaceae	Yeblig	1	1.1	1.3	Provides strength for pregnant women	No
KNUST/HM1/2025/L026	*Cassia sieberiana* DC.	Fabaceae	Kolkbawure	1	1.1	2.2	Diarrhea	No
KNUST/HM1/2025/SB012	*Pseudocedrela kotschyi* (Schweinf.) Harms	Meliaceae	Shiarig	4	4.5	1.7	Stomach pains, inflammation	No
KNUST/HM1/2025/L028	*Ziziphus spina-christi * (L.) Desf.	Rhamnaceae	Lien	1	1.1	5.9		No
KNUST/HM1/2017/L005	*Lannea microcarpa* Engl. & K. Krause	Anacardiaceae	Sisablik	1	1.1	9.1		No
KNUST/HM1/2025/L016	*Adansonia digitata* L.	Malvaceae	Tuu/Tua	2	2.3	66.7		No
KNUST/HM1/2019/L019	*Portulaca oleracea* L.	Portulacaceae	Baatobzeriba	1	1.1	100.0		No

***Plants for snakebite treatment.*** From the data obtained, *Securidaca longepedunculata* (locally known as Pelig) was the most cited plant, with 41 citations, representing the highest RFC (46.5%). Interview responses indicated that the root bark of this plant is widely used for snakebite treatment, with various applications including placing dried root bark around homes to repel snakes, applying powdered root bark topically to snakebite wounds, and consuming a decoction orally to counteract venom effects. Some respondents also reported chewing the root bark to mitigate snakebite symptoms. Beyond its primary use for snakebites, the plant was reported to treat headaches, inflammation, labor induction in animals, postpartum care, and blood clotting disorders. This agrees with other studies in which *S. longepedunculata* has been documented for its anti-inflammatory, analgesic, and antimicrobial properties. ^48^^–^^52^ Additionally, the high FL% values observed for the plant support its extensive use in snakebite management and highlight the need for further pharmacological validation. The next most cited species was *Piliostigma reticulatum* (locally called Ba-an), with 13 citations (15.1% RFC). Similarly, *Piliostigma reticulatum* has been found to possess antioxidant and antimicrobial properties, which may contribute to its traditional use in treating snakebites and wounds. ^53^ *Vitellaria paradoxa* (locally called Ta-an), with 10 citations (11.6% RFC). The survey revealed that the sap of *V. paradoxa* is applied immediately after a snake bite to remove the teeth of the snake and reduce the effect of the venom. This agrees with reports where *Vitellaria paradosa* and *Securidaca longepedunculata* were part of the plants mentioned for snake bite treatment. Aside from ethnobotanical alignment, *V. paradoxa* is also well documented for its wound-healing and anti-inflammatory effects, reinforcing its therapeutic significance. ^33^^,^^34^^,^^54^^,^^55^

Other species, including *Cola nitida* (locally called Kolanut), showed a high FL% (100%), indicating complete agreement among informants on its use for snakebites, suggesting strong cultural consensus and potential efficacy. ^33^^,^^56^ *According to the respondents, C. nitida is chewed to reduce the impact of the snake bite. Khaya senegalensis (locally called Kok) had a low FL% (0.4%), reflecting limited agreement among informants. This* could indicate variability in usage or limited ethnobotanical knowledge regarding its effectiveness in snakebite treatment.

***Plants for stomach-related diseases.*** From the study, *K. senegalensis* (locally called Kok) recorded the highest RFC (18.8%) and a moderately high FL% (63.4%), underscoring its widespread use and versatility in treating multiple conditions, including stomach ailments, anthrax, and wound healing. This aligns with studies by, ^57^^,^^58^ which report the antimicrobial and wound-healing properties of *K. senegalensis*, supporting its traditional use. *Pseudocedrela kotschyi* (locally called Shiarig/Shiarit) is ranked second with an RFC of 16.5% and an FL% of 55.7%, indicating its cultural and therapeutic significance. Its documented applications in treating inflammation and microbial infections ^59^^,^^60^ corroborate the findings of this study., *Mitragyna inermis* (locally called Yeblig) also displayed a notable RFC of 9.7% and a high FL% of 93.9%, reflecting its frequent use and strong agreement among informants regarding its efficacy. Its use as a remedy for stomach-related issues, swollen legs, and waist pains, as well as its role as an appetite booster, highlights its therapeutic versatility. *Combretum molle* (locally called Gberig) had an RFC of 7.3% and a high FL% of 98.3%, indicating its specific and reliable use in stomach ailment treatments. Previous studies, such as those, have documented its antibacterial and antifungal properties, ^61^^,^^62^ which may contribute to its traditional use.

Species such as *Nauclea orientalis* (locally known as Gu-un) and *Anogeissus leiocarpus* (locally known as Cii) exhibited moderate RFC values (4.5% and 6.2%, respectively) but high FL% values (83.7% and 94.2%). This combination suggests they are used less frequently but highly specifically for stomach-related conditions. Their roles as additives in traditional treatments for snakebites and toothaches reflect their broader medicinal applications.

Certain plants, such as *Detarium senegalense* (locally known as kbarig) and *Cochlospermum planchoni* (locally known as bugla), achieved perfect FL% values (100%) despite low RFCs (1.5% and 1.5%, respectively). These results imply these plants are not widely used but are highly specific to stomach-related treatments within the community. Such specificity could be attributed to cultural practices or the unique pharmacological properties of these plants. *Piliostigma reticulatum* (Ba-an), with a low RFC (0.6%) and a moderate FL% (26.3%), suggests limited use and consensus, possibly due to a broader range of applications beyond stomach-related ailments. Similarly, *V. paradoxa* (Ta-an) displayed low RFC (2.1%) and FL% (53.1%), indicating a decline in its traditional usage for stomach ailments, possibly due to the availability of preferred alternatives. Plants with high RFC and FL% values, such as *K. senegalensis, Pseudocedrela kotschyi*, *Mitragyna inermis*, and *Combretum molle*, are widely recognized and utilised explicitly for treating stomach-related diseases. Conversely, plants with low RFC but high FL%, such as *Detarium senegalense* and *Cochlospermum planchoni*, may offer untapped potential for specialized treatments. Details of the results are shown in  Table 4 below.

**Table 4.** Information on plants used to treat stomach-related diseases.

Voucher number	Scientific name	Family	Local name	Frequency of citation	RFC	FL	Other ailments	Side effect
KNUST/HM1/2023/R006	*Securidaca longepedunculata Fresen.*	Polygalaceae	Pelig	23	2.9	11.7	Inflammation	Yes, causes abortion
KNUST/HM1/2025/SB013	*Khaya senegalensis* (Desr.) A. Juss.	Meliaceae	Kok	149	18.9	63.1	Anthrax, boil, waist pains, aphrodisiac, wound healing	No
KNUST/HM1/2025/L024	*Mitragyna inermis* (Willd.) Kuntze	Rubiaceae	Yeblig	75	9.5	93.8	Swollen legs, waist pains, boosts appetite, aids delivery, additive to fracture treatment	No
KNUST/HM1/2025/L025	*Combretum molle* R. Br. ex G. Don	Combretaceae	Gberig	58	7.4	98.3	Appetite booster, toothache, waist pains	No
KNUST/HM1/2025/L027	*Nauclea orientalis* (L.) L.	Rubiaceae	Gu-un	36	4.6	83.7	Adjuvant to snake-bite treatment	No
KNUST/HM1/2025/SB012	*Pseudocedrela kotschyi* (Schweinf.) Harms	Meliaceae	Shiarig/Shiarit	131	16.6	55.7	Anthrax, inflammation, boil, antimicrobial, snake-bite additive, headache, wound healing	No
KNUST/HM1/2020/SB010	*Anogeissus leiocarpus* (DC.) Guill. & Perr.	Combretaceae	Cii	49	6.2	94.2	Cough	No
KNUST/HM1/2017/SB011	*Terminalia macroptera* Guill. Perr.	Combretaceae	Podaa	39	4.9	92.9	Toothache	No
KNUST/HM1/2018/L058	*Vitex doniana* Sweet	Lamiaceae	Aa-rig	23	2.9	92.0	Prevent miscarriage	No
KNUST/HM1/2017/L005	*Lannea microcarpa* Engl. & K. Krause	Anacardiaceae	Sisablik	9	1.1	81.8	Blood tonic, burns	No
KNUST/HM1/2025/L026	*Adesmia incana* var. grisea Hook. f.	Fabaceae	Kbantiug	51	6.5	92.7		No
KNUST/HM1/2017/L001	*Cassia sieberiana* DC.	Fabaceae	Kolkbawure	42	5.3	91.3		No
KNUST/HM1/2025/SB017	*Ficus gnaphalocarpa* (Miq.) Steud. ex A. Rich	Moraceae	Kikan	4	0.5	80.0	Cough	No
KNUST/HM1/2025/L029	*Detarium senegalense* J.F. Gmel.	Fabaceae	Kbarig	12	1.5	100.0		No
KNUST/HM1/2019/R002	*Sclerocarya birrea* (A. Rich.) Hochst.	Anacardiaceae	Nanobig	8	1.0	100.0	Cancer of breast and testes	No
KNUST/HM1/2018/L052	*Vitellaria paradoxa* C.F. Gaertn.	Sapotaceae	Ta-an	17	2.2	53.1		No
KNUST/HM1/2023/F001	*Parkia biglobosa* (Jacq.) R. Br. ex Don	Fabaceae	Duo	11	1.4	73.3		No
KNUST/HM1/2025/SB001	*Diospyros mespiliformis* Hochst. ex A. DC.	Ebenaceae	Gar	6	0.8	100.0	Rib pains	No
KNUST/HM1/2025/L030	*Cochlospermum planchonii* Hook.	Bixaceae/Cochlospermaceae	Bugla	12	1.5	100.0		No
KNUST/HM1/2025/L028	*Ziziphus spina-christi * (L.) Desf.	Rhamnaceae	Lien	11	1.4	64.7		No
KNUST/HM1/2025/L005	*Azadirachta indica* A. Juss.	Meliaceae	Nim tree	6	0.8	4.4		No
KNUST/HM1/2025/L006	*Vernonia amygdalina* Delile	Asteraceae	Suwaka	1	0.1	33.3		No
KNUST/HM1/2019/R014	*Borassus aethiopum* Mart.	Arecaceae	Karikon	1	0.1	100.0		No
KNUST/HM1/2019/R006	*Cantinoa americana* (Aubl.) Harley & J.F.B. Pastore	Lamiaceae	Dunkwaq	1	0.1	25.0		No
KNUST/HM1/2019/R008	*Acacia sieberiana* DC. var.	Fabaceae (Mimosoideae)	GUOpelug	3	0.4	100.0		No
KNUST/HM1/2019/R015	*Psidium guajava* L.	Myrtaceae	Guava	1	0.1	100.0		No
KNUST/HM1/2019/R010	*Dialium indum* L.	Fabaceae	Nolig	2	0.3	100.0		No
KNUST/HM1/2019/R011	*Waltheria indica* L.	Malvaceae	Dabirig	2	0.3	100.0		No
KNUST/HM1/2016/SB013	*Piliostigma reticulatum* (DC.) Hochst.	Fabaceae	Ba-an	5	0.6	26.3		No

***Plants used in the treatment of skin-related diseases/dermatological disorders.*** Securidaca longepedunculata (locally known as Pelig) was the most frequently cited plant for dermatological treatments, with 127 citations (35.8% RFC, 67.6% FL%). To treat boils or inflammation, the powdered plant material is mixed with water to form a paste, which is applied directly to the affected area. Beyond skin-related ailments, Securidaca longepedunculata is a highly versatile medicinal plant used for various health conditions. For headaches, a small amount of water is added to the powdered plant to create a paste, which is applied to the forehead. A decoction of the plant is consumed to manage diarrhoea and ulcers, while anthrax is treated by adding the plant to suspected infected meat or carcasses before boiling.

The root bark is also placed in homes or farmhouses as a natural repellent against snakes and scorpions. The plant also aids digestion, with a decoction taken to alleviate indigestion. Also, a warm root infusion is consumed with flour to reduce blood clotting after delivery. The high Relative Frequency of Citation (RFC) and significantly elevated Fidelity Level (FL%) values highlight the plant’s widespread recognition and trust within the community, particularly for dermatological applications. Our findings align with a study by Blench and colleagues, who documented *Securidaca longepedunculata* as an essential medicinal plant used in northern Ghana for disease treatment. ^50^ However, the use of *Securidaca longepedunculata* should be cautiously approached due to its potential side effects, particularly its abortifacient properties. This highlights the need for careful dosage regulation and awareness of its contraindications. Also, the second most frequently cited plant for treating inflammation and boils was *Pseudocedrela kotschyi* (locally known as *Shiarig*), with 100 citations (28.2% RFC, 42.6% FL%). To treat these conditions, the root or stem bark is crushed and mixed with a small amount of water to form a paste, which is then applied to the affected area. Beyond dermatological applications, *Pseudocedrela kotschyi* is also used to treat headaches, diarrhoea, cough, anthrax, toothache, and stomach pains. Our findings align with previous research, which has extensively documented the medicinal uses of *Pseudocedrela kotschyi* in African traditional medicine. Studies have highlighted its broad pharmacological properties, particularly its effectiveness as an analgesic, antimicrobial, antimalarial, anthelmintic, and antidiarrheal agent. ^59^^,^^60^ The high RFC reflects the strong cultural significance of *Pseudocedrela kotschyi*, while the moderate FL% suggests some variability in its perceived effectiveness or specific applications for skin conditions. Additionally, *K. senegalensis* (locally known as *Kok*) ranked third, with 79 citations (22.3% RFC, 33.6% FL%). According to informants, the powdered form of *K. senegalensis* is applied directly to the affected area to treat boils and inflammations. Beyond dermatological uses, it is also traditionally used for managing headaches, diarrhoea, and stomach pains. However, ethnobotanical studies from other regions have documented a broader spectrum of medicinal applications for *K. senegalensis*, particularly its use in treating malaria, stomach disorders, and other ailments. ^63^ Its RFC value indicates moderate cultural importance, while the FL% suggests a broader yet less specific application for skin-related diseases. However, certain plants, such as *Zingiber officinale*, *Allium sativum*, *Mitragyna inermis*, and *Strophanthus hispidus*, achieved perfect FL% values (100%), indicating unanimous agreement among informants regarding their dermatological applications. The consistent use of these plants for treating inflammation and other ailments aligns with reports from other countries, ^50^^,^^63^ further reinforcing their recognized therapeutic potential across different traditional medicine systems. However, their low RFC values suggest that while these plants are highly specific in their applications, they are not widely recognized or commonly utilized within the community. This observation underscores the importance of combining quantitative indices with qualitative insights to gain a more comprehensive understanding of medicinal plants’ cultural and therapeutic significance, aligning with other researchers on the value of integrated approach in ethnobotanical research. ^12^^,^^64^^,^^65^ The observed variations in plant use and consensus may be influenced by regional availability, traditional healing practices, and the diverse range of ailments these plants treat. These factors contribute to differences in the recognition and frequency of medicinal plants use across communities.  Table 5 below shows the %RFC, FC, and %FC of plants used to treat skin-related diseases. Additionally, it outlines other medicinal applications of these plants and any reported side effects.

**Table 5.** Information of plants used to treat skin-related diseases.

Voucher number	Scientific name	Family	Local name	Frequency of citation	RFC	FL (%)	Other treatment	Side effect
KNUST/HM1/2023/R006	*Securidaca longipedunculata* Fresen.	Polygalaceae	Pelig	132	37.0	67.3	Headache, diarrhoea, snake & scorpion repellent/bite, all kinds of diseases, removing blood clot, anthrax, ulcer, indigestion	Yes, causes abortion
KNUST/HM1/2025/L027	*Nauclea orientalis* (L.) L.	Rubiaceae	Gu-un	7	2.0	16.3	Stomach ache	No
KNUST/HM1/2025/SB013	*Khaya senegalensis* (Desr.) A. Juss.	Meliaceae	Kok	79	22.1	33.5	Headache, diarrhoea, stomach pains	No
KNUST/HM1/2025/SB012	*Pseudocedrela kotschyi* (Schweinf.) Harms.	Meliaceae	Shiarig	100	28.0	42.6	Snake bite, stomach ache, anthrax, indigestion, ulcer treatment, strengthen teeth, cough	No
KNUST/HM1/2017/SB011	*Terminalia macroptera* Guill. Perr.	Combretaceae	Podaa	3	0.8	7.1		No
KNUST/HM1/2019/R016	*Strophanthus hispidus* DC.	Apocynaceae	Yarbik	1	0.3	100.0	Ringworm, arrow poison, eye treatment	No
KNUST/HM1/2019/R017	*Zingiber officinale* Roscoe	Zingiberaceae	Kakadru/Ginga	1	0.3	100.0		No
KNUST/HM1/2019/R012	*Allium sativum* L.	Amaryllidaceae	Garlic	2	0.6	100.0	Ear problem	No
KNUST/HM1/2025/L025	*Combretum molle* R.Br. ex G.Don	Combretaceae	Gberig	1	0.3	1.7		No
KNUST/HM1/2023/F001	*Parkia biglobosa* (Jacq.) R. Br. ex G. Don	Fabaceae	Duo	4	1.1	26.7		No
KNUST/HM1/2016/SB013	*Piliostigma reticulatum* (DC.) Hochst.	Fabaceae	Ba-an	1	0.3	5.3		No
KNUST/HM1/2025/L028	*Ziziphus spina-christi * (L.) Desf.	Rhamnaceae	Lien	5	1.4	29.4	Additive to snake-bite treatment	No
KNUST/HM1/2025/L024	*Mitragyna inermis* (Willd.) Kuntze	Rubiaceae	Yeblig	4	1.1	5.0		No
KNUST/HM1/2019/R018	*Annona senegalensis* Pers.	Annonaceae	Baburig	1	0.3	100.0		No
KNUST/HM1/2019/R007	*Adansonia digitata* L.	Malvaceae	Tuo	1	0.3	33.3		No
KNUST/HM1/2020/SB010	*Anogeissus leiocarpus* (DC.) Guill. & Perr.	Combretaceae	Cii	2	0.6	3.8		No
KNUST/HM1/2019/R019	*Phaseolus vulgaris* L.	Fabaceae	Beans/tiya	1	0.3	100.0		No
KNUST/HM1/2019/R020	*Abelmoschus esculentus* (L.) Moench	Malvaceae	Okro/Ma-a-na	1	0.3	100.0		No
KNUST/HM1/2018/L052	*Vitellaria paradoxa* C.F. Gaertn.	Sapotaceae	Ta-an	4	1.1	12.5	Diarrhoea	No
KNUST/HM1/2018/L006	*Mangifera indica* L.	Anacardiaceae	Mongo tii	1	0.3	1.2		No
KNUST/HM1/2017/L001	*Cassia sieberiana* DC.	Fabaceae	Kolkbawure	2	0.6	4.3		No
KNUST/HM1/2025/L005	*Azadirachta indica* A. Juss.	Meliaceae	Nim ti	1	0.3	0.7		No
KNUST/HM1/2019/R021	*Paullinia pinnata* L.	Sapindaceae	Gerisablig	1	0.3	100.0		No
KNUST/HM1/2019/R022	*Vossia cuspidata* Griff.	Poaceae	Kontinchioh	1	0.3	100.0		No
KNUST/HM1/2019/R003	*Ficus gnaphalocarpa* (Miq.) Steud. ex A. Rich.	Moraceae	Kikan	1	0.3	20.0	Diarrhoea, enhance breast-milk flow	No
KNUST/HM1/2019/R013	*Portulaca oleracea* L.	Portulacaceae	Baatob-zariba	1	0.3	100.0		No

***Plants for malaria treatment.*** This study underscores the ethnobotanical richness and cultural significance of medicinal plants utilized for malaria treatment in traditional medicine. By analyzing the Relative Frequency of Citation (RFC) and Fidelity Level (FL%), the study identifies key species with widespread use and specificity, providing valuable insights for future pharmacological exploration. *Azadirachta indica* (locally called Nim tii), with 130 citations, the highest RFC (38.0%), and a high FL% (94.2%), emerged as the most frequently cited and culturally significant plant for malaria treatment. Its high FL% demonstrates strong agreement among traditional practitioners, emphasizing its specificity and reliability. To treat malaria, the leaves are combined with the leaves of *Mangifera indica, Moringa oleifera*, and *Carica papaya* and boiled. The boiled water is used for a steam bath for about 3 days to cure malaria. Previous studies have similarly highlighted the antimalarial properties of *A. indica* due to its bioactive compounds, including limonoids and azadirachtin, which inhibit the malaria parasite’s life cycle. ^36^^,^^37^^,^^66^^,^^67^ Also, *M. indica* (locally called Mongo tii) ranks second, with 83 citations, an RFC of 24.1%, and an FL% of 98.8%. Its high RFC reflects its widespread recognition, while the near-perfect FL% highlights its strong specificity for malaria treatment. *M. indica* contains mangiferin, a compound with documented antimalarial and immunomodulatory effects. ^36^^,^^46^ Although *M. oleifera* (43 citations, 12.8% RFC) has a relatively lower RFC compared to *A. indica* and *M. indica, * it demonstrates a perfect FL% (100%), underscoring its exceptional specificity for malaria treatment. This aligns with reports of its anti-plasmodial activity, likely due to its rich phytochemical profile, including flavonoids and alkaloids. ^68^^,^^69^ *C. papaya* (locally Pawpaw) also shows significant potential, with 59 citations, an RFC of 17.1%, and an FL% of 95.2%. The high FL% reflects consistent use and agreement among practitioners, further validating its medicinal relevance for malaria. An ethnobotanical study in Nigeria revealed that the plant is used to treat malaria, stomach ulcer, and deworming, ^70^ thus agreeing with our findings. The plant contains bioactive compounds, such as papain and alkaloids, which have been associated with antimalarial activity. ^68^^,^^69^ Several other species, including *Ananas comosus* (Pineapple), *Mimosa pudica* (Wabur-kasakur), used in China to treat bruises, ulcers, abscesses, boils, haemorrhoids and fractures, ^71^^,^^72^ and *Cantinoa americana* (Dunpbawq), achieved perfect FL% (100%) but had low RFC values (0.9%). This suggests these plants are highly specific for malaria treatment but are less well-known or regionally restricted in their use. These plants warrant further exploration for their therapeutic potential despite their limited popularity. Conversely, plants such as *V. paradoxa* (0.3% RFC, 3.1% FL%) and *K. senegalensis* (1.7% RFC, 2.6% FL%) exhibited low scores for both RFC and FL%. This may indicate limited regional availability, broader medicinal applications, or lesser recognition as primary malaria treatments.  Table 6 below shows %RFC, FC, %Fl of plants used for treating malaria and other diseases plants that can treat and side effects, if any.

**Table 6.** Information about plants used for the treatment of malaria.

Voucher number	Scientific name	Family	Local name	Frequency of citation	RFC	FL (%)	Other treatment	Side effect
KNUST/HM1/2025/L005	*Azadirachta indica A. Juss.*	Meliaceae	Nim tii	130	38.8	94.9		No
KNUST/HM1/2023/F009	*Carica papaya* L.	Caricaceae	Pawpaw	58	17.3	95.1		No
KNUST/HM1/2018/L006	*Mangifera indica* L.	Anacardiaceae	Mongo	83	24.8	98.8		No
KNUST/HM1/2018/L052	*Vitellaria paradoxa C.F. Gaertn.*	Sapotaceae	Ta-an	1	0.3	3.1		No
KNUST/HM1/2019/R023	*Ananas comosus* (L.) Merr.	Bromeliaceae	Pineapple	1	0.3	100.0		No
KNUST/HM1/2019/R009	*Vernonia amygdalina* Delile	Asteraceae	Suwaka	2	0.6	66.7		No
KNUST/HM1/2017/L005	*Lannea microcarpa Engl. & K. Krause*	Anacardiaceae	Sisablik	1	0.3	9.1		No
KNUST/HM1/2024/L013	*Moringa oleifera* Lam.	Moringaceae	Moringa	43	12.8	100.0		No
KNUST/HM1/2018/L058	*Vitex doniana* Sweet	Lamiaceae	Aa-rig	2	0.6	8.0		No
KNUST/HM1/2017/L001	*Cassia sieberiana* DC.	Fabaceae	Kolbawure	1	0.3	2.2		No
KNUST/HM1/2017/L009	*Jatropha gossypiifolia* L.	Euphorbiaceae	Black flowers/flower sabil	1	0.3	100.0		No
KNUST/HM1/2020/SB010	*Anogeissus leiocarpus* (DC.) Guill. & Perr.	Combretaceae	Cii	1	0.3	1.9		No
KNUST/HM1/2025/SB013	*Khaya senegalensis* (Desr.) A. Juss.	Meliaceae	Kok	6	1.8	2.5		No
KNUST/HM1/2019/R006	*Cantinoa americana* (Aubl.) Harley & J.F.B. Pastore	Lamiaceae	Dunkwaq	3	0.9	75.0		No
KNUST/HM1/2019/R024	*Mimosa pudica* L.	Fabaceae	Wabur-kasakur	1	0.3	100.0		No

### Use Reports and Use Value (UV) analysis

The Use Reports (UR) and Use Value (UV) analysis offer critical insights into the cultural significance and therapeutic applications of medicinal plants within the study area. These metrics reflect the frequency of plant use and indicate their perceived importance in traditional medicine. The findings highlight a spectrum of usage, ranging from highly utilized species to those with specialized or declining roles, providing a comprehensive view of the community’s ethnobotanical practices. Figure 6 shows the results of the analysis. However, plant with use value less than 0.05 not shown in the graph because of space.

**Figure 5.**
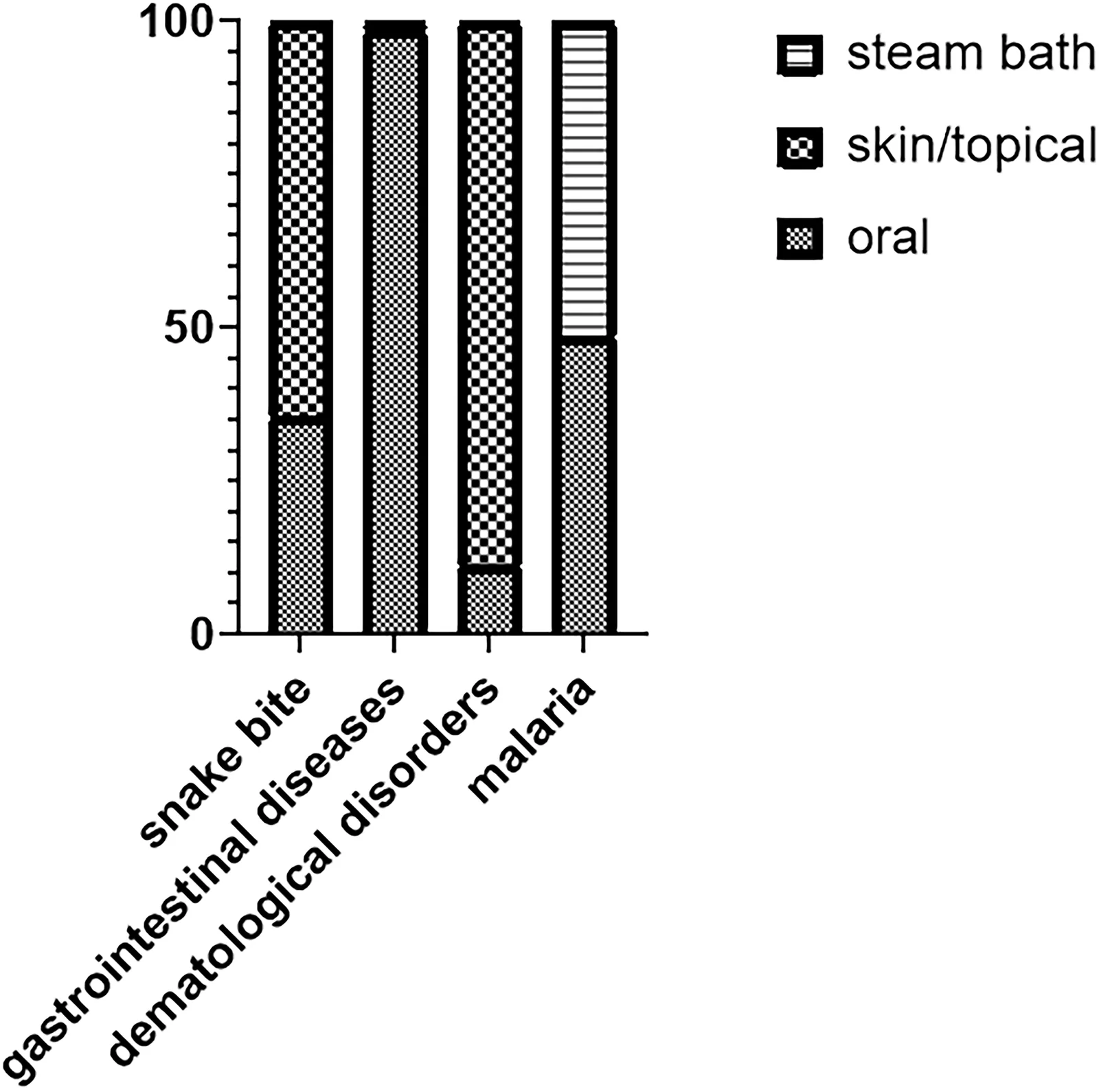
Percentage citation of mode of administration of plant for each ailment category.

*Khaya senegalensis* and *Pseudocedrela kotschyi* stand out as the most prominent species, with the highest UR (235) and UR (235) respectively and UV (1.07) and UV (1.07) respectively. Their high UV underscores strong informant consensus and widespread use, suggesting their central role in the community’s pharmacopoeia. These plants’ extensive application for common and severe ailments likely reflects their bioactive properties and therapeutic efficacy, aligning with reports of their use in treating various kinds of diseases, including diarrhea, dysentery, wound infections, diabetes, hypertension, jaundice, and malarial, dermatitis, scorpion bites, mental illness, pain, and inflammation. ^57^^–^^60^

Other species, including *Securidaca longepedunculata* (UR 188, UV 0.86), and *Azadirachta indica* (UR 139, UV 0.63), demonstrate substantial use and cultural value. These plants are integral to the community’s healthcare system and are widely employed for a range of ailments, particularly malaria and other febrile conditions. The consistent use of these species may be attributed to their potent phytochemicals, such as the saponins and alkaloids in *S. longepedunculata* and *A. indica.* ^49^^,^^73^

Also, plants such as *Mangifera indica* (UR 84, UV 0.38) and *Mitragyna inermis* (UR 82, UV 0.37) occupy a middle tier of importance. These species are likely used for specific conditions or as complementary remedies. Their moderate UV values suggest variability in their perceived efficacy, availability, or ease of preparation. For instance, *M. indica* is known for its broad-spectrum medicinal applications but may not be as culturally embedded as species like *Pseudocedrela kotschyi* or *A. indica.*

However, a significant number of plants exhibit low UR and UV values, such as *Psidium guajava*, *Phaseolus vulgaris*, *Abelmoschus esculentus*, and others with UVs near 0. These species may have niche applications, treating rare or culturally specific conditions, or their use may be declining due to shifts in traditional practices or availability. Despite their low UV, these plants should not be overlooked, as they may contain unique bioactive compounds with pharmacological potential. For instance, *P. guajava* is recognized for its antibacterial and antidiarrheal properties despite limited UR in this study. ^36^^,^^66^

Beyond the primary disease categories, some plants serve unique roles in traditional healthcare. For example, *Sterculia setigera* (locally Pupung) is used for cough treatment, *Kapok bark* (locally Gungun) is employed for respiratory ailments, and *Ocimum canum Sims seeds* (locally Hnuhnu) are used for eye cleaning. These uses reflect the diverse and multifaceted applications of medicinal plants within the community and underscore the importance of documenting such knowledge for cultural preservation and future research.

## Conclusion

This study documented the ethnomedicinal plants used by the Talensi District’s people to treat snakebites, gastrointestinal diseases, dermatological diseases, and malaria. It also examined the plant parts used, modes of preparation, and administration methods for each disease category. The Informant Consensus Factor (ICF) analysis demonstrated a strong agreement among informants regarding the medicinal plants used for each ailment, highlighting the reliability of traditional knowledge.

While plant utilization varied depending on the disease category, certain patterns emerged. Leaves were predominantly used for malaria treatment, whereas roots and stems were the preferred plant parts for other ailments. The Relative Frequency of Citation (RFC) and Fidelity Index (FI) analyses revealed that specific plants were more frequently cited for treating particular diseases, underscoring their significance in traditional healing practices. The overall Use Value (UV) analysis identified *Pseudocedrela kotschyi*, *Securidaca longepedunculata*, *K. senegalensis*, and *A. indica* as the most widely used medicinal plants.

These findings highlight the wealth of ethnomedicinal knowledge among the Talensi people and provide a foundation for future pharmacological investigations. The study underscores the importance of preserving and documenting traditional plant-based medicine, ensuring that this valuable indigenous knowledge is transmitted to future generations and potentially contributing to drug discovery and public health interventions.

### Declaration statements

#### Consent to participate

A written informed consent was obtained from all individual participants included in the study.

#### Ethics statement

The questionnaire and methodology for this study was prepared in accordance with the Kwame Nkrumah University of Science and Technology Research Ethics policy and approved by the Committee on Human Research, Publication and Ethics. Ethics approval number: CHRPE/AP/033/22. Approval date: February 2, 2022. The study did not involve clinical trial.

### Data availability statement

All data and materials supporting the results and analyses in this paper is published in Harvard Dataverse -view at https://doi.org/10.7910/DVN/QXCKJ0. ^74^

Data are available under the terms of the Creative Commons 1.0 Universal License (CC0 1.0).
