# Ethnobotanical Study of Indigenous Knowledge on Medicinal Plants Used in the Talensi District of the Upper East Region of Ghana

**DOI:** 10.12688/openresafrica.15807.4

**Published:** 2026-05-22

**Authors:** Emmanuel Azanlerigo, Philip T. Thompson, Cephas Kanati, Mercy Badu

**Affiliations:** 1 Chemistry, Kwame Nkrumah University of Science and Technology Department of Chemistry, Kumasi, Ashanti Region, Ghana

**Keywords:** ethnobotany, medicinal plants, dermatological, gastrointestinal, Talensi District, malaria, snake bite, skin

## Abstract

**Background:**

The Talensi and Nabdam districts of northern Ghana maintain a rich tradition of plant-based medicine integrated with ritual healing. However, this knowledge has not previously been systematically and quantitatively documented. This study aimed to record medicinal plant use and analyse patterns of therapeutic importance across major disease categories.

**Methods:**

Between February 2023 and August 2024, semi-structured interviews were conducted with 219 informants. Relative Frequency of Citation (RFC), Use Value (UV), Fidelity Level (FL), Informant Consensus Factor (ICF), and Plant Part Value (PPV) were calculated. Inferential analyses included Spearman’s rank correlation and Kruskal–Wallis tests with Dunn’s post-hoc comparisons. A plant–disease interaction network was visualised using a chord diagram. Analyses were performed in R and GraphPad Prism.

**Results:**

Forty-six medicinal plant species belonging to 46 genera and 27 families were recorded for the treatment of gastrointestinal disorders, dermatological conditions, malaria, and snakebite. Roots dominated treatments for snakebite, skin and gastrointestinal disorders, while leaves were primarily used for malaria. Informant consensus was high for gastrointestinal disorders (ICF = 0.96), malaria (0.96), and skin diseases (0.93). RFC and UV were strongly correlated (ρ = 0.85, p < 0.001). Network analysis identified *Khaya senegalensis*, *Pseudocedrela kotschyi*, *Securidaca longepedunculata* and *Azadirachta indica* as the most culturally and therapeutically important species.

**Conclusion:**

Talensi ethnomedicinal knowledge is structured around a small set of highly versatile plants, providing strong candidates for pharmacological and conservation prioritisation.

**Abbreviations**
ICF: Informant Consensus Factor
RFC: Relative Frequency of Citation
FL: Fidelity Level
UV: Use Value
PPV: Plant Part Value
FC: Frequency of Citation
NA: not specified

## Introduction

Medicinal plants remain a cornerstone of primary healthcare for many rural and Indigenous communities across sub-Saharan Africa. In Ghana’s savanna belt, particularly within the Guinea and Sudan savanna zones, plant-based therapies are widely used to manage infectious, inflammatory, and parasitic diseases due to their accessibility, cultural acceptance, and affordability. ^1^ ^,^ ^2^ The Upper East Region, characterised by a short unimodal rainy season (600–1000 mm) and a mosaic of woodland and grassland vegetation, supports a rich diversity of medicinal flora adapted to these semi-arid conditions. ^3^ ^–^ ^5^

The Talensi and Nabdam districts form part of this ecological and cultural landscape and possess a long-standing tradition of herbal medicine embedded within clan-based and ritual healing systems. Historically, specialised knowledge of medicinal plants has been transmitted orally within families and among traditional healers. However, this knowledge system is increasingly threatened by sociocultural change. During field interviews, practitioners repeatedly reported that religious conversion, formal education, and modernisation have reduced intergenerational knowledge transfer, as younger people increasingly perceive traditional medicine as incompatible with Christian or Islamic beliefs. Similar trends have been reported elsewhere in West Africa, where religious transformation and environmental change have contributed to the erosion of ethnomedicinal knowledge. ^6^ ^–^ ^8^

Despite the cultural and therapeutic importance of herbal medicine in this region, no systematic quantitative ethnobotanical study has previously been conducted in the Talensi or Nabdam districts. Existing studies ^9^ ^,^ ^10^ in northern Ghana have documented medicinal plant use in neighbouring districts, but none has evaluated the internal structure, consensus, and cultural importance of species within the Talensi–Nabdam ethnomedical system. Consequently, the degree to which medicinal knowledge is shared, specialised, or consolidated across disease categories remains unknown.

In this context, the present study was designed to document medicinal plants used by the Talensi people and to quantitatively analyse patterns of ethnomedicinal knowledge using established ethnobotanical indices and inferential statistics. The study focuses on four major disease categories—snakebite, gastrointestinal disorders, dermatological diseases, and malaria—which represent leading health burdens in the region. ^11^ ^–^ ^13^ By integrating indices such as RFC, UV, FL, and ICF with correlation analysis, non-parametric tests, and network visualisation, this study provides the first system-level assessment of traditional medicinal knowledge in the Talensi–Nabdam area, contributing to cultural preservation, conservation planning, and pharmacological prioritisation. ^14^

## Materials and methods

### Location, topography, vegetation cover and climate

The study was conducted in the Talensi District of the Upper East Region, Ghana, from March to September 2024. The district lies within the boundaries of longitudes 0°31′ and 1°05′ west and latitudes 10°35′ and 10°60′ north. The Talensi District, formally Talensi-Nabdam District, was established in 2012, covers an area of 845.3 km ^2^ with a population of 87,021 as of the 2021 census, 102.9/km ^2^ Population Density and 0.65% Annual Population Change from 2010 to 2021. ^3^ ^–^ ^5^ Most of the district’s inhabitants (about 90%) are peasant farmers. The topography of the district is generally flat, with relatively undulating lowlands and gentle slopes ranging between 1% and 5%. Elevations range between 100 m and 200 m, with some isolated rock outcrops and uplands mainly around the district capital Tongo and Yinduri reaching 400 m high. Several rivers and tributaries drain the district during the rainy season. In the dry season, most tributaries dry out, leaving only portions of the Red and White Volta Rivers flowing through the district. The area is almost always relatively warm, with temperatures reaching 45 °C in March and April and recording a minimum of 12 °C in December. ^3^ ^–^ ^5^ The prolonged dry season is accompanied by very low relative humidity, which reaches 10% during harmattan (the dry and hazy northeast trade wind) in December and January. With the onset of the rains, it rises gradually to a maximum of 65%. Figure 1 below shows the sketch of the map of the study area.

**Figure 1.**
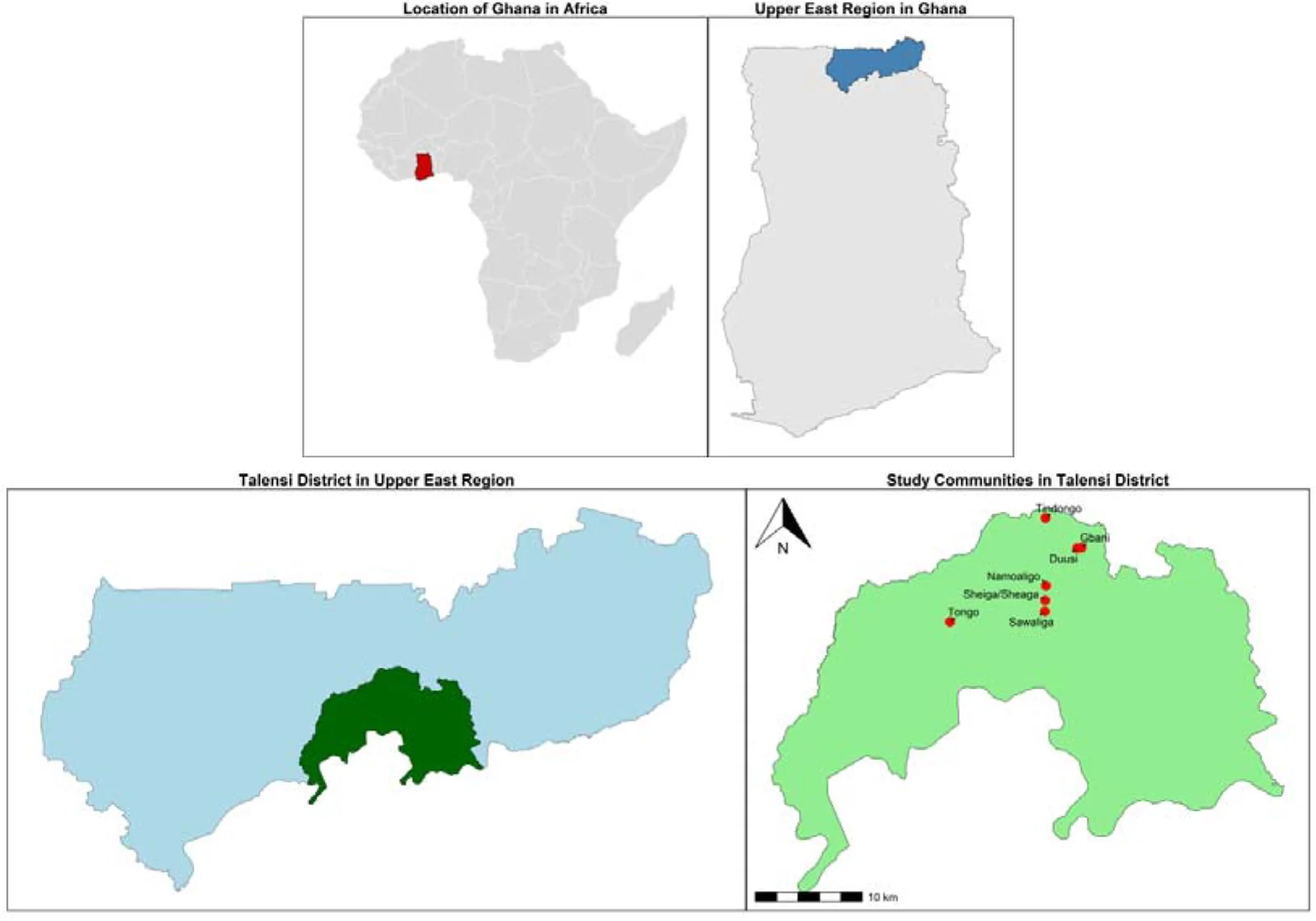
A map of the study area.

### Data collection and sampling strategy

Data were collected through semi-structured interviews with 219 respondents aged 30 years and above across selected communities in the Talensi District. Interviews were conducted in the local Taleni/Frafra dialect to ensure cultural appropriateness and clarity, while responses were recorded in English using a semi-structured questionnaire designed in Google Forms. Vernacular plant names were documented as provided by informants and later matched with scientific names during field collection and taxonomic verification.

A mixed non-probabilistic sampling strategy was employed, combining household-based purposive sampling with targeted snowball sampling. Initially, respondents were selected through house-to-house visits, focusing on individuals aged ≥30 years, as this group was considered more likely to possess accumulated indigenous knowledge through long-term experience. During these interviews, respondents were also asked to identify individuals recognized within the community as possessing advanced ethnomedicinal knowledge, such as traditional healers. These referrals formed the basis of a targeted snowball approach used exclusively to recruit key informants.

This combined strategy allowed for the inclusion of both general household knowledge and specialist expertise, thereby improving the representativeness and reliability of the ethnobotanical dataset while minimizing bias associated with sampling only traditional healers.

Prior to each interview, the objectives of the study were clearly explained to participants, and written informed consent was obtained; thumbprints were accepted from participants unable to sign. The study adhered to the International Society of Ethnobiology Code of Ethics, ^15^ ensuring voluntary participation, confidentiality, and respectful documentation of indigenous knowledge. The questionnaire comprised five sections covering demographic characteristics and ethnomedicinal plant use for snakebite envenomation, gastrointestinal diseases, dermatological conditions, and malaria. For each disease category, informants provided information on plant species used, plant parts utilized, methods of preparation, routes of administration, additional therapeutic uses, and perceived side effects. Ethical clearance was subsequently granted by the Committee on Human Research, Publications and Ethics (CHRPE), Kwame Nkrumah University of Science and Technology (KNUST), Ghana, on 9th July 2025 (Approval No.: CHRPE:/AP/576/25).

### Sample size justification

The study involved 219 respondents. Although Cochran’s formula was consulted as a reference for population-based surveys (yielding a theoretical sample size of 382 based on a population of 87,021). However, ethnobotanical research prioritizes knowledge saturation rather than probabilistic generalization.

Accordingly, the final sample size was determined based on field feasibility, informant availability, and informational saturation, where successive interviews yielded few additional plant species or use reports. This sample size was considered sufficient to capture the internal diversity and consistency of medicinal plant knowledge in the study area. Comparable sample sizes have been widely reported in similar ethnobotanical studies employing non-probabilistic designs. ^16^ ^,^ ^17^

### Plant identification

Plant identification was conducted in two stages. First, reported plants were located and identified in the field with the assistance of experienced local guides and traditional healers. Following interviews, field visits were conducted to collect voucher specimens from nearby forests and fallow lands.

All specimens were subsequently authenticated at the Department of Herbal Medicine, Faculty of Pharmacy and Pharmaceutical Sciences, Kwame Nkrumah University of Science and Technology (KNUST). Taxonomic verification was carried out by qualified botanists through comparison with reference herbarium collections and standard botanical literature. Voucher specimens were deposited at the KNUST Herbarium, and corresponding voucher numbers are provided in Tables 4– 6.

### Data analysis

Data were cleaned and organised using Microsoft Excel. Quantitative ethnobotanical indices and statistical analyses were performed using GraphPad Prism (version 8.0.1) and R (version 4.5.2). Descriptive ethnobotanical indices included Relative Frequency of Citation (RFC, expressed as percentage), Use Value (UV), Fidelity Level (FL), Informant Consensus Factor (ICF), Plant Part Value (PPV), and percentage distributions of modes of preparation and administration.

Inferential analyses were conducted to evaluate patterns and relationships within the dataset. Spearman’s rank correlation was used to assess the association between RFC and UV, while differences in RFC among disease categories were tested using the Kruskal–Wallis test followed by Dunn’s post-hoc test with Bonferroni correction. To complement the statistical analyses and explore the structure of ethnomedicinal knowledge, a bipartite plant–disease network was constructed based on RFC values and visualised using a chord diagram generated in R (circlize package). This network-based visualisation was used to identify highly connected species, disease-specific specialisation, and overall patterns of ethnobotanical knowledge distribution.

### Quantitative indices

Quantitative ethnobotanical indices were calculated to assess the cultural importance, specificity, and consensus of medicinal plant use. These included Informant Consensus Factor (ICF), Relative Frequency of Citation (RFC), Fidelity Level (FL), Plant Part Value (PPV), and Use Value (UV). These indices are widely applied in quantitative ethnobotany to evaluate informant agreement, cultural salience of species, disease-specific preferences, and overall ethnomedicinal importance. ^14^ ^,^ ^18^ ^,^ ^19^

Higher values of RFC, UV, FL, and ICF were interpreted as indicators of greater cultural relevance, higher use intensity, stronger specificity of use, and increased consensus among informants, respectively. These indices were selected based on their established utility in identifying culturally significant species and prioritizing plants for conservation and pharmacological investigation. ^14^ ^,^ ^18^ ^,^ ^19^

### Justification for disease categories and application of indices

The ethnobotanical survey focused on four disease categories: gastrointestinal disorders, dermatological conditions, snakebite, and malaria. These categories were selected based on field observations, informant consensus, and their high prevalence within the study area. Additionally, they correspond to globally recognized public health challenges, including malaria ^20^ ^,^ ^21^ and snakebite envenomation, ^22^ as well as infectious diseases associated with antimicrobial resistance. ^23^ ^,^ ^24^

Focusing on these categories facilitated accurate recall by informants and allowed disease-specific assessment of plant importance. All indices, except UV, were calculated separately for each disease category, while UV was computed across all categories to reflect overall ethnomedicinal importance.

### Calculation of quantitative indices

***Plant Part Value.*** The plant part value (PPV) presents the proportion of different plant parts (stem, leaves, root, fruit, bark, and flower) used as medicinal resources. The PPV is calculated according to Refs. 25 and 26 as follows:

PPV(%)=[ΣRU(plantpart)/ΣRU]*100 (1)

∑RU (plant part) and ∑RU represent the sum of the cited plant parts and the number of cited uses for a given plant, respectively. The same approach was applied to analyze modes of preparation and administration. Results are presented in charts for clarity.

***Informant Consensus Factor.*** Informant Consensus Factor (ICF) was used to evaluate the degree of agreement among informants regarding plant use for each disease category ^26^ ^–^ ^28^ and was calculated:

$$\text{ICF}=\text{Σ}({\text{N}}_{\text{ur}}-{\text{N}}_{\text{t}})\text{/Σ}({\text{N}}_{\text{ur}}-1)$$ (2)

where Σ N _ur_ is the total number of use reports for a given disease category and Σ N _t_ is the number of plant species used for that category. ICF values range from 0 to 1, with higher values indicating greater consensus among informants.

***Relative Frequency of Citation.*** Relative frequency of citation (RFC), expressed percentages, was calculated to assess the popularity of medicinal plant species in each disease category using the formula:

RFC=(ΣFC/ΣN)*100 (3)

where FC is the number of informants who cited a given species and N is the total number of informants interviewed. Higher RFC values indicate widely recognized and frequently cited species. ^26^ ^–^ ^28^

***Fidelity Level.*** The percentage of the most popular and valuable medicinal plant for a specific condition or use category was calculated using fidelity level (FL) using the formula:

FL(%)=(ΣIp/ΣIu)*100 (4)

Where ∑Ip is the proportion of informants who cited or mentioned using a medicinal plant to treat a specific disease category, and ∑Iu is the total number of informants who cited the plant for any other use or purpose. ^26^ ^–^ ^28^ FL was calculated separately for each disease category to identify dominant therapeutic indications.

***Use Value.*** To determine the relative importance of each medicinal plant, the Use Value (UV) index was calculated following established ethnobotanical methods. ^26^ ^–^ ^28^

UV=(ΣUr/ΣN) (5)

∑Ur is the number of use reports, citations, or mentions by each informant for a particular species, and ∑N is the total number of informants who participated in the study.

To avoid overestimation, when an informant cited the same species for multiple disease categories, only one use report was counted for that informant. Higher UV values indicate greater cultural importance.

### Inferential statistical analysis

Inferential analyses were conducted to examine relationships among key quantitative indices. Spearman’s rank correlation coefficient was used to assess associations between RFC and UV, due to the non-normal distribution of ethnobotanical data. Statistical significance was evaluated at p < 0.05.

Differences in RFC among disease categories were assessed using the non-parametric Kruskal–Wallis test. Pairwise comparisons were performed using Dunn’s post-hoc test with Bonferroni correction.

## Results and discussion

### Demographic characteristics of informants

A total of 219 informants participated in the study, comprising 115 females (53%) and 104 males (47%) ( Table 1). The slight predominance of female respondents suggests that women play a central role in the preservation and transmission of ethnomedicinal knowledge within the Talensi community. Similar gender patterns have been reported in other ethnobotanical studies in West Africa, where women are often primary caregivers and custodians of household healthcare practices. ^29^ ^–^ ^31^

**Table 1.** Demographic information of informants.

Parameter	Category	Number_of_ informants	Percent_ informants
**Gender**	Male	104	47
	Female	115	53
**Age (in years)**	30–50	60	23
	51–60	117	53
	Above 70	42	19
**Educational level**	None	188	86
	Elementary	9	4
	Junior high	8	4
	Secondary	6	3
	Tertiary	8	4
**Religion**	Christian	71	32
	Islamic	6	3
	Traditionalist	131	60
	Others	11	5
**Occupation**	Civil servant	7	3
	Trader	42	19
	Farmer	166	76
	NA	4	2

*Note:* NA = Not specified.

The majority of informants were within the age range of 51–60 years (53%), followed by those above 70 years (19%) and those between 30–50 years (23%). The dominance of older respondents indicates that traditional medicinal knowledge is largely concentrated among senior community members, reflecting cumulative experiential learning and intergenerational knowledge transfer. Although individuals below 30 years were not included in the sampling design, several informants informally reported that younger community members show limited interest in traditional medicine, often preferring biomedical healthcare, in line with other works. ^29^ ^–^ ^31^ This observation, however, was not formally assessed in the present study.

Educational attainment was generally low, with 86% of respondents having no formal education. This highlights the oral and experiential nature of ethnomedicinal knowledge transmission, which is primarily maintained through cultural practice rather than formal schooling. With regards to religious affiliation, most informants are traditionalists (60%), followed by Christians (32%) and Muslims (3%). The dominance of traditional religious affiliation may contribute to the persistence of plant-based healing systems, as such belief systems are often closely integrated with indigenous medical practices.

Farming was the dominant occupational activity (76%), reflecting the agricultural nature of the study area and facilitating frequent interaction with local vegetation. This occupational profile enhances informants’ exposure to medicinal plant resources and strengthens the ecological validity of the recorded ethnobotanical data.

### Influence of demographic factors on medicinal plant knowledge

Beyond descriptive patterns, inferential statistical analyses were conducted to assess whether ethnobotanical knowledge ( Figure 2), measured as the number of medicinal plant species cited per informant, differed significantly across demographic groups.

**Figure 2.**
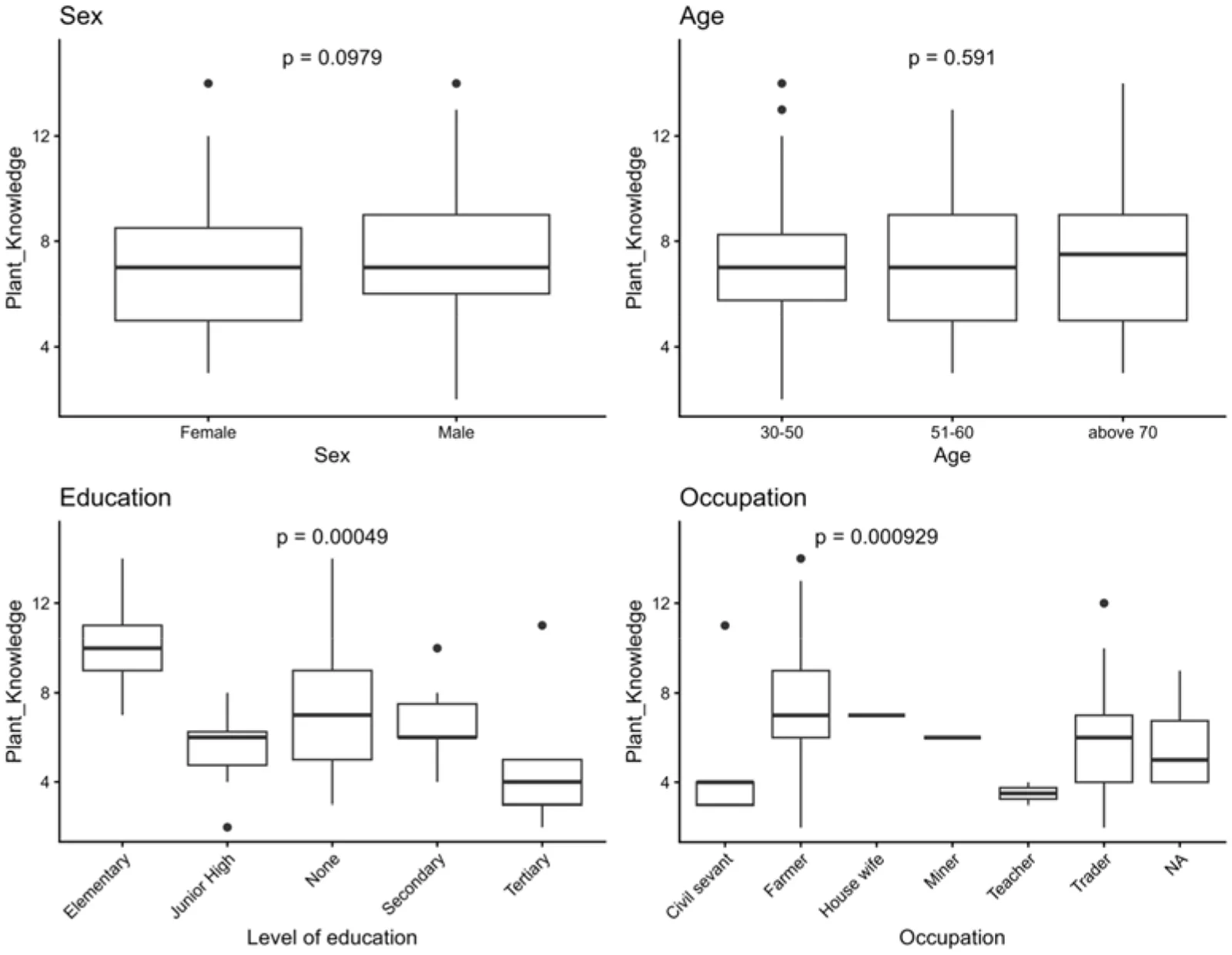
Graph of showing the influence of Demographic Factors on Medicinal Plant Knowledge of informants in the Talensi District.

Although females constituted a slightly higher proportion of respondents, no statistically significant difference was observed in medicinal plant knowledge between male and female informants (Wilcoxon test, p = 0.098). This suggests that ethnomedicinal knowledge is broadly shared between genders in the Talensi District, even though women may be more visibly involved in healthcare-related activities. Our result is in line with other researchers who also found no significant difference in medicinal plant knowledge among different genders. ^32^ ^,^ ^33^ Similarly, no significant differences in plant knowledge were detected across age groups (Kruskal–Wallis test, p = 0.591), indicating that adults across different age categories retain comparable levels of ethnomedicinal knowledge. This finding implies that, despite concerns about generational knowledge erosion, traditional medicinal knowledge remains relatively well distributed among adult members of the community.

In contrast, educational level showed a statistically significant influence on medicinal plant knowledge (Kruskal–Wallis test, p < 0.001). Informants with no formal education exhibited higher plant knowledge scores compared to those with secondary or tertiary education. This pattern suggests that increased exposure to formal education may be associated with reduced dependence on traditional medicinal systems, potentially due to greater reliance on modern healthcare. It must be noted that our findgings contrast with a study in philipines. ^32^

Occupation also significantly influenced medicinal plant knowledge (Kruskal–Wallis test, p < 0.05), with farmers demonstrating higher knowledge levels than other occupational groups. This finding varifies the importance of sustained interaction with the natural environment in shaping ethnobotanical knowledge and supports the role of farming communities as key reservoirs of medicinal plant knowledge.

Religious affiliation did not significantly affect medicinal plant knowledge (p > 0.05), indicating that traditional medicinal practices persist across different belief systems in the study area.

These findings demonstrate that while demographic composition influences participation patterns, ethnomedicinal knowledge in the Talensi District is primarily shaped by ecological interaction and sociocultural engagement rather than gender or age alone.

### Medicinal plant diversity and floristic composition

A total of 46 medicinal plant species belonging to 46 genera and 27 botanical families were documented in the Talensi District. These documented species reflect a savanna-dominated floristic composition, with Fabaceae, Combretaceae, Meliaceae, and Malvaceae emerging as the most represented families. The prominence of these families corresponds with the structural and taxonomic characteristics of the Sudan savanna vegetation, ^34^ where drought-tolerant woody taxa form a major component of the flora.

Growth habit analysis indicated that trees and shrubs constitute the majority of recorded medicinal species ( Figure 3). Dominant woody taxa such as *Khaya senegalensis*, *Pseudocedrela kotschyi* (Schweinf.) Harms, *Vitellaria paradoxa* C.F. Gaertn, and *Diospyros mespiliformis* Hochst. ex A. DC. were frequently cited, emphasizing the central role of perennial savanna species in the local treatment system. Moreover, herbaceous species and climbers, including *Portulaca oleracea* L, *Mimosa pudica* L., and *Paullinia pinnata* L., were comparatively fewer and were typically associated with more specialised therapeutic applications. The predominance of woody perennials suggests that medicinal plant knowledge in the Talensi District is closely aligned with the dominant life forms of the surrounding vegetation.

**Figure 3.**
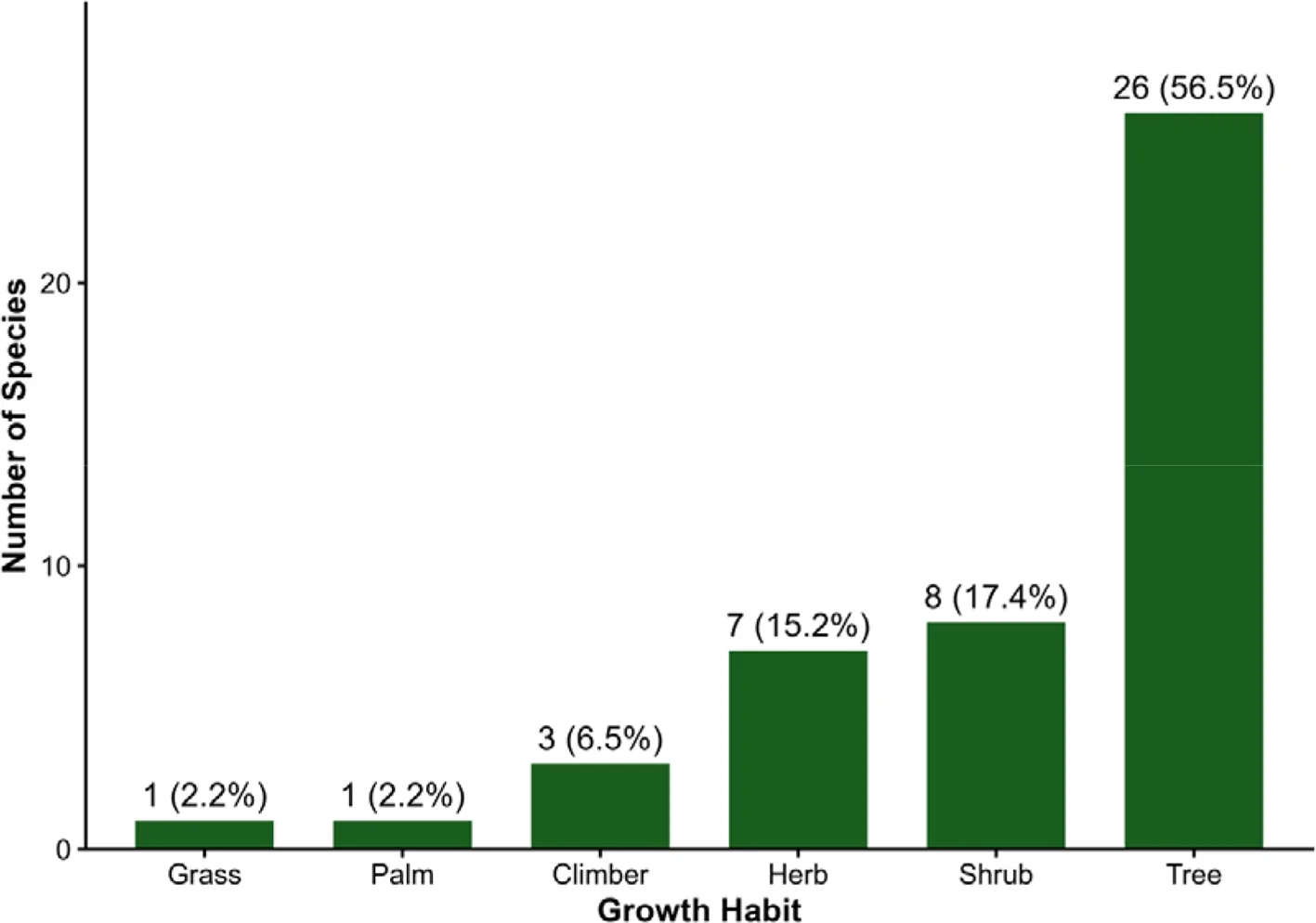
Distribution of growth habits of medicinal plant species documented in the Talensi District (n = 46).

Although most documented species are widely distributed across West Africa, their ethnomedicinal applications reflect locally contextualised knowledge systems shaped by ecological availability and cultural practice. The frequent use of bark and roots from woody species particularly *Securidaca longepedunculata Fresen*, *Khaya senegalensis* (Desr.) A. Juss., and *Pseudocedrela kotschyi*—raises important sustainability considerations. Root and bark harvesting can impose significant pressure on slow-growing savanna trees, potentially affecting regeneration dynamics. ^35^ These findings call for integration of conservation measures, including regulated harvesting and community-based resource management, into traditional medicinal practices.

### Plant part value

The selection of plant parts for remedy preparation varied according to disease category ( Figure 4). Overall, roots were the most frequently cited plant part for snakebite (58.8%), gastrointestinal disorders (53.6%), and dermatological conditions (74.1%), whereas leaves were predominantly used for malaria treatment (83.1%). The dominance of root use for snakebite, gastrointestinal, and skin-related conditions suggests a strong cultural preference for plant tissues perceived to contain higher concentrations of bioactive compounds. Similar patterns have been widely reported in African ethnobotanical studies, where roots are traditionally regarded as storage organs rich in secondary metabolites such as alkaloids and phenolics, making them suitable for treating severe or acute conditions. ^36^ ^–^ ^39^ Representative photographs of medicinal plant species are provided in Appendix Figures A1–A4.

**Figure 4.**
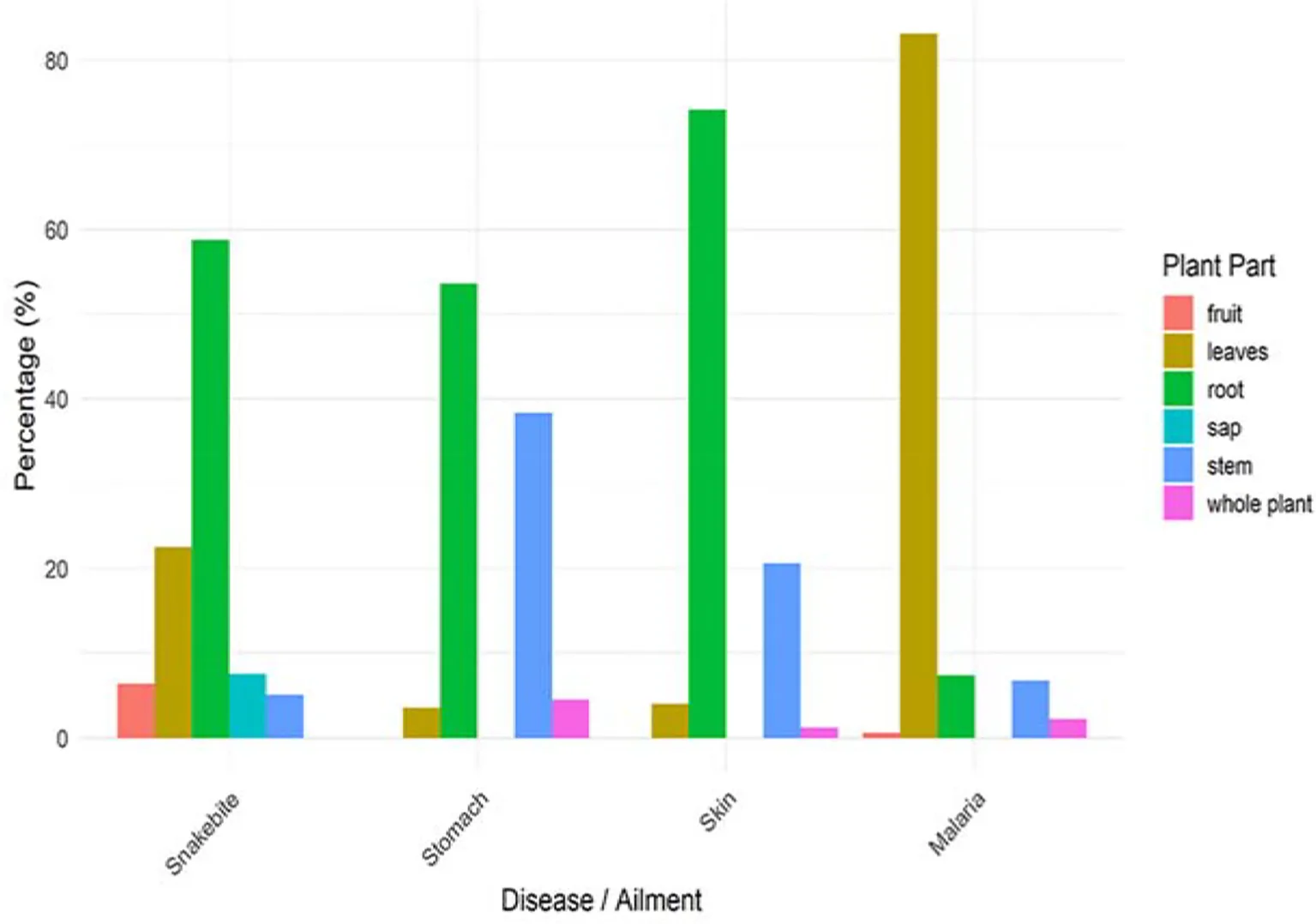
Plant part cited for each ailment.

In contrast, the overwhelming preference for leaves in malaria treatment reflects both pharmacological and ecological considerations. Leaves are commonly used in antimalarial herbal preparations and are known to contain diverse bioactive compounds with antiplasmodial activity. ^40^ ^,^ ^41^ Moreover, leaf harvesting is generally considered more sustainable than root extraction, as it does not compromise plant survival, thereby supporting long-term conservation of medicinal plant resources. ^42^ The intensive reliance on roots for several disease categories may therefore increase pressure on frequently harvested species, emphasizing the need for conservation-oriented harvesting practices.

### Methods for remedy preparation

The methods of preparing herbal remedies varied across disease categories and reflect local perceptions of pharmacological effectiveness and traditional drug delivery strategies ( Figure 5). Overall, boiling/decoction was the most commonly employed method, particularly for gastrointestinal disorders (76.5%) and malaria (94.8%), indicating a strong reliance on water-based extraction techniques.

**Figure 5.**
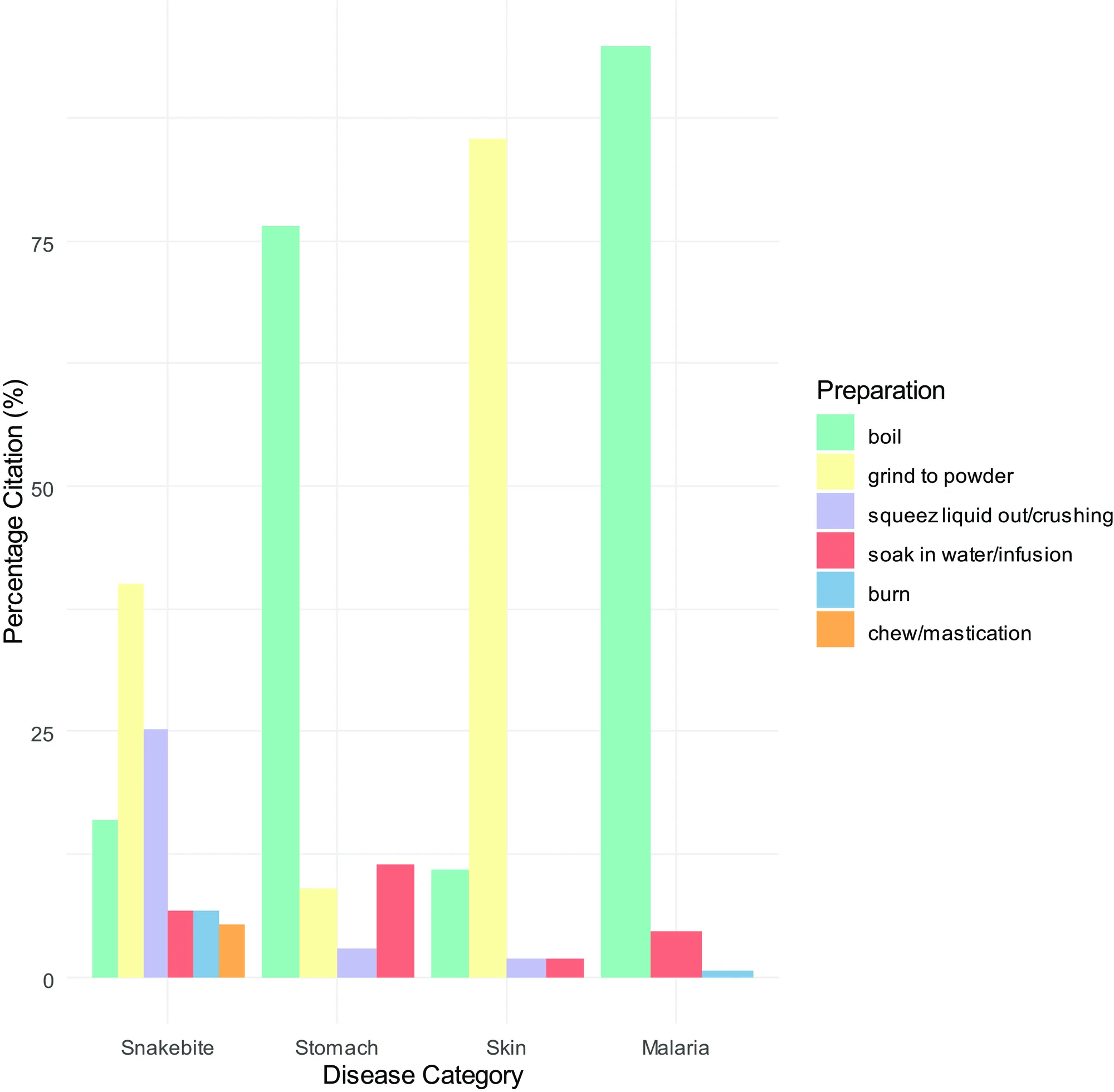
Percentage citation of the mode of preparation of the plant for each ailment category.

The dominance of decoction suggests that informants prioritize the extraction of heat-stable, water-soluble bioactive compounds, such as flavonoids, alkaloids, and phenolic constituents. This practice is consistent with ethnopharmacological literature, which reports that thermal processing enhances the release and bioavailability of active compounds in medicinal plants. ^42^ ^–^ ^44^ The preference for warm preparations further reflects traditional beliefs that hot decoctions are more effective for treating internal ailments.

In contrast, remedies for dermatological conditions were predominantly prepared as powders or pastes (85.5%), a method that facilitates topical application and localized therapeutic action. Grinding increases the surface area of plant material, thereby enhancing the interaction between bioactive compounds and affected tissues. Similar preparation strategies have been widely documented in traditional dermatological practices. ^45^

Preparation methods for snakebite were more diverse, with a combination of grinding, squeezing, burning, chewing, and soaking, reflecting the urgency associated with treating this life-threatening condition. These approaches aim at rapid extraction and immediate application of active compounds, which is consistent with reports from other ethnobotanical studies on snakebite management. ^46^ ^,^ ^47^

### Mode of administration

Three main routes of administration were identified for herbal remedies in the study area: oral, topical, and steam bath ( Figure 6).

**Figure 6.**
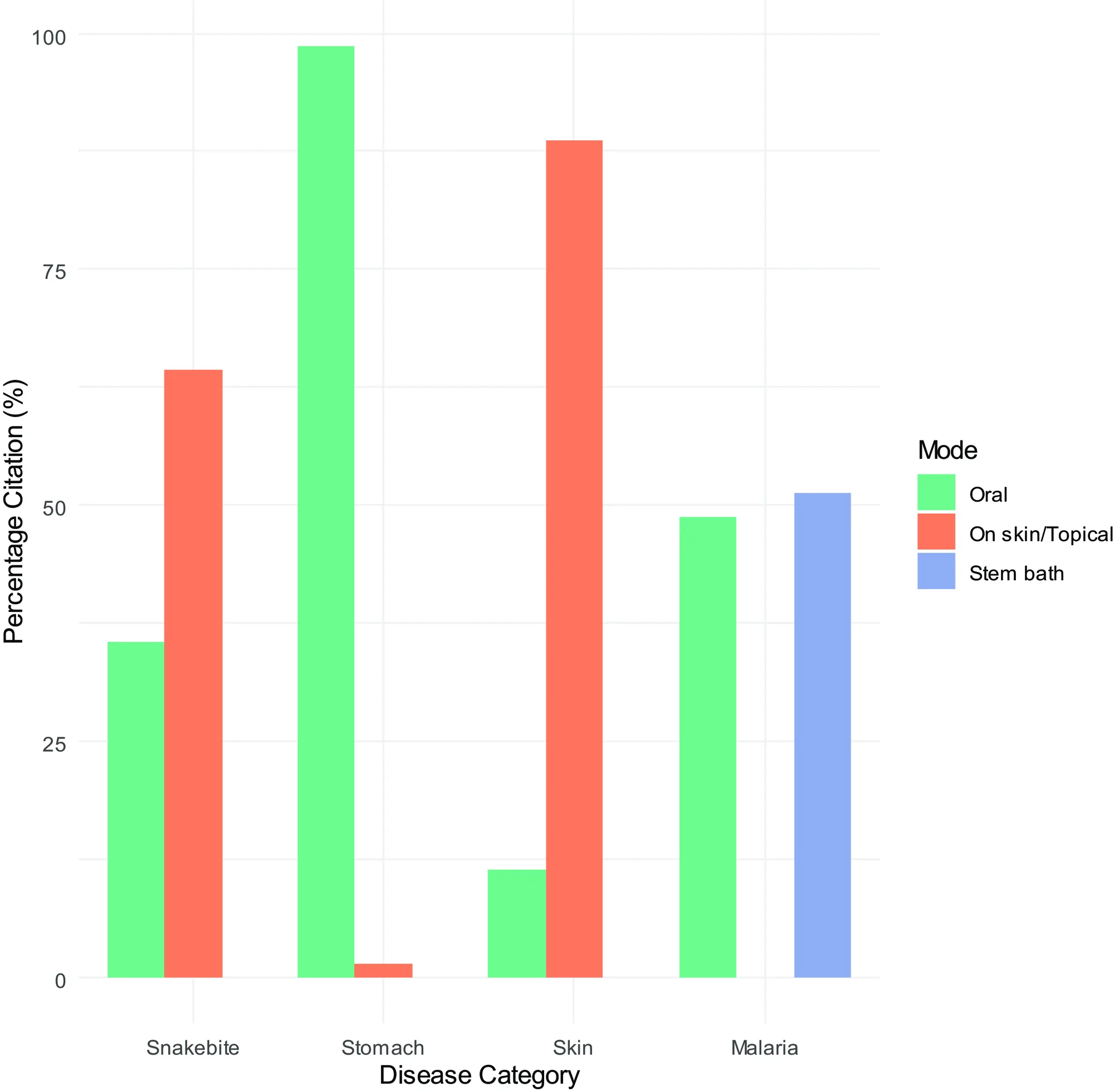
Percentage citation of mode of administration of plant for each ailment category.

The choice of administration route varied systematically with disease category and reflected local perceptions of disease pathology, urgency, and therapeutic target.

Oral administration was overwhelmingly dominant for gastrointestinal disorders (97.3%), indicating strong recognition that internal ailments require systemic treatment. The widespread use of oral decoctions and infusions suggests an understanding that direct gastrointestinal absorption enhances therapeutic effectiveness, a pattern commonly reported in ethnomedicinal systems for digestive conditions. ^48^ ^,^ ^49^

In contrast, dermatological conditions were primarily managed through topical application (88.6%), reflecting the need for localized treatment at the site of infection or inflammation. Topical delivery allows direct interaction between bioactive compounds and affected tissues and is consistent with traditional dermatological practices across different cultural contexts. ^45^

Malaria treatment involved a combination of steam bath (51.3%) and oral administration (48.7%), indicating a dual therapeutic strategy. Steam baths are traditionally used to alleviate external symptoms such as fever and body aches, while oral remedies target systemic parasitic infections. This integrative approach aligns with ethnomedical practices for febrile illnesses, where both symptomatic relief and systemic treatment are emphasized. ^50^ ^,^ ^51^

For snakebite, topical application was the most frequently cited route (62.1%), highlighting the urgency of neutralizing venom at the bite site, while oral remedies (37.9%) were used to manage systemic effects such as pain, inflammation, and tissue damage. Similar patterns have been reported in ethnopharmacological studies on snakebite management, where topical and oral routes are combined to address both local and systemic consequences of envenomation. ^37^ ^,^ ^38^ ^,^ ^46^

### Informant Consensus Factor

The Informant Consensus Factor (ICF) was used to assess the level of agreement among informants regarding the use of medicinal plants for each disease category ( Table 2).

**Table 2.** Informant Consensus Factor of the various disease categories.

Category of diseases	Nu	Nt	Informant consensus factor
Snake bite	88	14	0.89
Stomach related ailments/gastrointestinal disorders	788	29	0.96
Dermatological disorders/skin related diseases	357	26	0.93
Malaria	334	15	0.96

Overall, high ICF values were recorded across all categories, indicating a strong degree of shared ethnobotanical knowledge within the Talensi community. Gastrointestinal disorders and malaria exhibited the highest ICF values (0.96 and 0.96, respectively), reflecting a particularly strong consensus on plant species used for these conditions. This suggests that remedies for these ailments are culturally well established, widely transmitted, and perceived as reliable within the community. High consensus in these categories may also be linked to their high prevalence and frequent occurrence, which promotes repeated validation of specific remedies through collective experience.

Dermatological conditions also showed a high level of agreement (ICF = 0.93), indicating relatively stable and shared knowledge on treatments for skin-related infections and inflammations.

In contrast, snakebite presented the lowest ICF value (0.89), although still relatively high compared to values reported in many ethnobotanical studies. The comparatively lower consensus for snakebite may reflect the specialized and localized nature of knowledge associated with this condition. In the study area, snakebite treatment is often managed by specialist healers, locally referred to as *“waakolog daan”*, who combine herbal remedies with ritual practices. This restricted knowledge of transmission likely contributes to greater variability in reported plant use.

The ICF results indicate that ethnomedical knowledge in the Talensi District is highly structured and socially shared, particularly for common ailments such as gastrointestinal disorders and malaria, while more specialized conditions such as snakebite are characterized by narrower and more heterogeneous knowledge systems.

### Relative Frequency of Citation and Fidelity Level

Relative Frequency of Citation (RFC) and Fidelity Level (FL) were used to evaluate both the cultural prominence and the specificity of medicinal plants employed in snakebite management. While RFC reflects how widely a plant is known and relied upon, FL indicates the degree to which its use is restricted to a particular ailment. The combined use of these indices allows differentiation between culturally dominant species and those that are highly specialized.

In the present study, RFC values for snakebite ranged from 1.1% to 46.6% ( Table 3), indicating substantial variation in the level of community reliance on individual species.

**Table 3.** Information of plants used for the treatment of snakebite.

Voucher number	Medicinal plant	Part of plant used	Mode of preparation	Mode of administration	Family	Local name	Citations	RFC	FL	Other uses	Side effect
KNUST/HM1/2023/R006	*Securidaca longepedunculata* Fresen.	Root bark, stem bark or leaves	Soak in water, or crush to power	Drink infusion and apply past to affected area	Polygalaceae	Pelig	41	46.6	20.9	Boil, inflammation, pains, insect and snake repellant, stomach pains, pains in bones, deliver placenta, body weakness, de-clot blood after birth	Yes (causes miscarriage in pregnant women, excess can cause diarrhea)
KNUST/HM1/2023/F009	*Carica papaya* L.	Fruit (unripe)	Chew	Orally	Caricaceae	Pawpaw	3	3.4	4.9	Fever	No
KNUST/HM1/2016/SB013	*Piliostigma reticulatum* (DC.) Hochst.	Leaves, root or bark	Burn together with other non-plant materials	Orally	Fabaceae	Ba-an	13	14.8	68.4		No
KNUST/HM1/2018/L052	*Vitellaria paradoxa* C.F. Gaertn.	Leaves or gum/sap	Sap is used to remove the tooth of snake. Leaves are chewed and applied to affected area		Sapotaceae	Ta-an	10	11.4	31.3	Diarrhea, kokoo	No
KNUST/HM1/S002	*Cola nitida* (Vent.) Schott & Endl.	Fruit	Chew swallow and apply some to the affected area		Sterculiaceae	Kolanut	4	4.5	100.0		
KNUST/HM1/2025/SB013	*Khaya senegalensis* (Desr.) A. Juss.	Leaves	Burn or boil	Oral	Meliaceae	Kok	2	2.3	0.8	Stomach pains, inflammation	No
KNUST/HM1/2025/L026	*Adesmia incana var. grisea Hook. f.*	Root, leaves	Chew	Apply on skin and oral	Fabaceae	Kbantiug	4	4.5	7.3		No
KNUST/HM1/2025/L024	*Mitragyna inermis* (Willd.) Kuntze	Root	Grind	Topical and oral	Rubiaceae	Yeblig	1	1.1	1.3	Provides strength for pregnant women	No
KNUST/HM1/2025/L026	*Cassia sieberiana* DC.	Root	Grind	Topical and oral	Fabaceae	Kolkbawure	1	1.1	2.2	Diarrhea	No
KNUST/HM1/2025/SB012	*Pseudocedrela kotschyi* (Schweinf.) Harms	Root	Infusion, burning, crush to powder	Oral and topical	Meliaceae	Shiarig	4	4.5	1.7	Stomach pains, inflammation	No
KNUST/HM1/2025/L028	*Ziziphus spina-christi * (L.) Desf.	Laves, root, stem	No idea	No idea	Rhamnaceae	Lien	1	1.1	5.9		No
KNUST/HM1/2017/L005	*Lannea microcarpa* Engl. & K. Krause	Root, stem, leaves	No idea	No idea	Anacardiaceae	Sisablik	1	1.1	9.1		No
KNUST/HM1/2025/L016	*Adansonia digitata* L.	Leaves	Burn with other plants ( *Adesmia incana* var. grisea Hook. f.)	Topical and oral	Malvaceae	Tuu/Tua	2	2.3	66.7		No
KNUST/HM1/2019/L019	*Portulaca oleracea* L.	Leaves	No idea	No idea	Portulacaceae	Baatobzeriba	1	1.1	100.0		No

### Plants for snakebite treatment

*Securidaca longepedunculata* Fresen emerged as the most culturally salient species, with the highest RFC (46.6%), suggesting that it occupies a central position in local snakebite ethnotherapy. The prominence of *S. longepedunculata* reflects both widespread recognition and repeated validation of its perceived efficacy within the community. According to the informants, root bark of *S. longepedunculata* is also placed in bath-house, rooms, and farmhouses to repel snakes, scorpions, and insects in addition to use for treatment of snakebite and all manner of diseases.

However, *S. longepedunculata* exhibited a relatively moderate FL value (20.9%), indicating that although it is frequently cited, it is also used for a wide range of other ailments. This pattern suggests that highly cited plants are often therapeutically versatile rather than disease-specific, functioning as multipurpose remedies within the traditional medicinal knowledge system.

In contrast, species such as *Cola nitida* (Vent.) Schott & Endl. and *Portulaca oleracea* L. showed very high FL values (100%) despite relatively low RFC values, indicating strong consensus on their specific use for snakebite but limited overall popularity. These plants can therefore be considered culturally specialized remedies, likely maintained within narrower knowledge networks or used in specific contexts.

The coexistence of plants with high RFC but moderate FL, and others with low RFC but high FL, highlights a dual structure within the Talensi ethnomedical system. A core set of widely known, multifunctional species and a peripheral set of highly specific remedies. This pattern is consistent with ethnobotanical models reported in other traditional medical systems, where knowledge is organized around both generalist and specialist plant resources.

Collectively, the RFC–FL patterns for snakebite indicate that treatment knowledge is structured around a small number of culturally dominant species complemented by a diverse pool of specialized plants, reflecting both collective consensus and localized expertise in snakebite management.

### Plants for stomach-related diseases

The distribution of Relative Frequency of Citation (RFC) and Fidelity Level (FL%) values for stomach-related diseases indicates a structured and hierarchical ethnobotanical knowledge system within the Talensi community. A small group of species combines both high RFC and high FL values, reflecting plants that are not only widely recognized but also culturally validated as reliable remedies for gastrointestinal conditions.

Species such as *Khaya senegalensis* (Desr.) A. Juss. and *Pseudocedrela kotschyi* (Schweinf.) Harms emerged as the most culturally salient taxa, exhibiting the highest RFC values in this category. Their prominence suggests that they occupy central positions in the local pharmacopoeia and are perceived as multipurpose therapeutic resources. This pattern is consistent with ethnobotanical studies from other parts of West Africa, where these species are repeatedly reported for their antimicrobial, anti-inflammatory, and wound-healing properties, particularly in the management of gastrointestinal and infectious diseases. ^52^ ^–^ ^55^

In contrast, species such as *Mitragyna inermis* (Willd.) Kuntze and *Combretum molle* R. Br. ex G. Don. exhibited comparatively lower RFC values but very high FL percentages, indicating a high degree of specificity in their use. This combination suggests that while these plants are not among the most frequently cited overall, they are strongly associated with stomach-related ailments and are considered particularly effective within this therapeutic domain. Similar patterns of high FL coupled with moderate or low RFC have been interpreted in other ethnobotanical studies as indicators of specialized medicinal roles rather than general-purpose use. ^32^ ^,^ ^56^ ^,^ ^57^

Furthermore, several species, including *Detarium senegalense* J.F. Gmel. and *Cochlospermum planchonii* Hook., displayed perfect FL values despite low citation frequencies. This pattern reflects highly specialized knowledge restricted to a smaller subset of informants, possibly traditional healers or households with inherited ethnomedical expertise. Such species represent culturally conserved remedies that may be at risk of knowledge erosion but could offer valuable pharmacological potential for targeted gastrointestinal therapies.

The observed RFC–FL structure suggests that ethnomedicinal knowledge related to gastrointestinal disorders in the Talensi District is characterized by a dual system: a core group of widely used multipurpose species and a peripheral group of highly specialized plants. This hierarchical organization of knowledge aligns with patterns reported in quantitative ethnobotanical studies from other regions, where medicinal systems tend to concentrate around a limited number of culturally dominant species while preserving specialized remedies for specific conditions. ^58^ ^,^ ^59^

**Table 4.** Information on plants used to treat stomach-related diseases.

Voucher number	Scientific name	Family	Local name	Part of plant used	Mode of preparation	Mode of administration	Frequency of citation	RFC	FL	Other ailments	Side effect
KNUST/HM1/2023/R006	*Securidaca longepedunculata Fresen.*	Polygalaceae	Pelig	Root bark, stem bark	Boil, mix dry powder with warm water/porridge	Oral	23	2.9	11.7	Inflammation	Yes, causes abortion
KNUST/HM1/2025/SB013	*Khaya senegalensis* (Desr.) A. Juss.	Meliaceae	Kok	Root bark, stem bark	Boil, mix dry powder with warm water	Oral	149	18.9	63.1	Anthrax, boil, waist pains, aphrodisiac, wound healing	No
KNUST/HM1/2025/L024	*Mitragyna inermis* (Willd.) Kuntze	Rubiaceae	Yeblig	Root, stem, leaves	Boil	Oral	75	9.5	93.8	Swollen legs, waist pains, boosts appetite, aids delivery, additive to fracture treatment	No
KNUST/HM1/2025/L025	*Combretum molle* R. Br. ex G. Don	Combretaceae	Gberig	Root, stem	Boil	Oral	58	7.4	98.3	Appetite booster, toothache, waist pains	No
KNUST/HM1/2025/L027	*Nauclea orientalis* (L.) L.	Rubiaceae	Gu-un	Root, stem		Oral	36	4.6	83.7	Adjuvant to snake-bite treatment	No
KNUST/HM1/2025/SB012	*Pseudocedrela kotschyi* (Schweinf.) Harms	Meliaceae	Shiarig/Shiarit	Stem, root	Chew and swallow, mix powdered dry sample with water	Oral	131	16.6	55.7	Anthrax, inflammation, boil, antimicrobial, snake-bite additive, headache, wound healing	No
KNUST/HM1/2020/SB010	*Anogeissus leiocarpus* (DC.) Guill. & Perr.	Combretaceae	Cii	Leaves, stem, root	Boil	Oral	49	6.2	94.2	Cough	No
KNUST/HM1/2017/SB011	*Terminalia macroptera* Guill. Perr.	Combretaceae	Podaa	Root, stem	Boil	Oral	39	4.9	92.9	Toothache	No
KNUST/HM1/2018/L058	*Vitex doniana* Sweet	Lamiaceae	Aa-rig	Stem, root	Boil, infusion	Oral	23	2.9	92.0	Prevent miscarriage	No
KNUST/HM1/2017/L005	*Lannea microcarpa* Engl. & K. Krause	Anacardiaceae	Sisablik	Stem, root	Boil	Oral	9	1.1	81.8	Blood tonic, burns	No
KNUST/HM1/2025/L026	*Adesmia incana* var. grisea Hook. f.	Fabaceae	Kbantiug	Root, whole plant	Boil, infusion, chew	Oral	51	6.5	92.7		No
KNUST/HM1/2017/L001	*Cassia sieberiana* DC.	Fabaceae	Kolkbawure	Stem, root	Boil, infusion	Oral	42	5.3	91.3		No
KNUST/HM1/2025/SB017	*Ficus gnaphalocarpa* (Miq.) Steud. ex A. Rich	Moraceae	Kikan	Stem, root	Boil	Oral	4	0.5	80.0	Cough	No
KNUST/HM1/2025/L029	*Detarium senegalense* J.F. Gmel.	Fabaceae	Kbarig	Stem, root	Boil, infusion	Oral	12	1.5	100.0		No
KNUST/HM1/2019/R002	*Sclerocarya birrea* (A. Rich.) Hochst.	Anacardiaceae	Nanobig	Stem, root, leaves	Boil	Oral	8	1.0	100.0	Cancer of breast and testes	No
KNUST/HM1/2018/L052	*Vitellaria paradoxa* C.F. Gaertn.	Sapotaceae	Ta-an	Stem, root	Boil	Oral	17	2.2	53.1		No
KNUST/HM1/2023/F001	*Parkia biglobosa* (Jacq.) R. Br. ex Don	Fabaceae	Duo	Stem, root	Boil	Oral	11	1.4	73.3		No
KNUST/HM1/2025/SB001	*Diospyros mespiliformis* Hochst. ex A. DC.	Ebenaceae	Gar	Stem, root	Boil, infusion	Oral	6	0.8	100.0	Rib pains	No
KNUST/HM1/2025/L030	*Cochlospermum planchonii* Hook.	Bixaceae/Cochlospermaceae	Bugla	Leaves, root, stem	Boil	Oral	12	1.5	100.0		No
KNUST/HM1/2025/L028	*Ziziphus spina-christi * (L.) Desf.	Rhamnaceae	Lien	Stem, root, fruit	Boil, eat fruit	Oral	11	1.4	64.7		No
KNUST/HM1/2025/L005	*Azadirachta indica* A. Juss.	Meliaceae	Nim tree	Leaves, stem, root	Boil	Oral	6	0.8	4.4		No
KNUST/HM1/2025/L006	*Vernonia amygdalina* Delile	Asteraceae	Suwaka	Leaves	Boil	Oral	1	0.1	33.3		No
KNUST/HM1/2019/R014	*Borassus aethiopum* Mart.	Arecaceae	Karikon	Fruit	Eat fruit	Oral	1	0.1	100.0		No
KNUST/HM1/2019/R006	*Cantinoa americana* (Aubl.) Harley & J.F.B. Pastore	Lamiaceae	Dunkwaq	Whole plan	Boil	Oral	1	0.1	25.0		No
KNUST/HM1/2019/R008	*Acacia sieberiana* DC. var.	Fabaceae (Mimosoideae)	GUOpelug	Whole plant	Boil	Oral	3	0.4	100.0		No
KNUST/HM1/2019/R015	*Psidium guajava* L.	Myrtaceae	Guava	Leaves, root, stem	Boil	Oral	1	0.1	100.0		No
KNUST/HM1/2019/R010	*Dialium indum* L.	Fabaceae	Nolig	Stem, root	Boil	Oral	2	0.3	100.0		No
KNUST/HM1/2019/R011	*Waltheria indica* L.	Malvaceae	Dabirig	Root, stem	Boil	Oral	2	0.3	100.0		No
KNUST/HM1/2016/SB013	*Piliostigma reticulatum* (DC.) Hochst.	Fabaceae	Ba-an	Root, stem, whole plant	Boil	Oral	5	0.6	26.3		No

### Plants used in the treatment of skin-related diseases/dermatological disorders

The distribution of RFC and FL values for dermatological disorders ( Table 5) reveals a structured ethnomedical system characterized by a small group of culturally dominant species alongside a larger set of highly specialized remedies. This pattern suggests that traditional dermatological knowledge in the Talensi District is organized around a limited number of core plants that are widely recognized and frequently used, complemented by species with narrow but well-defined therapeutic roles.

**Table 5.** Information of plants used to treat skin-related diseases.

Voucher number	Scientific name	Family	Local name	Part of plant used	Mode of preparation	Mode of administration	Frequency of citation	RFC	FL (%)	Other treatment	Side effect
KNUST/HM1/2023/R006	*Securidaca longipedunculata* Fresen.	Polygalaceae	Pelig	Root, stem	Grind sample to powder and add water, and powdered boabab leaves to form a paste, also add powdered sample to warm water	Drink infusion and apply paste to affected area	132	37.0	67.3	Headache, diarrhoea, snake & scorpion repellent/bite, all kinds of diseases, removing blood clot, anthrax, ulcer, indigestion	Yes, causes abortion
KNUST/HM1/2025/L027	*Nauclea orientalis* (L.) L.	Rubiaceae	Gu-un	Root, stem	Add water to powdered sample to form a paste	Apply paste to affected area and drink a decoction	7	2.0	16.3	Stomach ache	No
KNUST/HM1/2025/SB013	*Khaya senegalensis* (Desr.) A. Juss.	Meliaceae	Kok	Root, stem	Grind/crush to powder, soak in water	Add water to from a paste and apply to affected area	79	22.1	33.5	Headache, diarrhoea, stomach pains	No
KNUST/HM1/2025/SB012	*Pseudocedrela kotschyi* (Schweinf.) Harms.	Meliaceae	Shiarig	Root, stem	Grind to form powder	Add water to form a paste and apply to affected area, you may drink a decoction in addition	100	28.0	42.6	Snake bite, stomach ache, anthrax, indigestion, ulcer treatment, strengthen teeth, cough	No
KNUST/HM1/2017/SB011	*Terminalia macroptera* Guill. Perr.	Combretaceae	Podaa	Root, stem	Grind to powder, soak in water	Drink decoction and apply a paste to affected area	3	0.8	7.1		No
KNUST/HM1/2019/R016	*Strophanthus hispidus* DC.	Apocynaceae	Yarbik	Root	Burn and add water or shear butter to form a paste	Apply to affected area	1	0.3	100.0	Ringworm, arrow poison, eye treatment	No
KNUST/HM1/2019/R017	*Zingiber officinale* Roscoe	Zingiberaceae	Kakadru/Ginga	Rhizome	Crush/grind	Apply paste to affected area and drink an infusion	1	0.3	100.0		No
KNUST/HM1/2019/R012	*Allium sativum* L.	Amaryllidaceae	Garlic	Bulb, whole plant	Crush	Apply paste to inflamed area	2	0.6	100.0	Ear problem	No
KNUST/HM1/2025/L025	*Combretum molle* R.Br. ex G.Don	Combretaceae	Gberig	Root	Grind to powder and add water to from a paste, soak in water	Drink a decoction, apply paste to affected area	1	0.3	1.7		No
KNUST/HM1/2023/F001	*Parkia biglobosa* (Jacq.) R. Br. ex G. Don	Fabaceae	Duo	Root, stem	Burn, or grind to powder then add water to from a paste	Drink a decoction and apply the paste to affected area	4	1.1	26.7		No
KNUST/HM1/2016/SB013	*Piliostigma reticulatum* (DC.) Hochst.	Fabaceae	Ba-an	Root, stem	Grind to powder and add water to from paste	Apply paste to affected area	1	0.3	5.3		No
KNUST/HM1/2025/L028	*Ziziphus spina-christi * (L.) Desf.	Rhamnaceae	Lien	Stem	Grind to powder and add water to form a paste	Apply paste to affected on skin	5	1.4	29.4	Additive to snake-bite treatment	No
KNUST/HM1/2025/L024	*Mitragyna inermis* (Willd.) Kuntze	Rubiaceae	Yeblig	Root, stem	Crush/grind to powder and add water to form a paste	Apply paste to affected area, you may drink a decoction of it	4	1.1	5.0		No
KNUST/HM1/2019/R018	*Annona senegalensis* Pers.	Annonaceae	Baburig	Stem, root	Grind to powder and add water to form a paste	Apply paste to affected area	1	0.3	100.0		No
KNUST/HM1/2019/R007	*Adansonia digitata* L.	Malvaceae	Tuo	Root, stem	Burn or grind to powder and add water to form a paste or shear butter in the case of the burnt sample	Apply paste to affected area	1	0.3	33.3		No
KNUST/HM1/2020/SB010	*Anogeissus leiocarpus* (DC.) Guill. & Perr.	Combretaceae	Cii	Stem	Grind to powder and add water and baobab to form a paste	Apply paste to affected area	2	0.6	3.8		No
KNUST/HM1/2019/R019	*Phaseolus vulgaris* L.	Fabaceae	Beans/tiya	Leaves	Grind/crush fresh sample	Apply paste to boil, or inflamed area	1	0.3	100.0		No
KNUST/HM1/2019/R020	*Abelmoschus esculentus* (L.) Moench	Malvaceae	Okro/Ma-a-na	Fruit, stem	Crush fresh sample	Apply paste around boil	1	0.3	100.0		No
KNUST/HM1/2018/L052	*Vitellaria paradoxa* C.F. Gaertn.	Sapotaceae	Ta-an	Root, stem	Mix with other plants and grind to powder and add water to form a paste	Apply paste to affected area and drink a decoction	4	1.1	12.5	Diarrhoea	No
KNUST/HM1/2018/L006	*Mangifera indica* L.	Anacardiaceae	Mongo tii	Sap	Squeeze liquid out	Apply sap to inflammation or boil area	1	0.3	1.2		No
KNUST/HM1/2017/L001	*Cassia sieberiana* DC.	Fabaceae	Kolkbawure	Root, stem	Grind to powder and add water to form a paste	Apply on affected area	2	0.6	4.3		No
KNUST/HM1/2025/L005	*Azadirachta indica* A. Juss.	Meliaceae	Nim ti	Leaves	Boil and crush to paste	Drink decoction and apply paste on affected area	1	0.3	0.7		No
KNUST/HM1/2019/R021	*Paullinia pinnata* L.	Sapindaceae	Gerisablig	Root, stem	Grind to powder, Boil	Drink decoction and apply paste to affected area	1	0.3	100.0		No
KNUST/HM1/2019/R022	*Vossia cuspidata* Griff.	Poaceae	Kontinchioh	Whole plant	Crush to paste	Apply paste to inflamed area	1	0.3	100.0		No
KNUST/HM1/2019/R003	*Ficus gnaphalocarpa* (Miq.) Steud. ex A. Rich.	Moraceae	Kikan	Stem	Grind to powder	Apply to affected area	1	0.3	20.0	Diarrhoea, enhance breast-milk flow	No
KNUST/HM1/2019/R013	*Portulaca oleracea* L.	Portulacaceae	Baatob-zariba	Whole plant, leaves	Grind fresh plant	Apply paste around boil or inflammation	1	0.3	100.0		No

*S. longepedunculata* and *P. kotschyi* emerged as the most culturally salient species for skin-related conditions, reflecting their central position in the local traditional knowledge system. Their high RFC values indicate widespread recognition, while their relatively high FL values suggest consistent use for dermatological applications. These species are also widely reported in ethnobotanical studies for their antimicrobial, anti-inflammatory, and wound-healing properties, supporting their perceived efficacy in treating skin infections and inflammatory conditions. ^54^ ^,^ ^55^ ^,^ ^60^

In contrast, several species, including *Zingiber officinale* Roscoe, *Allium sativum* L., *Strophanthus hispidus* DC., and *Annona senegalensis* Pers., exhibited perfect FL values but very low RFC scores. This combination indicates highly specialized medicinal knowledge, restricted to a small subset of informants but associated with strong therapeutic consensus. Similar patterns have been reported in other quantitative ethnobotanical studies, where plants with high FL and low RFC are interpreted as culturally conserved remedies with niche applications rather than general-purpose medicines. ^32^ ^,^ ^59^

The RFC–FL configuration observed in this study suggests that dermatological treatments in the Talensi District are guided by both communal consensus and specialized expertise, reinforcing the importance of combining quantitative indices with qualitative interpretation to fully capture the complexity of traditional medicinal systems.

### Plants for malaria treatment

The RFC–FL structure for malaria remedies reveals a highly organized ethnomedical system dominated by a small group of culturally salient species, supported by a set of specialized but less widely diffused plants. This pattern suggests that malaria management in the Talensi District is largely centered on a core repertoire of well-established remedies, reflecting strong communal consensus and long-term experiential validation.

*Azadirachta indica A. Juss.* and *Mangifera indica* L. emerged as the most culturally dominant species, combining high RFC with very high FL values. This configuration indicates that these plants are not only widely known but are also consistently associated with malaria treatment across informants. Similar dominance of *A. indica* has been widely reported in West Africa and other malaria-endemic regions, where it is recognized for its strong antiplasmodial activity and pharmacological relevance. ^40^ ^,^ ^41^ ^,^ ^61^ ^,^ ^62^ The prominence of these species supports the ethnopharmacological principle that highly cited plants often represent biologically active taxa that merit priority for pharmacological screening.

In contrast, species such as *Moringa oleifera* Lam, *Mimosa pudica* L., *Ananas comosus* (L.) Merr. and *Cantinoa americana* (Aubl.) Harley & J.F.B. Pastore exhibited perfect or near-perfect FL values but low RFC scores. This pattern reflects highly specialized knowledge, restricted to a limited number of informants but associated with strong therapeutic consensus. Such plants are typically interpreted as culturally conserved remedies maintained within specific families or healer lineages, rather than widely diffused community medicines. Comparable RFC–FL configurations have been reported in quantitative ethnobotanical studies where low-frequency but high-fidelity species are considered important reservoirs of localized medicinal knowledge. ^32^ ^,^ ^57^

Conversely, species such as *Vitellaria paradoxa C.F. Gaertn.* and *K. senegalensis* displayed both low RFC and low FL values, suggesting marginal roles in malaria management within the study area. Their limited importance may reflect broader therapeutic specialization in other disease categories, reduced perceived efficacy for malaria, or shifting treatment preferences associated with increased access to biomedical antimalarial therapies.

**Table 6.** Information about plants used for the treatment of malaria.

Voucher number	Scientific name	Family	Local name	Part of plant used	Mode of preparation	Mode of administration	Frequency of citation	RFC	FL (%)	Other treatment	Side effect
KNUST/HM1/2025/L005	*Azadirachta indica A. Juss.*	Meliaceae	Nim tii	Leaves	Boil	Oral and steam bath	130	38.8	94.9		No
KNUST/HM1/2023/F009	*Carica papaya* L.	Caricaceae	Pawpaw	Leaves	Boil	Oral and stem bath	58	17.3	95.1		No
KNUST/HM1/2018/L006	*Mangifera indica* L.	Anacardiaceae	Mongo	Leaves, whole plant	Boil	Oral and steam bath	83	24.8	98.8		No
KNUST/HM1/2018/L052	*Vitellaria paradoxa C.F. Gaertn.*	Sapotaceae	Ta-an	Stem, leaves	Boil	Oral and steam bath	1	0.3	3.1		No
KNUST/HM1/2019/R023	*Ananas comosus* (L.) Merr.	Bromeliaceae	Pineapple	Leaves	Boil	Oral and steam bath	1	0.3	100.0		No
KNUST/HM1/2019/R009	*Vernonia amygdalina* Delile	Asteraceae	Suwaka	Leaves	Boil	Oral and steam bath	2	0.6	66.7		No
KNUST/HM1/2017/L005	*Lannea microcarpa Engl. & K. Krause*	Anacardiaceae	Sisablik	Steam, root	Boil	Oral and steam bath	1	0.3	9.1		No
KNUST/HM1/2024/L013	*Moringa oleifera* Lam.	Moringaceae	Moringa	Leaves, stem	Boil	Oral and steam bath	43	12.8	100.0		No
KNUST/HM1/2018/L058	*Vitex doniana* Sweet	Lamiaceae	Aa-rig	Root, stem	Boil	Oral and steam bath	2	0.6	8.0		No
KNUST/HM1/2017/L001	*Cassia sieberiana* DC.	Fabaceae	Kolbawure	Stem	Boil	Oral and steam bath	1	0.3	2.2		No
KNUST/HM1/2017/L009	*Jatropha gossypiifolia* L.	Euphorbiaceae	Black flowers/flower sabil	Leaves	Boil	Oral and steam bath	1	0.3	100.0		No
KNUST/HM1/2020/SB010	*Anogeissus leiocarpus* (DC.) Guill. & Perr.	Combretaceae	Cii	Leaves, stem bark	Boil	Oral and steam bath	1	0.3	1.9		No
KNUST/HM1/2025/SB013	*Khaya senegalensis* (Desr.) A. Juss.	Meliaceae	Kok	Steam, leaves	Boil	Oral and steam bath	6	1.8	2.5		No
KNUST/HM1/2019/R006	*Cantinoa americana* (Aubl.) Harley & J.F.B. Pastore	Lamiaceae	Dunkwaq	Whole plant	Burn for smoke to repel mosquitoes	-	3	0.9	75.0		No
KNUST/HM1/2019/R024	*Mimosa pudica* L.	Fabaceae	Wabur-kasakur	Whole plant	Boil	Oral and steam bath	1	0.3	100.0		No

### Use Reports and Use Value (UV) analysis

The Use Reports (UR) and Use Value (UV) analysis offer critical insights into the cultural significance and therapeutic applications of medicinal plants within the study area. These metrics reflect the frequency of plant use and indicate their perceived importance in traditional medicine. The findings highlight a spectrum of usage, ranging from highly utilized species to those with specialized or declining roles, providing a comprehensive view of the community’s ethnobotanical practices. Figure 7 shows the results of the analysis. However, plant with use value less than 0.05 not shown in the graph because of space.

**Figure 7.**
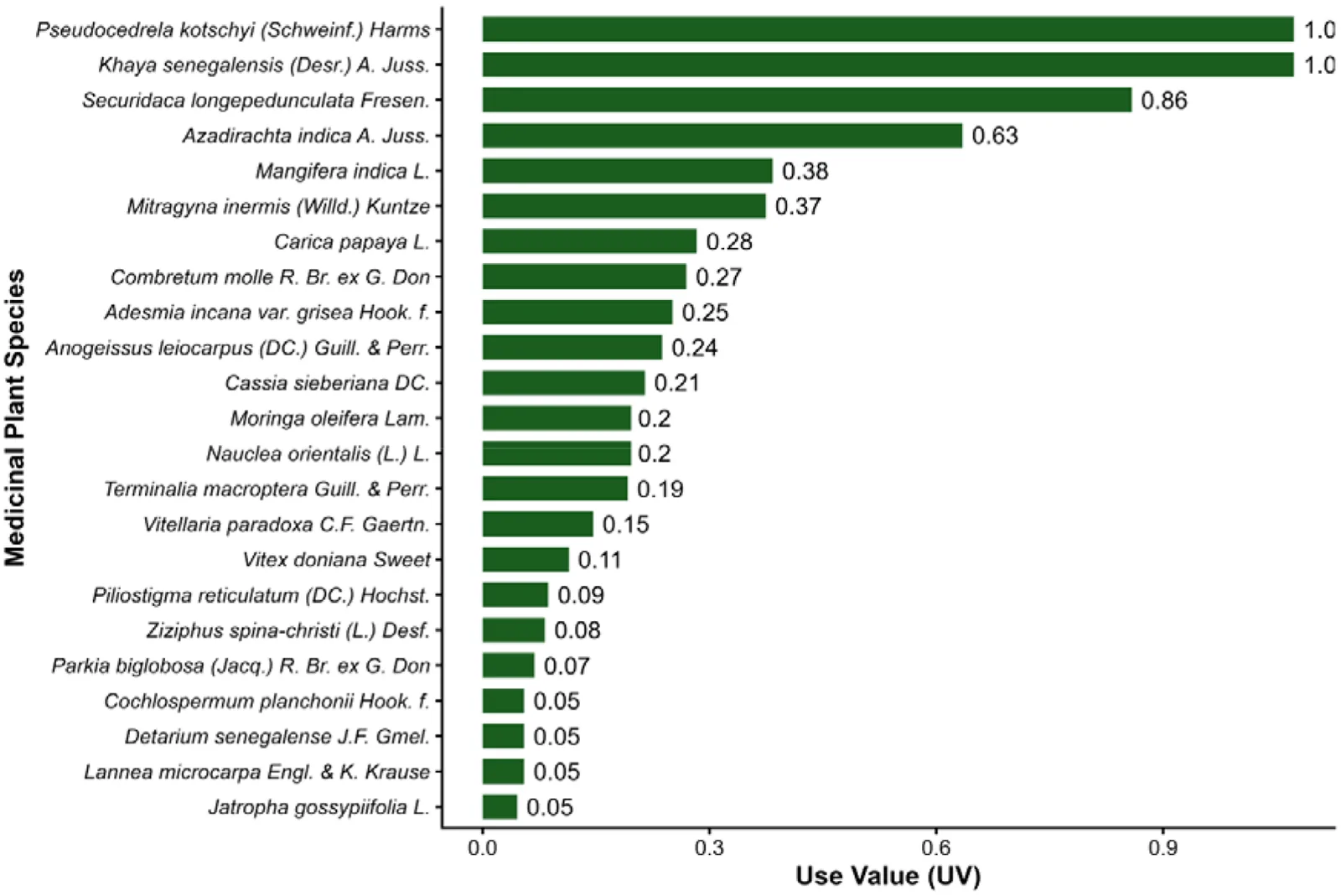
Use value of plant species in the Talensi District.

The Use Value (UV) analysis reveals a strongly structured ethnomedical system in which a limited number of species form the core of medicinal practice in the Talensi District, while a larger group of plants occupies peripheral or specialized roles. This knowledge distribution is characteristic of mature traditional medical systems, where repeated empirical use over generations leads to the consolidation of a culturally dominant pharmacopoeia.

The highest UV values were recorded for *K. senegalensis* and *P. kotschyi*, indicating that these species are not only widely known but are also used across multiple disease categories and therapeutic contexts. Plants with high UV are typically regarded as therapeutically reliable and culturally indispensable, since they combine frequency of citation with diversity of application. The central position of these two species in the Talensi pharmacopeia is consistent with their documented antimicrobial, anti-inflammatory and wound-healing properties reported in previous pharmacological and ethnobotanical studies. ^52^ ^,^ ^53^ ^,^ ^55^ Their prominence suggests that they function as “generalist” medicinal plants, supporting the management of a wide range of common and severe health conditions.

A second tier of species, including *S. longepedunculata* and *A. indica*, also displayed high UV values, reflecting their widespread cultural relevance but with a more disease-focused pattern of use. In particular, *S. longepedunculata* is strongly associated with snakebite, inflammation and ritual-medical practices, while *A. indica* is primarily linked to malaria and febrile illnesses. This differentiation indicates functional specialization within the core medicinal repertoire, where highly valued plants are either broad-spectrum remedies or highly effective disease-specific treatments.

Species such as *M. indica* and *M. inermis* occupied an intermediate UV range, suggesting moderate cultural importance and selective therapeutic application. These plants appear to be important complementary remedies, used either as adjuncts to primary treatments or in specific clinical contexts. Their intermediate UV values may reflect ecological availability, preparation complexity, or competition with more dominant species in the same therapeutic niches.

In contrast, the majority of recorded species exhibited very low UV values, indicating marginal or highly specialized use. Such plants may be associated with rare conditions, family-specific traditions, or knowledge held by a small number of practitioners. Although their limited UV suggests low cultural diffusion, these plants remain ethnobotanically significant, as low-frequency species with documented medicinal use often represent reservoirs of unique bioactive compounds and culturally embedded therapies.

### Inferential Statistical Analysis

**Relationship between Use Value (UV) and Relative Frequency of Citation (RFC)**

A strong and statistically significant positive correlation was observed between Relative Frequency of Citation (RFC) and Use Value (UV) (Spearman’s ρ = 0.85, p < 0.001; Figure 8), indicating that plant species cited more frequently by informants also tended to be associated with a broader range of therapeutic uses. This confirms that RFC is not merely a measure of popularity, but is closely linked to therapeutic versatility within the Talensi ethnomedical system.

**Figure 8.**
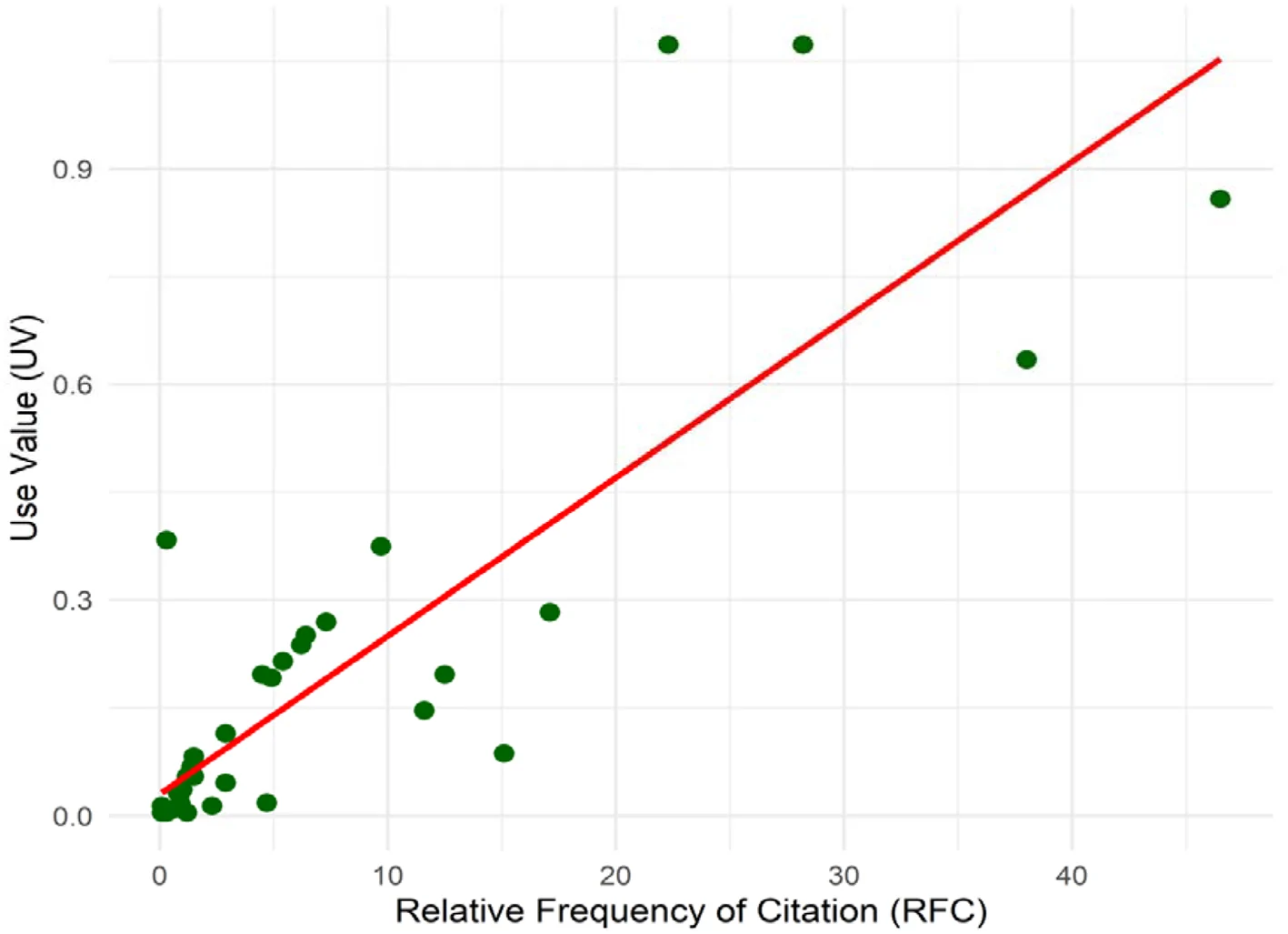
Relationship between Relative Frequency of Citation (RFC) and Use Value (UV) of medicinal plant species recorded in the Talensi District.

The high correlation demonstrates strong internal coherence in the traditional knowledge base: plants that are culturally salient are also those that contribute most extensively to healthcare. Highly cited species such as *S. longepedunculata*, *K. senegalensis*, and *P. kotschyi* therefore function as both cultural and therapeutic keystones within the local pharmacopoeia. Similar positive associations between RFC and UV have been reported in other quantitative ethnobotanical studies, where species with high citation frequencies also show broad therapeutic portfolios, reflecting long-term empirical validation and cultural transmission of knowledge. ^32^ ^,^ ^56^ ^,^ ^57^

### Differences in RFC across Disease Categories

The Kruskal–Wallis test indicated a statistically significant difference in RFC values among the four disease categories (χ ^2^ = 10.23, df = 3, p = 0.0168), demonstrating that medicinal plant use is not evenly distributed across health conditions. Dunn’s post-hoc test with Bonferroni correction ( Table 7, Figure 8) revealed that plants used for gastrointestinal (GI) disorders had significantly higher RFC values than those used for malaria (p = 0.0147) and snakebite (p = 0.0195), while no significant differences were detected among the remaining category pairs.

**Table 7.** Pairwise comparison of RFC among disease categories using Dunn’s post-hoc test (Bonferroni-adjusted).

Comparison	Z-value	Adjusted p-value	Significance
GI vs Malaria	2.81	0.0147	*
GI vs Snakebite	2.72	0.0195	*
GI vs Skin	1.80	0.2155	Ns
Skin vs Malaria	−1.01	0.9320	Ns
Skin vs Snakebite	0.92	1.0000	Ns
Malaria vs Snakebite	−0.09	1.0000	Ns

Significant at p < 0.05; ns = not significant.

The elevated RFC associated with GI-related remedies indicates that gastrointestinal disorders are the most intensively managed conditions within the Talensi traditional healthcare system. This likely reflects their high prevalence, chronic recurrence, and suitability for household-level herbal management. In contrast, the lower RFC values for malaria and snakebite suggest a more restricted ethnomedical domain, possibly due to increasing reliance on biomedical treatment for malaria and the episodic nature of snakebite.

The lack of significant differences involving dermatological conditions suggests a more evenly distributed knowledge base for skin diseases, where remedies are neither highly concentrated nor marginalised. The boxplot ( Figure 9) supports these findings by showing a wider dispersion and higher median RFC for GI-related species compared with the other categories.

**Figure 9.**
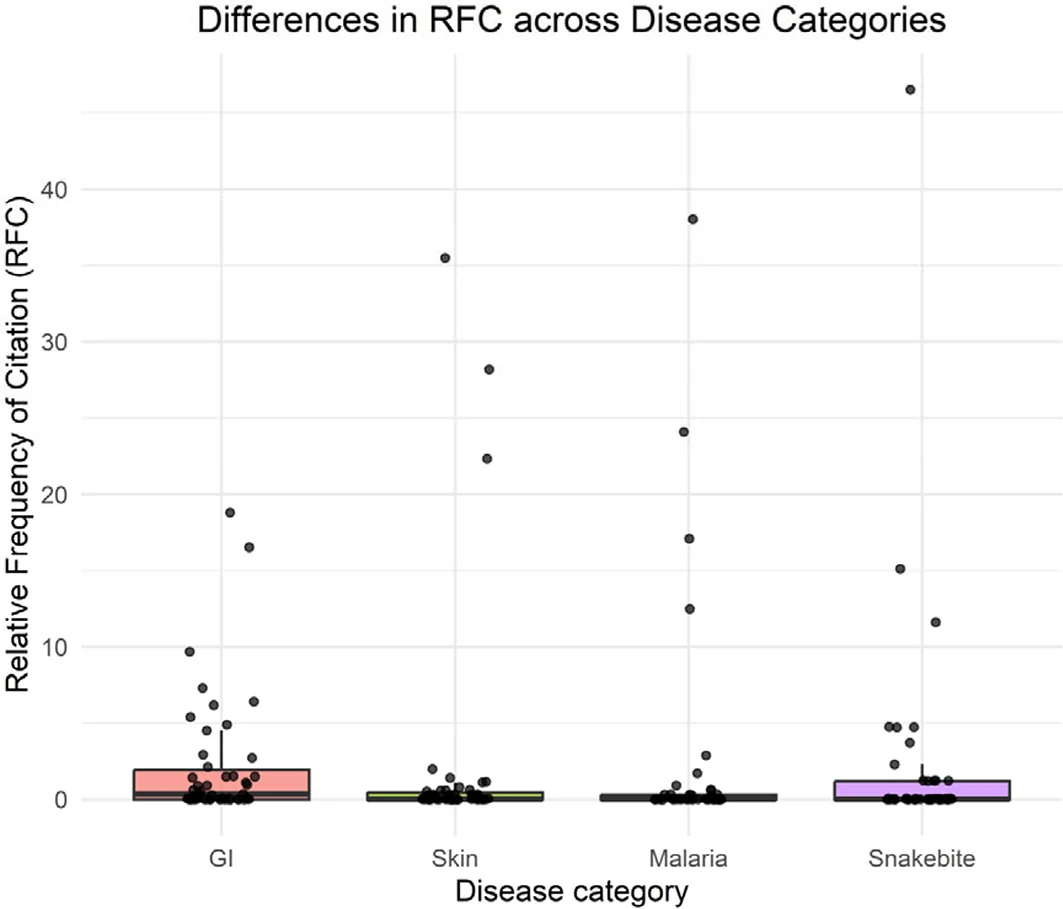
Boxplot showing the distribution of Relative Frequency of Citation (RFC) across disease categories in the Talensi District. Boxes represent median and interquartile range, while points indicate individual plant species.

### Integrated network interpretation (Chord diagram)

To complement the inferential statistical tests, a chord diagram was used to visualise the complete network of interactions between medicinal plant species and disease categories based on RFC values ( Figure 10). While the Spearman correlation quantified the association between RFC and UV, and the Kruskal–Wallis test detected differences among disease categories, the chord diagram provides a structural representation of how ethnobotanical knowledge is organised at the system level. ^56^ ^,^ ^63^

**Figure 10.**
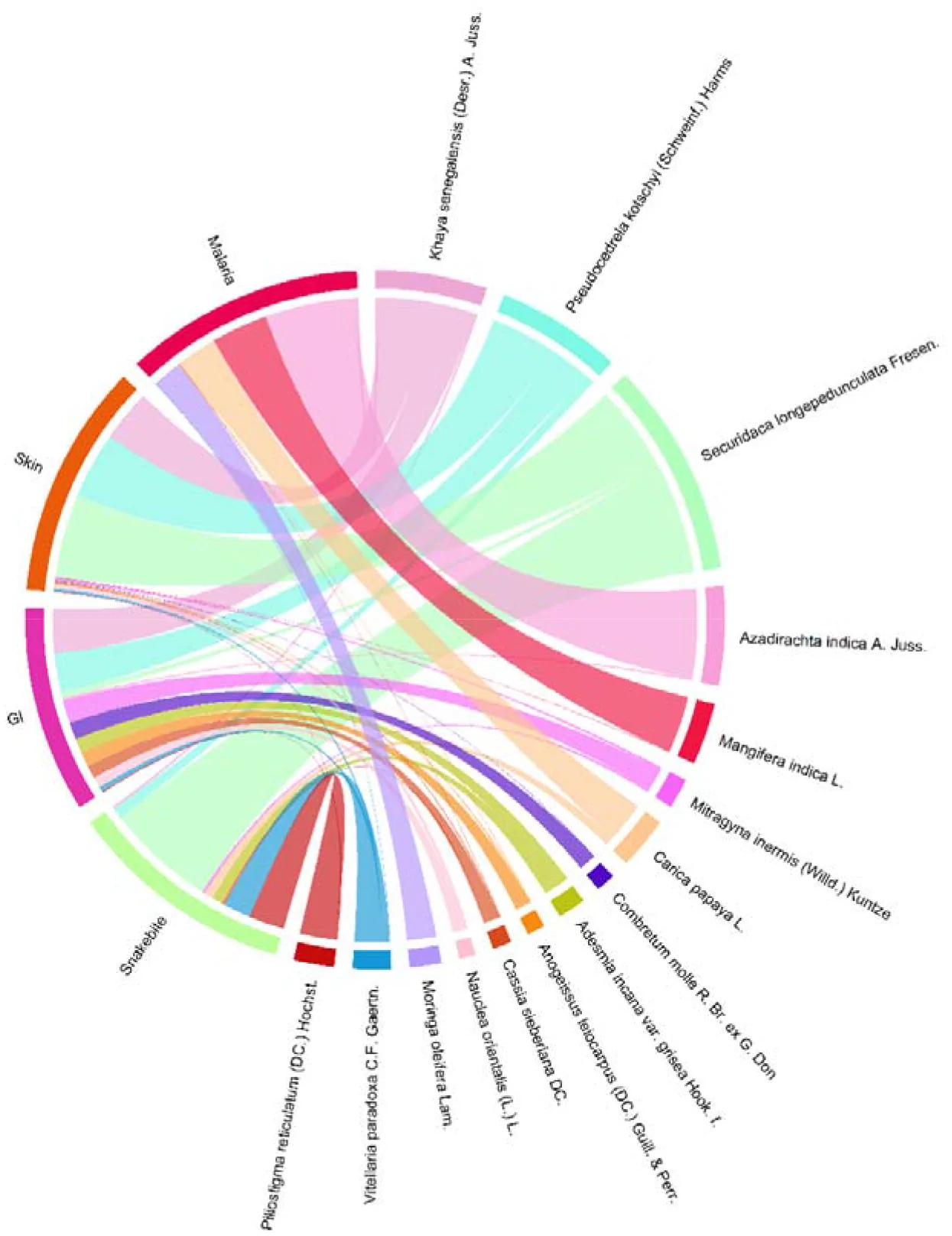
Chord diagram showing associations between the top 15 medicinal plant species and major disease categories in the Talensi District. Ribbon width represents the strength of association based on Relative Frequency of Citation (RFC).

The network reveals that a small number of highly cited species—particularly *S. longepedunculata*, *K. senegalensis*, *P. kotschyi*, and *A. indica*—act as dominant hubs linking multiple disease categories. This hub-and-spoke structure visually reinforces the strong RFC–UV correlation, demonstrating that culturally salient plants are also those that bridge multiple therapeutic domains. ^63^ ^,^ ^64^ In contrast, most species exhibit narrow, disease-specific connections, reflecting specialised or low-frequency use.

The denser network connectivity associated with gastrointestinal remedies mirrors the Kruskal–Wallis and Dunn’s post-hoc results, confirming that GI disorders constitute the most intensively treated category both statistically and structurally.

## Conclusion

This study systematically documented the ethnomedicinal knowledge of the Talensi people of northern Ghana, focusing on plant-based treatments for snakebite, gastrointestinal disorders, dermatological conditions, and malaria. By integrating quantitative ethnobotanical indices with inferential statistical and network analyses, the study provides a robust and reproducible framework for understanding how traditional medical knowledge is structured and transmitted within the community.

The results demonstrate that the Talensi pharmacopoeia is hierarchically organised around a small group of culturally and therapeutically dominant species. *P. kotschyi*, *S. longepedunculata*, *K. senegalensis*, and *A. indica* consistently recorded the highest Use Values and Relative Frequency of Citation, indicating that these plants function as keystone species within the local healthcare system. The strong positive correlation between RFC and UV (Spearman’s ρ = 0.85, p < 0.001) confirms that culturally salient plants are also those with the broadest therapeutic scope, reflecting long-term empirical validation and shared knowledge.

Disease-specific patterns further revealed that gastrointestinal disorders are the most intensively managed category within Talensi traditional medicine, as shown by significantly higher RFC values compared with malaria and snakebite. This statistical pattern, supported by both Kruskal–Wallis and Dunn’s post-hoc tests, suggests that frequent, recurrent, and household-level ailments drive stronger reliance on herbal remedies. High Informant Consensus Factor (ICF) values across all disease categories indicate a well-established and internally coherent ethnomedical system, where plant selection is shaped by collective experience rather than individual preference.

The integrated chord-diagram network analysis reinforced these findings by showing that a small number of multipurpose species connect multiple disease categories, while most plants occupy specialised, low-frequency niches. This network structure demonstrates how cultural importance, therapeutic versatility, and disease burden jointly organise ethnobotanical knowledge in the Talensi District.

Taken together, these results highlight not only the richness of Talensi ethnomedicine but also its internal logic and resilience. The documented species, particularly those occupying central positions in both statistical and network analyses, represent high-priority candidates for future phytochemical and pharmacological investigation. More broadly, this study contributes to the global effort to preserve, validate, and integrate indigenous medicinal knowledge into evidence-based healthcare and drug discovery.

## Declaration statements

### Consent to participate

A written informed consent was obtained from all individual participants included in the study.

### Ethics statement

The questionnaire and methodology for this study was prepared in accordance with the Kwame Nkrumah University of Science and Technology Research Ethics policy and approved by the Committee on Human Research, Publication and Ethics. Ethics approval number: CHRPE/AP/033/22. Approval date: February 2, 2022. The study did not involve clinical trial.

## Data Availability

All data and materials supporting the results and analyses in this paper is published in Harvard Dataverse -view at https://doi.org/10.7910/DVN/QXCKJ0 ^65^ Data are available under the terms of the Creative Commons 1.0 Universal License (CC0 1.0).
