# Supplementary material for: Ethnobotanical Study of Indigenous Knowledge on Medicinal Plants Used in the Talensi District of the Upper East Region of Ghana
Source: Open Res Afr. 2026 May 22;8:16. Originally published 2025 Sep 18. [Version 4] doi: 10.12688/openresafrica.15807.4 (PMC13550910; doi:10.12688/openresafrica.15807.4)
Supplement: Peer review history

# Reviewer report: Reviewer response for version 4

**DOI:** 10.21956/openresafrica.17761.r34707

**Published:** 2026-07-06

**Authors:** Kunal Kaushik

**Affiliations:** 1 Assam Agriculture University, Jorhat, Assam, India

This manuscript presents a valuable ethnobotanical study documenting medicinal plants used in the Talensi–Nabdam area of northern Ghana. The topic is relevant and contributes to the preservation of indigenous medicinal knowledge. Some minor corrections are required

 1. The text mentions both **Talensi and Nabdam districts** and later says the study was designed to document plants used by the **Talensi people**. This should be made consistent. If both districts were included, the wording should reflect that throughout.

 2.The choice of **snakebite, gastrointestinal disorders, dermatological diseases and malaria** should be justified more clearly with evidence of their burden in the study area.

 3.Clarify the knowledge gap more sharply

 4. Acronyms like **RFC, UV, FL, and ICF** should be introduced only after a short explanation of why these indices were selected and what they will help measure

 5. Data collection was conducted from **March to September 2024**, whereas ethical clearance was granted on **9 July 2025**, after the study had already been completed.The authors should clarify whether ethical approval was obtained before data collection or correct the date if it is a typographical error.

 6. Some demographic and climatic information may be excessive for an ethnobotanical study and could be condensed to improve readability

 7. The conclusion that highly cited species are "high-priority candidates for future phytochemical and pharmacological investigation" is reasonable, but the limitations of ethnobotanical indices as predictors of biological activity should be acknowledged.

 8. The discussion would benefit from a clearer comparison of the findings with previous ethnobotanical studies conducted in northern Ghana or neighboring regions to place the results in a broader context.

 9. The authors are encouraged to acknowledge the participation and knowledge-sharing contributions of the local informants and traditional healers involved in this study.

 Overall, the findings are interesting and relevant; however, addressing these points would strengthen the interpretation and improve the scientific rigor of the manuscript.

Is the work clearly and accurately presented and does it cite the current literature?

Yes

If applicable, is the statistical analysis and its interpretation appropriate?

Yes

Are all the source data underlying the results available to ensure full reproducibility?

Partly

Is the study design appropriate and is the work technically sound?

Partly

Are the conclusions drawn adequately supported by the results?

Partly

Are sufficient details of methods and analysis provided to allow replication by others?

Partly

Reviewer Expertise:

Expertise: Horticulture (Vegetable Science), Genetics and Plant Breeding, Ethnobotany, Medicinal and Aromatic Plants, Plant Sciences, Experimental Design, Statistical Analysis and Agricultural Research Methodology. My assessment primarily addresses the study methodology, data analysis, interpretation of findings, and overall scientific quality of the manuscript.

I confirm that I have read this submission and believe that I have an appropriate level of expertise to confirm that it is of an acceptable scientific standard, however I have significant reservations, as outlined above.

# Reviewer report: Reviewer response for version 4

**DOI:** 10.21956/openresafrica.17761.r34665

**Published:** 2026-06-22

**Authors:** Ida Bagus Yorky Brahmantya

**Affiliations:** 1 Medical Education Unit, Universitas Mahasaraswati Denpasar (Ringgold ID: 185821), Bali, Denpasar, Indonesia

The reviewer has examined the latest version of the manuscript against the three points raised in the prior round. **The assessment is mixed: two of the three concerns have been substantively addressed, while one remains only partially resolved.**

 On the first point, updating the literature, the revision is satisfactory. The introduction now draws more explicitly on regional comparative work, and the discussion sections accompanying each disease category (snakebite, gastrointestinal, dermatological, malaria) incorporate additional citations situating the Talensi findings against other ethnobotanical surveys. While the reviewer had specifically flagged three West African sources (Popoola et al. 2022; Assogbadjo et al. 2026; Dossou-Yovo et al. 2022), the authors were not obligated to use exactly these, and the expanded reference list (now including comparative studies from Bangladesh, Pakistan, Italy, and elsewhere) achieves the underlying goal of broadening methodological and geographical context, even if the specific West African citations suggested were not directly taken up. The authors are still encouraged, in a future revision, to anchor at least one or two comparisons specifically within the West African subregion, since the introduction's contextual claims (e.g., regarding religious conversion and knowledge erosion) are supported by citations from Mizoram, Niger, and general Ghana sources rather than studies geographically and ecologically closest to Talensi.

 On the second point, enhancing synthesis, meaningful progress is evident. The revised discussion sections for each disease category (snakebite, stomach-related, skin, malaria) now move beyond restating RFC and FL percentages and instead interpret the coexistence of high-RFC/moderate-FL "generalist" species against low-RFC/high-FL "specialist" species, explicitly linking this to documented antimicrobial, anti-inflammatory, and antiplasmodial properties of species such as Khaya senegalensis, Pseudocedrela kotschyi, and Azadirachta indica. This is a genuine improvement over the previous version's more descriptive tone, and the synthesis in the Use Value section, which frames the RFC–UV correlation as reflecting "cultural and therapeutic keystone" species, is a good example of the kind of interpretive depth previously requested.

 On the third point, clarifying the rationale for the four disease categories, the manuscript now states in the Methods that gastrointestinal disorders, dermatological conditions, snakebite, and malaria were selected based on field observations, informant consensus, prevalence, and alignment with recognized public health challenges, with supporting citations on malaria, snakebite envenomation, and antimicrobial resistance. This directly answers the prior request and is adequately justified.

 One remaining observation, not raised previously, concerns internal consistency in the ethics reporting. The Methods section states that ethical clearance was granted by CHRPE, KNUST, on 9 July 2025 (Approval No. CHRPE/AP/576/25), while the Declaration Statements section at the end of the manuscript cites a different approval number and date (CHRPE/AP/033/22, approved 2 February 2022). This discrepancy should be reconciled, as it bears on the verifiability of ethical oversight for a study involving indigenous knowledge holders, a point of particular importance in ethnobotanical research.

 A minor residual proofreading issue in the demographic discussion ("our findgings," "varifies") should also be corrected at the copyediting stage.

Is the work clearly and accurately presented and does it cite the current literature?

Partly

If applicable, is the statistical analysis and its interpretation appropriate?

Yes

Are all the source data underlying the results available to ensure full reproducibility?

Yes

Is the study design appropriate and is the work technically sound?

Yes

Are the conclusions drawn adequately supported by the results?

Yes

Are sufficient details of methods and analysis provided to allow replication by others?

Yes

Reviewer Expertise:

Ethnomedicine

I confirm that I have read this submission and believe that I have an appropriate level of expertise to confirm that it is of an acceptable scientific standard.

# Reviewer report: Reviewer response for version 4

**DOI:** 10.21956/openresafrica.17761.r34664

**Published:** 2026-06-19

**Authors:** Cecilia S Cordero

**Affiliations:** 1 University of Santo Tomas, Manila, Metro Manila, Philippines; 2 Division of Biological Sciences, University of the Philippines Visayas College of Arts and Sciences, Miagao, Western Visayas, Philippines

Although the manuscript addresses an important ethnobotanical topic and provides valuable documentation of medicinal plant knowledge in the Talensi District, I do not recommend it for indexing in its current form. The manuscript contains substantial concerns regarding the chronology of ethical approval, inconsistencies in the reported data collection period, inappropriate and unsupported citations, and several issues related to data presentation and methodological transparency. In particular, the apparent acquisition of ethical approval after the completion of fieldwork raises concerns about compliance with accepted research ethics standards. Collectively, these issues undermine the credibility and reproducibility of the study and require substantial clarification and revision before the manuscript can be considered for indexing. The following are my additional comments on the paper:

**Major Concerns to be Addressed in the Manuscript**

**1. Temporal Inconsistency in Ethical Approval**

 The manuscript presents inconsistencies regarding the chronology of data collection and ethical approval. Data collection was reported to have occurred during the following periods:

- **February 2023–August 2024** (Abstract)
- **March–September 2024** (Methods)

However, ethical approval was obtained only on **9 July 2025**, suggesting that approval was granted after the completion of fieldwork.

 The authors should clarify:

- Whether preliminary ethical approval was obtained prior to fieldwork;
- Whether retrospective ethical approval was sought and granted; and
- The actual dates of data collection and ethical clearance.

Failure to clarify this issue may raise concerns regarding compliance with research ethics and institutional approval procedures.

**2. Contradictory Dates of Data Collection**

 The reported periods of data collection are inconsistent:

- **Abstract:** February 2023–August 2024
- **Methods:** March–September 2024

A single, accurate sampling period should be consistently reported throughout the manuscript to avoid confusion and ensure transparency.

**3. In-Text Citation and Referencing Issues**

**a.** References **26–28** were cited as sources for the quantitative ethnobotanical indices (ICF, RFC, FL, and UV). However, upon examination, these studies do not appear to have employed these indices in their respective methodologies. The authors should verify the appropriateness of these citations and replace them with references that specifically describe the derivation and application of these indices. Furthermore, the reference list should be revised to conform to the journal's prescribed formatting guidelines.

**b.** The term **“philipines”** should be corrected to **“Philippines.”**

**c.** The authors cite Reference **35** to support the statement that *“Root and bark harvesting can impose significant pressure on slow-growing savanna trees, potentially affecting regeneration dynamics.”* However, the cited study appears to discuss only bark harvesting and does not mention root harvesting. The statement should therefore be revised or supported with an additional reference specifically addressing the impacts of root harvesting.

**4. Clarification and Revisions Required in Table 3**

**a.** Under the mode of administration for *Securidaca longepedunculata* Fresen., the phrase **“Drink infusion and apply past to affected area”** should be clarified. It appears that **“past”** is a typographical error and should likely read **“paste.”**

**b.** For *Piliostigma reticulatum* (DC.) Hochst., the phrase **“Burn together with other non-plant materials”** requires clarification. Please specify the non-plant materials used and provide examples for the benefit of readers.

**c.** Under the “Other Uses” column for *Vitellaria paradoxa* C.F. Gaertn., the term **“kokoo”** may not be familiar to an international readership. A brief description or definition should be provided, either in the table or as a footnote.

**d.** The varietal epithet **“grisea”** in *Adesmia incana* var. *grisea* Hook. f. should be italicized consistently, including in the “Mode of Preparation” column.

**e.** It is concerning that the mode of preparation and mode of administration for several medicinal plants are reported as **“No idea.”** Since these variables were explicitly included in the questionnaire, the authors are encouraged to revisit and verify these information with the informants. If the information was genuinely unavailable, a more appropriate descriptor (e.g., “Not reported” or “Information unavailable”) should be used instead of “No idea.”

Is the work clearly and accurately presented and does it cite the current literature?

Partly

If applicable, is the statistical analysis and its interpretation appropriate?

Partly

Are all the source data underlying the results available to ensure full reproducibility?

Yes

Is the study design appropriate and is the work technically sound?

Yes

Are the conclusions drawn adequately supported by the results?

Yes

Are sufficient details of methods and analysis provided to allow replication by others?

Yes

Reviewer Expertise:

Ethnobotany

I confirm that I have read this submission and believe that I have an appropriate level of expertise to state that I do not consider it to be of an acceptable scientific standard, for reasons outlined above.

# Reviewer report: Reviewer response for version 3

**DOI:** 10.21956/openresafrica.17697.r34284

**Published:** 2026-04-29

**Authors:** Ida Bagus Yorky Brahmantya

**Affiliations:** 1 Medical Education Unit, Universitas Mahasaraswati Denpasar (Ringgold ID: 185821), Bali, Denpasar, Indonesia

**Summary of the Article**

 The manuscript investigates the indigenous knowledge of medicinal plants within the Talensi District of Ghana. The researchers conducted a survey utilizing semi-structured interviews with 219 informants to document traditional remedies for four primary health conditions: gastrointestinal disorders, dermatological infections, snakebites, and malaria. The study successfully identified 46 plant species used in these treatments. To analyze the data, the authors applied several quantitative ethnobotanical indices, including the Informant Consensus Factor (ICF), Fidelity Level (FL), Relative Frequency of Citation (RFC), Plant Part Value (PPV), and Use Value (UV). The findings indicate a high informant consensus for the treatment of gastrointestinal disorders and malaria. Furthermore, the data highlights specific species, such as *Khaya senegalensis* and *Pseudocedrela kotschyi*, as highly significant within the community's traditional pharmacopoeia.

**Assessment of Evaluation Criteria**

**1. Is the work clearly and accurately presented and does it cite the current literature?**

 The manuscript is logically structured, but the integration of current literature requires improvement. The introduction adequately contextualizes the local setting; however, it lacks a robust comparison with recent ethnobotanical studies from neighboring West African regions. Expanding the reference list to include contemporary research that utilizes similar quantitative methodologies will strengthen the theoretical framework. Additionally, while the text is generally clear, the discussion section frequently reiterates data already presented in the results rather than offering a critical synthesis of the findings.

**2. Is the study design appropriate and is the work technically sound?**

 The methodological framework is robust and appropriate for the study's aims. The application of multiple quantitative indices provides a multidimensional evaluation of the ethnobotanical data, moving beyond simple qualitative descriptions. The sample size is well-justified using Cochran's formula, ensuring that the selected knowledge holders adequately represent the targeted demographic.

**3. Are sufficient details of methods and analysis provided to allow replication by others?**

 The authors provide transparent and detailed methodological steps. The processes for plant identification, herbarium verification, and voucher authentication at the Kwame Nkrumah University of Science and Technology are clearly documented. Furthermore, the inclusion of the specific mathematical formulas used to calculate the quantitative indices ensures that the analytical approach can be readily replicated.

**4. If applicable, is the statistical analysis and its interpretation appropriate?**

 The application of the ethnobotanical indices is statistically appropriate for this type of survey data. Calculating the ICF and FL independently for distinct disease categories is a rigorous approach; it prevents the dilution of data and accurately captures the condition-specific fidelity of multi-use plants. The interpretation of the Use Value (UV) correctly reflects the overall cultural significance of the documented species.

**5. Are all the source data underlying the results available to ensure full reproducibility?**

 The authors have demonstrated a commitment to open science by depositing all underlying data and materials in the Harvard Dataverse. The data is accessible under a Creative Commons 1.0 Universal License, fulfilling the requirements for full reproducibility.

**6. Are the conclusions drawn adequately supported by the results?**

 The conclusions logically derive from the presented data. The identification of key medicinal plants and the observed patterns in plant part utilization are directly supported by the calculated quantitative indices. The assertion that documentation preserves cultural heritage and offers pathways for pharmacological investigation is well-founded.

**Points to be addressed:**

1. **Update Literature**: Integrate 3–5 recent peer-reviewed articles focusing on West African ethnobotany to provide a broader geographical and methodological context.
2. **Enhance Synthesis**: Revise the discussion to focus on why certain plants yield high use values or fidelity levels, drawing comparisons to known pharmacological data rather than repeating the raw citation percentages.
3. **Clarify Rationale**: Briefly expand on how the four specific disease categories align with current epidemiological data or public health priorities in Northern Ghana.

Is the work clearly and accurately presented and does it cite the current literature?

Partly

If applicable, is the statistical analysis and its interpretation appropriate?

Yes

Are all the source data underlying the results available to ensure full reproducibility?

Yes

Is the study design appropriate and is the work technically sound?

Yes

Are the conclusions drawn adequately supported by the results?

Yes

Are sufficient details of methods and analysis provided to allow replication by others?

Yes

Reviewer Expertise:

Ethnomedicine

I confirm that I have read this submission and believe that I have an appropriate level of expertise to confirm that it is of an acceptable scientific standard, however I have significant reservations, as outlined above.

## References

[1] : West African medicinal plants and their constituent compounds as treatments for viral infections, including SARS-CoV-2/COVID-19. *DARU Journal of Pharmaceutical Sciences* .2022;30(1) : 10.1007/s40199-022-00437-9 191-210 10.1007/s40199-022-00437-9 PMC904309035476297

[2] : Use patterns and distribution of indigenous knowledge on three Momordica species in Benin, West Africa. *Journal for Nature Conservation* .2026;89: 10.1016/j.jnc.2025.127114 10.1016/j.jnc.2025.127114

[3] : Application of Ethnobotanical Indices in the Utilization of Five Medicinal Herbaceous Plant Species in Benin, West Africa. *Diversity* .2022;14(8) : 10.3390/d14080612 10.3390/d14080612

# Reviewer report: Reviewer response for version 2

**DOI:** 10.21956/openresafrica.17474.r33564

**Published:** 2026-01-28

**Authors:** Cecilia S Cordero

**Affiliations:** 1 University of Santo Tomas, Manila, Metro Manila, Philippines; 2 Division of Biological Sciences, University of the Philippines Visayas College of Arts and Sciences, Miagao, Western Visayas, Philippines

**Ethnobotanical Study of Indigenous Knowledge on Medicinal Plants Used in the Talensi District of the Upper East Region of Ghana**

**Comments, Suggestions, and Recommendations**

**Abstract**

1. Include the inclusive dates of the sampling period.
2. Indicate the total number of medicinal plant species, genera, and families documented in the study area.
3. Highlight the most highly valued medicinal plants, particularly those with high Use Value (UV) or Fidelity Level (FL).
4. Arrange the keywords in alphabetical order.

**Introduction**

1. What is the research gap addressed in this study? Is this the first ethnobotanical study conducted in the Talensi District? If yes, kindly state this clearly in the Significance of the Study. If not, please include and discuss other related ethnobotanical studies conducted in the Talensi District, neighboring districts, or other parts of Ghana to contextualize the contribution of the present study.

**Methods**

1. Include a brief description of the random and snowball sampling techniques, along with appropriate citations, to ensure that readers can clearly understand the sampling methods used in the study.
2. Clarify whether the questionnaire used in this study underwent ethics review. If approved, kindly specify the approving institution. In human-centered studies such as ethnobotanical research, ethics review is particularly important because it protects indigenous knowledge holders, prevents the exploitation of traditional knowledge, and ensures fair acknowledgment and benefit-sharing.

**Results**

1. Why were the respondents’ plant knowledge and demographic parameters not evaluated in this study? It is strongly recommended to include statistical analyses examining the relationship between informants’ plant knowledge and their demographic profiles to determine whether there are significant differences among these parameters.
2. I suggest moving Figures 3a, 3b, and 3c to the appendices.
3. To ensure consistency in figure style, I recommend revising Figures 4 and 5 to match the style of Figure 2, including the font type and size.
4. The following statement is unclear: “ *However, according to the respondents, burning is more of preive method than treating as it thrives mosquitoes away and prevent bite*.” Please restate or paraphrase this sentence for clarity.
5. It is highly recommended to include a subsection on medicinal plant diversity that clearly reports the total number of species, genera, and families documented in the Talensi District. This subsection should also describe the growth habit, conservation status, and endemicity of the recorded plants. Presenting these parameters is important for characterizing floristic composition, assessing ecological patterns, identifying conservation priorities, and highlighting the sustainability and potential vulnerability of medicinal plant resources. Such information strengthens the scientific rigor of the study and supports evidence-based conservation and resource management planning.
6. Include the authority of scientific names, especially at their first mention (e.g., *Securidaca longepedunculata*), including those listed under the “Plants for Snakebite Treatment” section. For subsequent mentions, abbreviate the genus name. Apply this convention consistently throughout the manuscript.
7. **Table 2.** Replace the periods with spaces in the column headings “Category of Diseases” and “Informant Consensus Factor.
8. **Table 3.** Include separate columns for plant parts used, preparation, and mode of administration for plants used in snakebite treatment. Figures 4 and 5 on snakebite preparation and administration are not referenced and do not provide species-specific details; therefore, either properly reference these figures or present the corresponding information for each plant species in the table. Apply the same approach to other disease categories, such as stomach-related diseases and malaria. In addition, specify the actual side effects of medicinal plants rather than indicating only “Yes.” For example, instead of “Yes” for *Securidaca longepedunculata* Fresen., indicate the specific side effects (e.g., dizziness, nausea, or other reported effects).

Is the work clearly and accurately presented and does it cite the current literature?

Partly

If applicable, is the statistical analysis and its interpretation appropriate?

Partly

Are all the source data underlying the results available to ensure full reproducibility?

Yes

Is the study design appropriate and is the work technically sound?

Yes

Are the conclusions drawn adequately supported by the results?

Yes

Are sufficient details of methods and analysis provided to allow replication by others?

Yes

Reviewer Expertise:

Ethnobotany

I confirm that I have read this submission and believe that I have an appropriate level of expertise to confirm that it is of an acceptable scientific standard, however I have significant reservations, as outlined above.

# Response

**Published:** 2026-05-03

**Authors:** Mercy Badu

**Affiliations:** Kwame Nkrumah University of Science and Technology, Ghana

**Response to Reviewer Comments**

 We sincerely thank the reviewer for the detailed evaluation and constructive suggestions provided for our manuscript entitled *“Ethnobotanical Study of Indigenous Knowledge on Medicinal Plants Used in the Talensi District of the Upper East Region of Ghana.”* The comments were highly valuable and have significantly improved the clarity, structure, and scientific rigor of the revised manuscript. Below is our point-by-point response.

**ABSTRACT** **Comment:**

 Include the inclusive dates of the sampling period.

**Response:**

 The Abstract has been revised to include the full sampling period to improve methodological transparency.

**Comment:**

 Indicate the total number of medicinal plant species, genera, and families documented.

**Response:** The revised Abstract now explicitly reports the total number of species, genera, and families identified in the study.

**Comment:** Highlight the most highly valued medicinal plants.

**Response:** The Abstract now briefly mentions the most culturally significant species identified through high Use Value (UV) and Fidelity Level (FL), including *Securidaca longepedunculata*, *Khaya senegalensis*, *Pseudocedrela kotschyi*, and *Azadirachta indica*. .

**INTRODUCTION** **Comment:**

 Clarify the research gap and contextualize the study within previous ethnobotanical research.

**Response:** The Introduction has been revised to clearly define the research gap addressed by this study. We clarified the novelty of documenting medicinal plant knowledge in the Talensi District and expanded the background discussion by referencing related ethnobotanical studies conducted in Ghana and neighbouring regions to strengthen contextualization.

**METHODS** **Comment:**

 Provide clearer explanation of random and snowball sampling techniques.

**Response:**

 A concise methodological description with supporting citations has been added to clarify how random and snowball sampling were combined during participant recruitment.

**Comment:**

 Clarify ethics review.

**Response:**

 Ethical approval details have been added, including the approving institution and informed consent procedures. This ensures compliance with ethical standards for research involving indigenous knowledge holders.

**RESULTS** **Comment:**

 Include statistical evaluation of demographic factors and plant knowledge.

**Response:**

 Additional inferential statistical analyses were incorporated to examine relationships between informants’ demographic characteristics and ethnobotanical knowledge indicators, strengthening the analytical depth of the study.

**Comment:**

 Move Figures 3a–3c to appendices.

**Response:**

 The photographic panels have been relocated to the Appendix section to improve flow and readability of the Results.

**Comment:**

 Standardize figure style.

**Response:**

 Figures 4 and 5 were reformatted to align with the font type, layout, and graphical style of Figure 2.

**Comment:**

 Clarify unclear statement regarding burning practices.

**Response:**

 The sentence has been rewritten to clarify that burning plant material was described by informants primarily as a mosquito-repellent preventive practice rather than a direct therapeutic treatment.

**Comment:**

 Include medicinal plant diversity subsection.

**Response:**

 A new subsection titled **“Medicinal Plant Diversity and Floristic Composition”** has been added. This section reports the total number of species, genera, and families, and discusses growth habits, conservation considerations, and ecological implications of the recorded flora.

**Comment:**

 Include botanical authorities at first mention.

**Response:**

 Scientific names now include taxonomic authorities at first mention, and subsequent references use abbreviated genus names, applied consistently throughout the manuscript.

**Comment:**

 Table 2 formatting corrections.

**Response:**

 Column headings have been revised to remove punctuation errors and improve clarity.

**Comment:**

 Improve Table 3 and species-specific details.

**Response:**

 Tables have been updated to include plant parts used, preparation methods, and modes of administration where appropriate. Reported side effects are now specified explicitly instead of being indicated generically.

# Reviewer report: Reviewer response for version 2

**DOI:** 10.21956/openresafrica.17474.r33159

**Published:** 2025-12-30

**Authors:** Mushtaq Ahmad

**Affiliations:** 1 Quaid-I-Azim University, Islamabad, Pakistan

The article documents important indigenous knowledge on medicinal plants; however, it is only partly clear and accurate due to errors in botanical nomenclature and reliance on limited and outdated literature. The study design and technical approach are weakly justified, with insufficient methodological detail to allow replication. Statistical analyses are inadequately described and interpreted. Source data availability is incomplete, and the conclusions are broadly stated but not fully supported by the results.

Is the work clearly and accurately presented and does it cite the current literature?

Partly

If applicable, is the statistical analysis and its interpretation appropriate?

I cannot comment. A qualified statistician is required.

Are all the source data underlying the results available to ensure full reproducibility?

Partly

Is the study design appropriate and is the work technically sound?

Yes

Are the conclusions drawn adequately supported by the results?

Partly

Are sufficient details of methods and analysis provided to allow replication by others?

Partly

Reviewer Expertise:

Ethnobotany, Ethnomedicine and Ethnoecology etc

I confirm that I have read this submission and believe that I have an appropriate level of expertise to state that I do not consider it to be of an acceptable scientific standard, for reasons outlined above.

# Response

**Published:** 2026-05-03

**Authors:** Mercy Badu

**Affiliations:** Kwame Nkrumah University of Science and Technology, Ghana

**Response to Reviewer Comments**

 We sincerely thank the reviewer for the careful evaluation of our manuscript and for the valuable comments aimed at improving scientific clarity, methodological transparency, and overall rigor. We appreciate the recognition of the importance of documenting indigenous medicinal knowledge and have addressed all concerns through substantial revision of the manuscript. Detailed responses are provided below.

**1. Clarity, botanical nomenclature, and use of current literature** **Reviewer comment:** Errors in botanical nomenclature and reliance on limited or outdated literature affected clarity and accuracy.

**Response:**

 All scientific names were carefully reviewed and standardized according to accepted taxonomic authorities, and inconsistencies were corrected throughout the manuscript, tables, and figure captions. The reference list has been expanded to include more recent ethnobotanical and pharmacological studies to strengthen the scientific context and ensure that current literature supports the discussion.

**2. Justification of study design and technical approach** **Reviewer comment:**

 Study design and methodological justification were weakly presented.

**Response:**

 The Methods section has been revised to provide a clearer rationale for the ethnobotanical approach, including detailed explanations of sampling strategy, participant selection, and data collection procedures. The combined use of semi-structured interviews and quantitative ethnobotanical indices has been clarified to demonstrate alignment with established methodologies in ethnobotanical research.

**3. Methodological detail and reproducibility** **Reviewer comment:**

 Insufficient methodological detail limited reproducibility.

**Response:**

 Additional methodological information has been incorporated, including clearer descriptions of data cleaning, calculation of quantitative indices (RFC, UV, FL, ICF, PPV), and inferential statistical procedures. Software versions and analytical workflows are now explicitly stated in the Data Analysis section to facilitate replication by other researchers.

**4. Statistical analyses and interpretation** **Reviewer comment:**

 Statistical analyses were inadequately described and interpreted.

**Response:**

 Inferential statistical analyses have now been included and described in detail. Spearman’s rank correlation was performed to evaluate relationships between Use Value (UV) and Relative Frequency of Citation (RFC), while non-parametric comparisons among disease categories were conducted using the Kruskal–Wallis test followed by Dunn’s post-hoc analysis. Interpretation of statistical results has been revised to ensure that conclusions are directly supported by the data. An integrated chord diagram was also added to visually synthesize network relationships among plant species and disease categories.

**5. Source data availability and reproducibility** **Reviewer comment:**

 Source data availability was incomplete.

**Response:**

 Data tables and supplementary materials have been reorganized and clarified to ensure transparency of the underlying datasets. Supporting information, including plant images and extended materials, has been moved to appendices to improve accessibility while maintaining clarity within the main text.

**6. Conclusions and alignment with results** **Reviewer comment:**

 Conclusions were broadly stated and not fully supported by results.

**Response:**

 The Conclusion section has been revised to more closely reflect the quantitative findings and inferential analyses presented in the Results. Statements have been refined to avoid overgeneralization and to emphasize evidence-based interpretations derived from the data.

**Overall Revision Outcome**

 In response to the reviewer’s comments, the manuscript has undergone substantial revision, including correction of botanical nomenclature, expansion of recent literature, improved methodological detail, addition of inferential statistical analyses, clarification of data availability, and refinement of the conclusions. We believe these revisions significantly enhance the clarity, rigor, and reproducibility of the study. We are grateful for the reviewer’s thoughtful suggestions, which have greatly improved the quality and scientific robustness of the manuscript.

# Reviewer report: Reviewer response for version 2

**DOI:** 10.21956/openresafrica.17474.r33189

**Published:** 2025-12-15

**Authors:** Miriam Patti

**Affiliations:** 1 Mediterranea University of Reggio Calabria, Reggio Calabria, Italy

The manuscript presents an ethnobotanical study conducted in the Talensi District of Ghana, documenting 46 plant species traditionally used to treat four main categories of health conditions: gastrointestinal disorders, dermatological infections, malaria and snakebites. The study offers a relevant contribution to the preservation of traditional knowledge and provides a rich dataset supported by the use of several quantitative ethnobotanical indices, which represent one of the strongest aspects of the work. This revised version shows clear improvements, especially in the description of the study area, the presentation of voucher specimens, and the justification of the sampling strategy.

 Despite these improvements, several issues remain that require substantial revision before the article can be considered for publication. Some methodological aspects are still not sufficiently clarified, particularly the combination of snowball and random sampling, which is described in a way that does not fully ensure the transparency of data collection. Moreover, the manuscript lacks inferential statistical analyses that could enhance the interpretation of the results. The decision to focus exclusively on four disease categories, even if now justified, remains somewhat restrictive and would benefit from deeper discussion in comparison with recent literature from West Africa.

 The manuscript is also overly long and at times repetitive, with descriptive sections that could be streamlined. The discussion often reiterates information already presented in the results, rather than developing a more critical and integrated interpretation. The language still requires careful revision, and the quality of some figures remains insufficient. Although the reference list has been expanded, it could still benefit from the inclusion of more recent and methodologically relevant studies.

 In this regard, the methodological framing of the manuscript could be strengthened by considering the inclusion of additional references that, although focused on different geographical areas, adopt similar quantitative ethnobotanical approaches and analytical schemes (particularly regarding the combined use of indices such as ICF, RFC, UV and FL). If deemed relevant by the authors, the following DOIs may offer useful methodological parallels:

 (refer to 1, 2, 3)

 Their inclusion should be evaluated solely on the basis of their relevance to the structure and objectives of the manuscript.

 Overall, the manuscript shows potential, but in its current form it does not yet meet the standards required for indexing. Major revisions are necessary, both in terms of methodological clarity and textual organization. For these reasons, my recommendation is that the manuscript **cannot be approved** at this stage, although it may become suitable for reconsideration after substantial revision.

Is the work clearly and accurately presented and does it cite the current literature?

Yes

If applicable, is the statistical analysis and its interpretation appropriate?

No

Are all the source data underlying the results available to ensure full reproducibility?

Partly

Is the study design appropriate and is the work technically sound?

Partly

Are the conclusions drawn adequately supported by the results?

Yes

Are sufficient details of methods and analysis provided to allow replication by others?

Yes

Reviewer Expertise:

Ethnobotany

I confirm that I have read this submission and believe that I have an appropriate level of expertise to confirm that it is of an acceptable scientific standard, however I have significant reservations, as outlined above.

## References

[1] : Ethnobotanical knowledge in Calabria (southern Italy): A summary review. *Heliyon* .2025;11(2) : 10.1016/j.heliyon.2025.e42050 10.1016/j.heliyon.2025.e42050 PMC1179506639911427

[2] : Ethnobotanical survey in the Graecanic Area of Reggio Calabria (Southern Italy): a treasure chest of biodiversity and traditions at risk of extinction. *Ethnobotany Research and Applications* .2025;30: 10.32859/era.30.13.1-29 10.32859/era.30.13.1-29

[3] : The virtues of being peripheral, recreational, and transnational: local wild food and medicinal plant knowledge in selected remote municipalities of Calabria, Southern Italy. *Ethnobotany Research and Applications* .2020;19: 10.32859/era.19.38.1-20 10.32859/era.19.38.1-20

# Response

**Published:** 2026-05-03

**Authors:** Mercy Badu

**Affiliations:** Kwame Nkrumah University of Science and Technology, Ghana

**Response to Reviewer Comments**

 We sincerely thank the reviewer for the detailed and constructive evaluation of our manuscript entitled *“Ethnobotanical Study of Indigenous Knowledge on Medicinal Plants Used in the Talensi District of the Upper East Region of Ghana.”*We appreciate the recognition of the study’s contribution to documenting indigenous knowledge and the strengths noted regarding the quantitative ethnobotanical indices and improved presentation of voucher specimens and study area description. All comments have been carefully considered, and substantial revisions have been implemented to improve methodological clarity, analytical rigor, structure, and language quality. Our detailed responses are provided below.

**1. Clarification of methodological aspects (snowball and random sampling)** **Reviewer comment:**

 The combination of snowball and random sampling lacked transparency.

**Response:**

 The Methods section has been revised to clearly explain the sequential application of sampling strategies. Random sampling was first used to identify communities and initial informants, after which snowball sampling was applied to reach knowledgeable traditional practitioners recommended by participants. Additional procedural details have been added to enhance transparency and reproducibility, including participant eligibility criteria, recruitment flow, and rationale for combining both approaches.

**2. Lack of inferential statistical analyses** **Reviewer comment:**

 The manuscript lacked inferential analyses that enhance interpretation.

**Response:**

 We have now incorporated inferential statistical analyses to strengthen data interpretation. Specifically, Spearman’s rank correlation was used to evaluate the relationship between Relative Frequency of Citation (RFC) and Use Value (UV), and non-parametric comparisons among disease categories were performed using the Kruskal–Wallis test followed by Dunn’s post-hoc analysis with Bonferroni correction. In addition, a chord diagram was included to provide an integrated network-level interpretation of plant–disease relationships. These additions are described in both the Methods and Results sections.

**3. Restriction to four disease categories** **Reviewer comment:**

 The focus on four disease categories appeared restrictive.

**Response:**

 The Introduction and Discussion have been revised to further justify the selection of gastrointestinal disorders, dermatological infections, malaria, and snakebite as focal categories. We clarified that these conditions represent the most frequently reported health concerns during preliminary field observations and reflect regional public health priorities in northern Ghana. Additional discussion has been added comparing our findings with recent ethnobotanical studies from West Africa to contextualize this focused approach.

**4. Length, repetition, and structure of the discussion** **Reviewer comment:**

 The manuscript was overly long and repetitive, with discussion sections reiterating results. **Response:**

 The Results and Discussion sections were extensively streamlined. Descriptive repetition has been reduced, and emphasis has been shifted toward synthesis and interpretation rather than restating numerical findings. Sections were reorganized to improve logical flow, and redundant descriptions of figures were removed. Representative plant photographs were relocated to an appendix to improve readability and reduce manuscript length.

**5. Language revision and figure quality** **Reviewer comment:**

 Language and figure quality required improvement.

**Response:**

 The manuscript has undergone careful language editing to improve clarity, grammar, and academic tone. Figures were regenerated at higher resolution, and graphical elements were standardized. Botanical nomenclature and captions were also revised to ensure consistency with international standards.

**6. Inclusion of recent and methodologically relevant literature** **Reviewer comment:**

 The reference list required expansion with recent studies.

**Response:**

 Additional recent references, including the suggested studies (Patti et al., 2025; Mattalia et al., 2020), were reviewed and incorporated where relevant to strengthen methodological framing and comparative discussion of quantitative ethnobotanical indices such as ICF, RFC, UV, and FL. Literature citations were updated to reflect more current research trends.

**7. Statistical interpretation and reproducibility** **Reviewer comment:**

 Statistical analysis and data transparency needed improvement.

**Response:**

 The Data Analysis section has been expanded to include details of statistical procedures, software versions, and visualization approaches.

**Overall Revision Outcome**

 In response to the reviewer’s recommendations, we have strengthened methodological clarity, added inferential analyses, streamlined the narrative, updated references, improved figures, and enhanced statistical interpretation. We believe these revisions substantially improve the scientific rigor and organization of the manuscript and align it more closely with international indexing standards. We are once again grateful for the reviewer’s insightful feedback, which has significantly contributed to improving the quality of this work.

# Reviewer report: Reviewer response for version 1

**DOI:** 10.21956/openresafrica.16982.r32633

**Published:** 2025-10-27

**Authors:** Ari Sunandar

**Affiliations:** 1 Universitas Muhammadiyah Pontianak, Pontianak, West Kalimantan, Indonesia

Dear Authors;

 The manuscript provides a valuable ethnobotanical documentation of medicinal plants used by the Talensi people in Ghana. The topic is relevant, original, and contributes to the preservation of indigenous knowledge.

 However, the manuscript currently lacks several critical methodological and reporting details that are required to meet international standards for ethnobotanical and ethnopharmacological research.

 Major Comments:

 1. Please include a comprehensive voucher table (species name with authority, family, local name, collector name, voucher number, herbarium acronym, and location).

 2. Please clarify how *use reports* were defined and handled for plants cited under multiple categories.

 3. Please add details about  the rationale for choosing 219 informants.

 4. Please explain the rationale for grouping or limiting to these four (snakebite, gastrointestinal, dermatological, malaria), or include a supplementary table with the complete set of categories analyzed.

 The study is scientifically valuable and has publication potential, but substantial methodological clarification is ****required to meet international ethnobotanical/ethnopharmacological research standards.

Is the work clearly and accurately presented and does it cite the current literature?

Partly

If applicable, is the statistical analysis and its interpretation appropriate?

Partly

Are all the source data underlying the results available to ensure full reproducibility?

No

Is the study design appropriate and is the work technically sound?

Partly

Are the conclusions drawn adequately supported by the results?

No

Are sufficient details of methods and analysis provided to allow replication by others?

No

Reviewer Expertise:

Ethnobotany

I confirm that I have read this submission and believe that I have an appropriate level of expertise to state that I do not consider it to be of an acceptable scientific standard, for reasons outlined above.

# Response

**Published:** 2025-10-27

**Authors:** Mercy Badu

**Affiliations:** Kwame Nkrumah University of Science and Technology, Ghana

All suggestions effected

# Reviewer report: Reviewer response for version 1

**DOI:** 10.21956/openresafrica.16982.r32570

**Published:** 2025-10-08

**Authors:** Adolfo Andrade-Cetto

**Affiliations:** 1 National Autonomous University of Mexico, Mexico City, Mexico

Dear Authors;

 The work presented is of interest; however, upon review, it raises several major concerns:

 •     How was the number of interviewed individuals determined? Does this sample represent the entire study universe of the Talensi District? If so, this must be substantiated.

 •     What is the rationale behind selecting 219 interviewees? Please clarify both the method and justification.

 •     The manuscript lacks the scientific names of the plants and descriptions of the corresponding herbarium vouchers. These are essential for validation.

 •     Regarding the indices used, were they based on vernacular names or scientific nomenclature?

 •     Why are only four categories used for the Informant Consensus Factor (ICF)? Please justify this classification and clarify whether it reflects the full range of reported ailments or was limited by methodological constraints.

 •     How was the Fidelity Level (FL) calculated in cases where a single plant is associated with multiple uses? Please explain how overlapping uses were handled and whether the calculation accounts for dominant or primary indications.

 The manuscript presents potentially valuable insights; however, it requires substantial revisions before it can be considered for acceptance. Key methodological clarifications, taxonomic rigor, and justification of analytical choices are necessary to strengthen its scientific validity and relevance.

 please read; Best practice in research: Consensus Statement on Ethnopharmacological Field Studies – ConSEFS. [Reference 1]

 Recommended standards for conducting and reporting ethnopharmacological field studies [Reference 2]

Is the work clearly and accurately presented and does it cite the current literature?

Partly

If applicable, is the statistical analysis and its interpretation appropriate?

Partly

Are all the source data underlying the results available to ensure full reproducibility?

No

Is the study design appropriate and is the work technically sound?

Partly

Are the conclusions drawn adequately supported by the results?

No

Are sufficient details of methods and analysis provided to allow replication by others?

No

Reviewer Expertise:

Ethnopharmacology, Natural Products, Ethnobotany

I confirm that I have read this submission and believe that I have an appropriate level of expertise to state that I do not consider it to be of an acceptable scientific standard, for reasons outlined above.

## References

[1] : Best practice in research: Consensus Statement on Ethnopharmacological Field Studies – ConSEFS. *Journal of Ethnopharmacology* .2018;211: 10.1016/j.jep.2017.08.015 329-339 10.1016/j.jep.2017.08.015 28818646

[2] : Recommended standards for conducting and reporting ethnopharmacological field studies. *Journal of Ethnopharmacology* .2018;210: 10.1016/j.jep.2017.08.018 125-132 10.1016/j.jep.2017.08.018 28834697

# Response

**Published:** 2025-10-27

**Authors:** Mercy Badu

**Affiliations:** Kwame Nkrumah University of Science and Technology, Ghana

Response to Reviewer 1 Comments

**Review comments** The number of respondents/interviewees and the basis for the chosen sample size were unclear

**Response:** In the revised manuscript, we have provided a detailed explanation of how the sample size was determined and justified (see Section 2.2, Data Collection). The total population of the Talensi District, according to the 2021 Population and Housing Census, is approximately 87,021. Using Cochran’s (1977) sample size determination formula with a 95% confidence level and a 5% margin of error, the theoretical representative sample for the entire adult population would be approximately 382 respondents. However, the present study specifically focused on individuals recognized as knowledge holders—including traditional healers, herbalists, and elderly residents aged 30 years and above—who possess indigenous knowledge of medicinal plants. Therefore, a purposive and snowball sampling approach was adopted, and a total of 219 respondents were interviewed. This number was considered adequate and representative of the knowledge-holding population within the district, rather than of the entire general population. Similar sample sizes have been successfully applied in previous ethnobotanical studies (Martin, 1995; Alexiades, 1996; Giday et al., 2009; Yineger et al., 2008). The revised manuscript now includes these details and references.

**Review Comments** The manuscript lacks the scientific names of the plants and corresponding herbarium voucher information  

**Response:** We have now updated the manuscript to include the correct **scientific names** of all plant species and their corresponding **herbarium voucher numbers**. Initially, plant identification was based on comparison of field photographs with online images; however, after manuscript submission, all collected specimens were taken to the **Herbarium of the Department of Herbal Medicine, Faculty of Pharmacy and Pharmaceutical Sciences, KNUST**, where they were formally identified and assigned voucher numbers. During this verification, two species were corrected: *Mimosa pudica* (previously reported as *Albizia julibrissin*) and *Paullinia pinnata* (previously recorded as *Salacia* sp.). These verified names and voucher numbers are now reflected in **Table 3, Table 4, Table 5 and Table 6** and in the revised **Section 2.3 (Plant Identification and Voucher Authentication)** of the manuscript to ensure taxonomic accuracy and traceability.   These verified names and voucher numbers are now reflected in **Table 3, Table 4, Table 5 and Table 6** and in the revised **Section 2.3 (Plant Identification and Voucher Authentication)** of the manuscript to ensure taxonomic accuracy and traceability.

**Review Comment** Regarding the indices used, were they based on vernacular names or scientific nomenclature?

**Response:** All ethnobotanical indices were calculated using scientifically validated plant names, not vernacular names. Although local (Taleni/Frafra) names were recorded during interviews, each plant specimen was taxonomically identified and verified at the herbarium before data analysis. This approach ensured the avoidance of duplicate entries due to linguistic variation, enhanced accuracy, and maintained scientific consistency across datasets. The method section (Subsection 'Data Analysis') was revised to state that all indices were computed using scientific names confirmed through herbarium identification. The Data Analysis section has been updated to clearly explain how FL and UV were computed, emphasizing the treatment of plants with multiple uses and disease-specific analysis.  

**Review Comment** Why are only four categories used for the Informant Consensus Factor (ICF)? Please justify this classification and clarify whether it reflects the full range of reported ailments or was limited by methodological constraints.  

**Response:** The classification into four main ailment categories, stomach-related, skin-related, malaria, and snakebite was based on preliminary ethnobotanical findings and public health relevance. These categories represent the most commonly reported and culturally significant health issues in the study area and also correspond to globally recognized disease burdens, including snakebite envenomation, malaria, antimicrobial resistance-related skin and gastrointestinal infections, and oxidative stress-related conditions. Thus, the grouping was intentional, context-specific, and data-driven, not due to methodological limitations. The justification for the four ICF categories has been added to the Data Collection and Data Analysis subsections, clarifying that they reflect both local prevalence and global biomedical significance.      

**Review Comments** How was the Fidelity Level (FL) calculated in cases where a single plant is associated with multiple uses? Please explain how overlapping uses were handled and whether the calculation accounts for dominant or primary indications.  

**Response:** In this study, Fidelity Level (FL) was calculated independently for each disease category to capture ailment-specific importance. When a plant was mentioned for multiple uses, each use was analyzed separately within its relevant disease category, while the primary indication—that with the highest number of informant citations—was used for FL computation. For Use Value (UV), however, each plant was considered only once, regardless of repeated mentions, to avoid overestimation. This method ensures that FL values reflect disease-specific fidelity while maintaining statistical rigor.

 References are added or Cited in the Revision.
