# Supplementary material for: Ethnobotanical Study of Indigenous Knowledge on Medicinal Plants Used in the Talensi District of the Upper East Region of Ghana
Source: Open Res Afr. 2026 Apr 10;8:16. Originally published 2025 Sep 18. [Version 3] doi: 10.12688/openresafrica.15807.3 (PMC13550910; doi:10.12688/openresafrica.15807.3)
Supplement: Peer review history

# Response

**Published:** 2025-10-27

**Authors:** Mercy Badu

**Affiliations:** Kwame Nkrumah University of Science and Technology, Ghana

Response to Reviewer 1 Comments

**Review comments** The number of respondents/interviewees and the basis for the chosen sample size were unclear

**Response:** In the revised manuscript, we have provided a detailed explanation of how the sample size was determined and justified (see Section 2.2, Data Collection). The total population of the Talensi District, according to the 2021 Population and Housing Census, is approximately 87,021. Using Cochran’s (1977) sample size determination formula with a 95% confidence level and a 5% margin of error, the theoretical representative sample for the entire adult population would be approximately 382 respondents. However, the present study specifically focused on individuals recognized as knowledge holders—including traditional healers, herbalists, and elderly residents aged 30 years and above—who possess indigenous knowledge of medicinal plants. Therefore, a purposive and snowball sampling approach was adopted, and a total of 219 respondents were interviewed. This number was considered adequate and representative of the knowledge-holding population within the district, rather than of the entire general population. Similar sample sizes have been successfully applied in previous ethnobotanical studies (Martin, 1995; Alexiades, 1996; Giday et al., 2009; Yineger et al., 2008). The revised manuscript now includes these details and references.

**Review Comments** The manuscript lacks the scientific names of the plants and corresponding herbarium voucher information  

**Response:** We have now updated the manuscript to include the correct **scientific names** of all plant species and their corresponding **herbarium voucher numbers**. Initially, plant identification was based on comparison of field photographs with online images; however, after manuscript submission, all collected specimens were taken to the **Herbarium of the Department of Herbal Medicine, Faculty of Pharmacy and Pharmaceutical Sciences, KNUST**, where they were formally identified and assigned voucher numbers. During this verification, two species were corrected: *Mimosa pudica* (previously reported as *Albizia julibrissin*) and *Paullinia pinnata* (previously recorded as *Salacia* sp.). These verified names and voucher numbers are now reflected in **Table 3, Table 4, Table 5 and Table 6** and in the revised **Section 2.3 (Plant Identification and Voucher Authentication)** of the manuscript to ensure taxonomic accuracy and traceability.   These verified names and voucher numbers are now reflected in **Table 3, Table 4, Table 5 and Table 6** and in the revised **Section 2.3 (Plant Identification and Voucher Authentication)** of the manuscript to ensure taxonomic accuracy and traceability.

**Review Comment** Regarding the indices used, were they based on vernacular names or scientific nomenclature?

**Response:** All ethnobotanical indices were calculated using scientifically validated plant names, not vernacular names. Although local (Taleni/Frafra) names were recorded during interviews, each plant specimen was taxonomically identified and verified at the herbarium before data analysis. This approach ensured the avoidance of duplicate entries due to linguistic variation, enhanced accuracy, and maintained scientific consistency across datasets. The method section (Subsection 'Data Analysis') was revised to state that all indices were computed using scientific names confirmed through herbarium identification. The Data Analysis section has been updated to clearly explain how FL and UV were computed, emphasizing the treatment of plants with multiple uses and disease-specific analysis.  

**Review Comment** Why are only four categories used for the Informant Consensus Factor (ICF)? Please justify this classification and clarify whether it reflects the full range of reported ailments or was limited by methodological constraints.  

**Response:** The classification into four main ailment categories, stomach-related, skin-related, malaria, and snakebite was based on preliminary ethnobotanical findings and public health relevance. These categories represent the most commonly reported and culturally significant health issues in the study area and also correspond to globally recognized disease burdens, including snakebite envenomation, malaria, antimicrobial resistance-related skin and gastrointestinal infections, and oxidative stress-related conditions. Thus, the grouping was intentional, context-specific, and data-driven, not due to methodological limitations. The justification for the four ICF categories has been added to the Data Collection and Data Analysis subsections, clarifying that they reflect both local prevalence and global biomedical significance.

**Review Comments** How was the Fidelity Level (FL) calculated in cases where a single plant is associated with multiple uses? Please explain how overlapping uses were handled and whether the calculation accounts for dominant or primary indications.  

**Response:** In this study, Fidelity Level (FL) was calculated independently for each disease category to capture ailment-specific importance. When a plant was mentioned for multiple uses, each use was analyzed separately within its relevant disease category, while the primary indication—that with the highest number of informant citations—was used for FL computation. For Use Value (UV), however, each plant was considered only once, regardless of repeated mentions, to avoid overestimation. This method ensures that FL values reflect disease-specific fidelity while maintaining statistical rigor.

 References are added or Cited in the Revision.
